# New genes in the evolution of the neural crest differentiation program

**DOI:** 10.1186/gb-2007-8-3-r36

**Published:** 2007-03-12

**Authors:** Juan-Ramon Martinez-Morales, Thorsten Henrich, Mirana Ramialison, Joachim Wittbrodt, Juan-Ramon Martinez-Morales

**Affiliations:** 1 Developmental Biology Unit, EMBL, Meyerhofstraße, 69117 Heidelberg, Germany

## Abstract

**Background:**

Development of the vertebrate head depends on the multipotency and migratory behavior of neural crest derivatives. This cell population is considered a vertebrate innovation and, accordingly, chordate ancestors lacked neural crest counterparts. The identification of neural crest specification genes expressed in the neural plate of basal chordates, in addition to the discovery of pigmented migratory cells in ascidians, has challenged this hypothesis. These new findings revive the debate on what is new and what is ancient in the genetic program that controls neural crest formation.

**Results:**

To determine the origin of neural crest genes, we analyzed Phenotype Ontology annotations to select genes that control the development of this tissue. Using a sequential blast pipeline, we phylogenetically classified these genes, as well as those associated with other tissues, in order to define tissue-specific profiles of gene emergence. Of neural crest genes, 9% are vertebrate innovations. Our comparative analyses show that, among different tissues, the neural crest exhibits a particularly high rate of gene emergence during vertebrate evolution. A remarkable proportion of the new neural crest genes encode soluble ligands that control neural crest precursor specification into each cell lineage, including pigmented, neural, glial, and skeletal derivatives.

**Conclusion:**

We propose that the evolution of the neural crest is linked not only to the recruitment of ancestral regulatory genes but also to the emergence of signaling peptides that control the increasingly complex lineage diversification of this plastic cell population.

## Abstract 2

The phylogenetic classification of genes that are ontologically associated with neural crest development reveals that neural crest evolution is associated with the emergence of new signalling peptides.

## Background

As first proposed by Gans and Northcutt [1,2], the major evolutionary innovation of the vertebrate body plan relies on elaboration of a new head at the anterior end of an ancestral chordate trunk. The three existing groups of the phylum Chordata, namely urochordates (ascidians), cephalochordates (amphioxus), and craniates (including vertebrates and agnates), share many characteristics. These include a notochord, segmented trunk muscles, and a dorsal nerve cord. Molecular data have further confirmed these anatomic descriptions, revealing a conserved patterning mechanism along the anterior-posterior and dorso-ventral axes of the neural tube [3]. Resting on this archetypal chordate body plan, unique populations of cells, the neural crest and the ectodermal placodes, evolved in craniates (referred to here as 'vertebrates' for simplicity). The emergence of these pluripotent cells is linked to the evolution of more sophisticated sensory and predatory organs (for instance, jaws). These new organs, in conjunction with an increasingly complex brain, allowed the shift from a filter-feeding style of life toward active predatory strategies [2,4].

The neural crest is a transient population of embryonic cells that originate at the boundary between neural plate and dorsal ectoderm. Secreted from neighboring tissues, signaling molecules of the Wnt, Fgf, and Bmp families cooperate to activate a distinct combination of transcription factors at the neural plate border. Among those are members of the Pax, Zic, Snail, Sox, and Msx families, which constitute the neural crest specification network [5,6]. Shortly after their dorsal specification, neural crest cells undergo an epithelial-to-mesenchymal transition, migrate, and finally, upon arrival at their destination, they give rise to a variety of cell types. These include peripheral neurons, glial and Schwann cells, pigment cells, endocrine cells, cartilage, and bone [7,8]. This large diversity of derivatives arises through a complex mechanism of lineage restriction, which operates both early, on the pluripotent precursors at the dorsal neural tube [9], and later, during the migration and differentiation of precursors already committed to different degrees [10,11]. Environmental cues found throughout neural crest migratory routes play a fundamental role not only in instructing the precursor's differentiation into particular phenotypes, but also in controlling their proliferation and survival [7]. Among these extracellular cues, classical signaling molecules such as Fgfs, Wnts, Bmps and transforming growth factor (TGF)-βs, in conjunction with locally produced cytokines such as neurotropins, endothelins, glial-derived neurotropic factor (GDNF), neuregulin and cKit, have been shown to influence precursor fate and survival [12,13].

The neural crest has traditionally been considered the key structure acquired very early by craniate pioneers. The presence of cartilage first and biomineralized material later in the head of the earliest craniate fossils supports this view [14,15]. Because of their particular nature, the evolution of cartilage and bone elements can easily be traced in the large collection of Cambrian fossils. Many fossil fish exhibit neural crest derived exoskeletal coverings of dermal bone that extend partially over the trunk, with no trace of mesenchymal endoskeleton [16]. These paleontologic records indicate that in early vertebrates cartilage and bones arose first in the context of the cephalic neural crest, and that only later was this genetic program co-opted by the para-axial sclerotome [17].

The existence of an ancestral population of cells in early chordates that give rise to vertebrate neural crest on the one hand and to basal chordate dorsal derivatives on the other has been proposed several times [2,18-20]. This hypothesis is supported by the conservation of many components of the neural crest specification network in chordates [6]. Furthermore, migratory cells that express neural crest markers and differentiate as pigmented cells have recently been identified in the urochordate *Ecteinascidia turbinate *[21]. These data reinforce the hypothesis of pan-chordate 'precursors' behaving similarly and expressing a set of genes homologous to the modern neural crest. According to this view, the innovative drive impelling neural crest evolution stems from the evolution of their *cis*-regulatory elements - a process facilitated by the ancestral duplication of the vertebrate genome. The duplication of key developmental genes would have released enough evolutionary pressure to facilitate their divergence and hence the evolution of new functions [17]. Although the existence of pan-chordate 'precursors' offers a satisfactory answer to the evolutionary origin of the neural crest, it fails to account for the acquisition of fundamental properties of this tissue. These include the pluripotency of the neural crest precursors that now give rise to novel cell types that are present neither in basal chordates nor in other metazoans.

To gain insight into the origin and evolution of neural crest properties, we have chosen a bioinformatics approach to analyze the phylogeny of tissue-specific developmental programs in a systematic manner. Our analytical tool takes advantage of an extensive collection of mouse genes annotated through Mammalian Phenotype Ontology terms [22] (at Mouse Genome Informatics [MGI] [23]). According to their related mouse mutant phenotype annotations, we grouped genes into tissue-specific genetic programs. We then explored the phylogeny of each program using a sequential blast pipeline. We defined as 'new genes' those encoding proteins that did not exhibit any significant homology in previous phylogenetic categories, either because they are extremely divergent or because they have evolved *de novo*. For each group, the total number of new genes at each branch of the evolutionary tree was analyzed. These graphical representations (gene emergence plots) are characteristic for each tissue/organ. They show how the rate of gene innovation has changed during the evolution of a particular tissue. These data substantiate the traditional concept that neural crest is a vertebrate innovation. In addition, our systematic analysis demonstrates that neural crest evolution builds not only on the rewiring of gene networks but also on the emergence of new genes. Gene Ontology (GO) analysis of the group of new neural crest components revealed remarkable enrichment in extracellular ligands. Half of the vertebrate new genes encode secreted cytokines that are known to control the specification and survival of the different neural crest derivatives, including pigment cells, neurons, glial cells, and skeletal components. Here we propose that the emergence of these novel ligands is associated with the evolutionary transition of a relatively simple cell population, in the dorsal neural tube of ancestral chordates, toward the lineage complexity of the vertebrate neural crest.

## Results and discussion

How animal body plans are modified in relation to the evolution of their genome is an intricate issue. Acquisition of novel properties in a particular cell type, or even innovative changes in tissues and organs, can very often be attributed to modifications in the wiring of pre-existing gene networks [24]. However, a fundamental process in genome evolution is also the emergence of new genes. Several molecular mechanisms, including exon shuffling, gene duplication and fusion, transposition, fast sequence divergence, and entire *de novo *origin, have been proposed to serve as sources for gene innovation [25]. In this work we explore the phylogeny of the genes that are involved in neural crest development to gain insight into the evolution of neural crest properties. We aimed to determine which components of the vertebrate neural crest gene program are ancient, and hence have been recruited to perform a function in this tissue, and which components evolved only recently.

### Determining the origin of vertebrate proteins through a sequential blast pipeline

**Figure 1:**
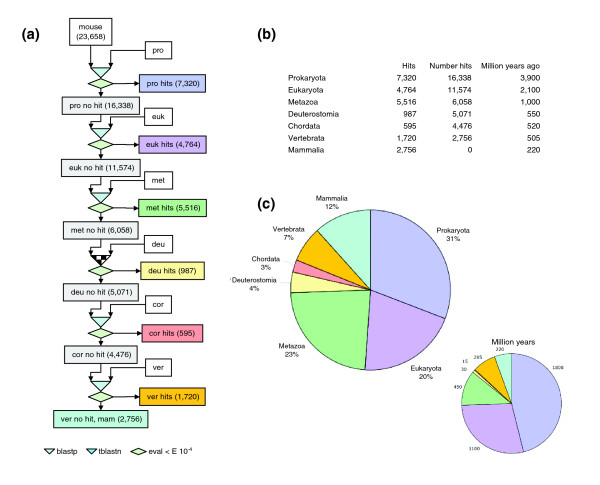
Gene phylogeny was explored using a sequential blast pipeline. **(a) **All known mouse proteins were sequentially blasted (cutoff value E = 10^-4^) against available databases and then classified according to their appearance into seven different categories: prokaryota (pro), eukaryota (euk), metazoa (met), deuterostomia (deu), chordata (cor), vertebrata (ver), and mammalia (mam). **(b) **The table shows the number of mouse genes assigned to each category compared with their estimated age in millions of years. **(c) **Graphical representation of the global gene phylogeny.

As a first step in determining when neural crest genes evolved, we filtered mouse proteins through a sequential blast pipeline. All 23,658 known mouse protein sequences (EnsEMBL v31) were consecutively blasted against available genomes grouped into seven different evolutionary categories (prokaryota, eukaryota, metazoa, deuterostomia, chordata, vertebrata, and mammalia) using a relaxed threshold of E = 10^-4^, as established in similar studies [26,27]. Proteins exhibiting homology when blasted against the prokaryotic genomes were classified as ancient. The remaining genes were subsequently blasted against eukaryotic genomes and the procedure was repeated until all genes were classified (Figure 1a). According to our definition, 'new genes' in each category are those encoding proteins that did not exhibit any significant homology in previous categories, either because they have diverged extensively from a former protein or because they have evolved *de novo*.

A direct comparison of the percentage of genes appearing in each category with an estimation of their respective age in millions of years [28] indicated that the frequency of gene emergence is higher for late categories (specifically, metazoans to mammals; Figure 1b,c). This higher frequency of innovation correlates with the reported observation that the rate of evolution for proteins (calculated as the ratio between nonsynonymous and synonymous amino acid substitutions) is also higher for more recent categories [26].

To elucidate whether 'new proteins', because of their divergent amino acid sequences, correlate with the emergence of novel molecular functions, we performed a GO analysis [29]. For each evolutionary category we identified the GO terms that are statistically over-represented compared with all of the known mouse proteins. The 10 most significantly over-represented GO terms for each of the seven different categories are listed in Table 1 (also see Additional data file 1 for a full list of over-represented GO terms). Our analysis shows that, within a large evolutionary window, innovations are associated with the emergence of 'new genes'. Although the first category, prokaryota, is enriched in genes that are involved in general cell metabolism, GO terms of genes appearing first in eukaryotes demonstrate their function in the newly evolved subcellular organelles. In metazoans we find the GO terms 'cell communication', 'signal transduction', and 'receptor activity' to be highly over-represented, which is in accordance with a *de novo *requirement for cell-cell communication and tissue subspecialization in the context of multicellularity. Interestingly, the collection of genes appearing first in vertebrates and mammals is enriched in terms such as 'hormone activity', 'receptor binding', 'extracellular space', and 'cytokine response', suggesting that diversification of receptor ligands is linked to vertebrate evolution. In summary, our sequential blast pipeline reliably classifies genes according to their first appearance within the phylogenetic tree.

**Table 1:** Frequency of GO terms for each group of 'new genes'

GO ID	GO term	Count sample	Count total	*P*
Prokaryota				
GO:0050875	Cellular physiological process	3,219	8,198	0
GO:0008152	Metabolism	2,576	5,906	0
GO:0044237	Cellular metabolism	2,369	5,566	0
GO:0044238	Primary metabolism	2,192	5,312	0
GO:0043170	Macromolecule metabolism	1,569	3,298	0
GO:0044260	Cellular macromolecule metabolism	1,158	2,500	0
GO:0019538	Protein metabolism	1,149	2,486	0
GO:0044267	Cellular protein metabolism	1,138	2,469	0
GO:0000166	Nucleotide binding	1,070	1,577	0
GO:0016787	Hydrolase activity	1,037	1,876	0

Eukaryota				
GO:0005622	Intracellular	1,820	6,664	0
GO:0043226	Organelle	1,587	5,789	0
GO:0043229	Intracellular organelle	1,586	5,785	0
GO:0043227	Membrane-bound organelle	1,419	5,097	0
GO:0043231	Intracellular membrane-bound organelle	1,417	5,092	0
GO:0005634	Nucleus	1,054	3,267	0
GO:0046914	Transition metal ion binding	644	1,791	0
GO:0008270	Zinc ion binding	619	1,416	0
GO:0004888	Transmembrane receptor activity	23	2,007	0
GO:0043169	Cation binding	799	2,589	3.45 × e^-85^

Metazoa				
GO:0016020	Membrane	1,768	6,163	0
GO:0031224	Intrinsic to membrane	1,524	4,932	0
GO:0016021	Integral to membrane	1,523	4,930	0
GO:0007154	Cell communication	1,234	3,201	0
GO:0007165	Signal transduction	1,211	3,059	0
GO:0004872	Receptor activity	1,143	2,793	0
GO:0007166	Cell surface receptor linked signal transduction	1,061	2,253	0
GO:0004888	Transmembrane receptor activity	926	2,007	0
GO:0007186	G-protein coupled receptor protein signaling pathway	906	1,763	0
GO:0004930	G-protein coupled receptor activity	870	1,693	0

Deuterostomia				
GO:0004931	ATP-gated cation channel activity	5	6	4.74 × e^-05^
GO:0009607	Response to biotic stimulus	45	979	0.00100739
GO:0006952	Defense response	44	950	0.00100739
GO:0004800	Thyroxine 5'-deiodinase activity	3	3	0.002093473
GO:0030106	MHC class I receptor activity	5	15	0.002209497
GO:0006955	Immune response	35	736	0.002495027
GO:0030178	Negative regulation of Wnt receptor signaling pathway	4	9	0.003585659
GO:0042981	Regulation of apoptosis	16	246	0.003971402
GO:0008430	Selenium binding	6	29	0.004113225
GO:0008517	Folic acid transporter activity	3	4	0.004113225

Chordata				
GO:0005911	Intercellular junction	38	131	5.96 × e^-33^
GO:0005921	Gap junction	20	24	1.97 × e^-29^
GO:0030054	Cell junction	38	164	2.28 × e^-29^
GO:0005922	Connexon complex	17	18	2.57 × e^-27^
GO:0005243	Gap-junction forming channel activity	17	18	2.57 × e^-27^
GO:0015285	Connexon channel activity	17	18	2.57 × e^-27^
GO:0005923	Tight junction	17	60	2.44 × e^-14^
GO:0016327	Apicolateral plasma membrane	17	76	1.45 × e^-12^
GO:0043296	Apical junction complex	17	76	1.45 × e^-12^
GO:0005615	Extracellular space	74	2,021	7.43 × e^-10^

Vertebrata				
GO:0005102	Receptor binding	130	507	0
GO:0016503	Pheromone receptor activity	59	111	0
GO:0005179	Hormone activity	53	115	0
GO:0042221	Response to chemical stimulus	90	329	9.81 × e^-79^
GO:0009628	Response to abiotic stimulus	92	414	2.94 × e^-59^
GO:0005615	Extracellular space	230	2,021	1.24 × e^-45^
GO:0005550	Pheromone binding	50	94	1.49 × e^-38^
GO:0005125	Cytokine activity	52	212	5.02 × e^-38^
GO:0005549	Odorant binding	50	99	3.45 × e^-37^
GO:0001664	G-protein-coupled receptor binding	36	47	3.23 × e^-36^

Mammalia				
GO:0005615	Extracellular space	198	2,021	6.14 × e^-53^
GO:0005102	Receptor binding	80	507	1.79 × e^-46^
GO:0005125	Cytokine activity	48	212	1.79 × e^-46^
GO:0009607	Response to biotic stimulus	104	979	1.03 × e^-30^
GO:0006952	Defense response	102	950	1.03 × e^-30^
GO:0042742	Defense response to bacteria	34	70	2.51 × e^-28^
GO:0009617	Response to bacteria	34	78	2.22 × e^-26^
GO:0005126	Hematopoietin/interferon-class (D200-domain) cytokine receptor binding	20	33	6.10 × e^-19^
GO:0008083	Growth factor activity	26	141	2.98 × e^-18^
GO:0051707	Response to other organism	60	594	1.67 × e^-15^

The table summarizes the 10 most statistically overrepresented Gene Ontology (GO) annotations for genes belonging to each of the seven categories. We only considered GO terms for which *P *> 0.001 and count sample was above 15.

### Assignment of neural crest genes based on phenotypic data

In order to investigate when neural crest genes arose during evolution, it was necessary to build a comprehensive list of genes involved in the development of this tissue. A large number of studies, in particular the phenotypic analysis of mutations in mice, generated by either mutagenesis or genetic engineering, have led to the identification of many genes that are involved in neural crest development [7]. The Mammalian Phenotype Browser, at MGI [23], provides a comprehensive resource of phenotypic information derived from mouse mutant studies [22]. Because phenotypic analysis annotations offer the most reliable read out of gene function, we took advantage of this large collection of mouse mutants in our study. The collection includes more than 14,000 genotype records associated with a total of 6,442 genes (27% of the total mouse transcriptome), and furthermore it includes the majority of the genes demonstrated to play a *bona fide *role in neural crest development. In the MGI database each mutation is annotated by a controlled vocabulary of phenotypic terms that describe the effect of a genetic variation on different tissues, organs, or systems. We selected the Mammalian Phenotype Ontology for terms associated with mutations affecting both neural crest precursors and its derivative cell types and tissues.

At the Mammalian Phenotype Browser the ontology term 'abnormal neural crest cells' (MP:0002949:) is reserved for phenotypes that affect the early migration of neural crest cells. Because of this stringent definition, only eight genes are included in this definition. However, when we took phenotypes associated with the development of neural crest derivatives into account, we retrieved a comprehensive list of 615 genes. In our analysis we considered three main groups of neural crest derivatives: pigmented cells, skeletal components, and elements of the peripheral nervous system. The 'pigmentation derivatives phenotype' is completely covered by a single term, namely 'pigmentation phenotype' (MP:0001186). The 'bone derivatives phenotype' terms consist of 'craniofacial phenotype' (MP:0005382) and 'skeleton phenotype' (MP:0005390). At this point, it could be argued that vertebrate neural crest cells only give rise to cranial skeleton and teeth, whereas the axial skeleton has a mesodermal origin. As already mentioned, however, paleontologic records indicate that skeletal elements evolved within the context of the neural crest and only later was this genetic program co-opted by the sclerotome [17]. The 'peripheral nervous system derivatives phenotype' consists of 'abnormal autonomic nervous system morphology' (MP:0002751), 'abnormal peripheral nervous system glia' (MP:0001105), 'abnormal somatic sensory system morphology' (MP:0000959), and 'peripheral nervous system degeneration' (MP:0000958). We grouped these three categories under the general term 'neural crest derivatives phenotype'.

### Determining the origin of the neural crest gene set: gene emergence rate plots

The sequential blast pipeline provides a list of genes that emerge along the evolutionary tree in each of the seven defined categories, whereas the phenotypic annotation provides a functional link for each of these genes. Combining both, we determined in which category each of the 615 neural crest genes emerged (see Additional data file 2 for the full dataset). Previous studies had promoted the idea that gene co-option was the driving force for neural crest invention [6]. Our data strongly support this view because the majority (91%) of genes involved in neural crest development was already present in basal metazoans or even before. Thus, key transcription factors acting as both 'neural plate border specifiers' (such as Pax3, Dlx5, Zic, and Msx1/2) and 'neural crest specifiers' (such as FoxD, Snail/Slug, Sox9/10, Twist, and AP-2) can be traced back to our category 'metazoans' or 'eukaryotes'. Similarly, the Fgf, Wnt, and Bmp signaling pathways involved in induction of the neural plate border are ancestral. Although their corresponding ligands can be traced back to basal metazoans, the kinase activity of their receptors was already present in prokaryotes. Altogether, these data confirm the idea that gene recruitment played an important role during neural crest evolution.

**Figure 2:**
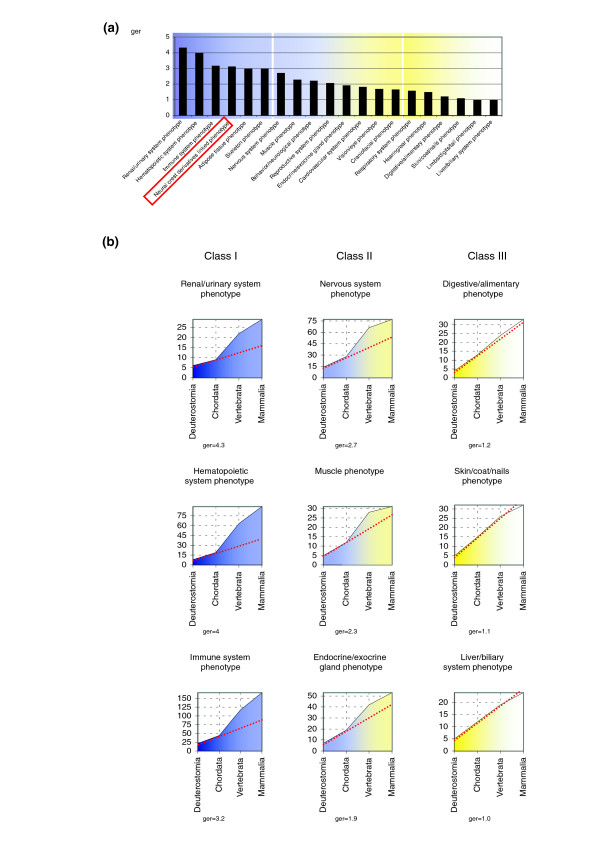
Tissue-specific profiles of gene emergence. The accumulative number of emerging genes (y-axis) in the deuterostomia-mammalia evolutionary window (x-axis) is represented for different tissue-specific genetic programs. We termed these representations gene emergence plots. At the chordate-vertebrate transition the rate of gene emergence (ger) was estimated for the different genetic programs. **(a) **Using mouse phenotypic annotations we calculated ger values between chordata and vertebrata for each main phenotype structure in the database. Structures are highlighted from blue to yellow, according to decreasing values of ger. Neural crest derivative structures are present within the highest ger values (red box). **(b) **Plots of representative structures of each class of ger value: class I = ger > 3; class II = 3 > ger > 1.5; and class III: ger < 1.5.

However, we found that a substantial percentage of the genes (9%, listed in Table 2) involved in neural crest development evolved in deuterostomes during the past 550 million years. To determine, within this evolutionary window, how the rate of gene emergence in the neural crest relates to the rate of innovation in other tissues, we plotted the cumulative number of genes appearing in each category. In these graphs, the tissue-specific evolutionary profile of gene emergence is depicted (Figure 2). In order to quantify the profile of the graphs we calculated 'gene emergence rate' (ger) values, as a numeric representation of the gene innovation rate from an earlier category to a later one (see Materials and methods for a description of the formula). A ger value of 1 indicates a constant profile of gene innovation. Higher ger values indicate increased appearance of new genes in a particular tissue.

**Table 2:** Neural crest genes compiled using Phenotype Ontology annotations (phenotypic information derived from mutant mice studies)

Group	Gene
Deuterostomia	Brain derived neurotrophic factor
	Fanconi anemia, complementation group A
	Fos-like antigen 2
	Neurotropin 3
	Noggin
	Purinergic receptor P2X, ligand-gated ion channel, 7
	Rod outer segment membrane protein 1

Vertebrata	BCL2-like 11 (apoptosis facilitator)
	Calcitonin/calcitonin-related polypeptide, alpha
	Cocaine and amphetamine regulated transcript
	Endothelin 1
	Endothelin 3
	Formin 1
	Glial cell line derived neurotrophic factor
	Gonadotropin releasing hormone 1
	Hermansky-Pudlak syndrome 6
	Integrin, alpha 10
	Islet amyloid polypeptide
	Leukocyte cell derived chemotaxin 1
	Matrix Gla protein
	Melanoma inhibitory activity 1
	Myelin protein zero
	Natriuretic peptide precursor type C
	Neuregulin 1
	Neurturin
	Parathyroid hormone
	Parathyroid hormone-like peptide
	Phosphodiesterase 6G, cGMP-specific, rod, gamma
	Pro-opiomelanocortin-alpha
	Silver
	Tenomodulin
	Treacher Collins Franceschetti syndrome 1, homolog

Chordata	Activating transcription factor 4
	Cbp/p300-interacting transactivator, with Glu/Asp-rich carboxy-terminal domain, 2
	Claudin 14
	Epilepsy, progressive myoclonic epilepsy, type 2 gene alpha
	Fos-like antigen 1
	Gap junction membrane channel protein beta 6
	Hyaluronan and proteoglycan link protein 1
	Transforming growth factor, beta receptor III

Mammalia	Adrenocortical dysplasia
	Ameloblastin
	Amelogenin X chromosome
	BH3 interacting domain death agonist
	Colony stimulating factor 2 (granulocyte-macrophage)
	Harakiri, BCL2 interacting protein (contains only BH3 domain)
	Kit ligand
	Leptin
	Matrix extracellular phosphoglycoprotein with ASARM motif (bone)
	MyoD family inhibitor
	Nonagouti
	Oncostatin M
	Programmed cell death 1
	TYRO protein tyrosine kinase binding protein

The first appearance of neural crest genes was then determined using the sequential blast pipeline (Figure 1). The table contains the complete name of neural crest genes emerging in deuterostomia, chordata, vertebrata and mammalia.

**Figure 3:**
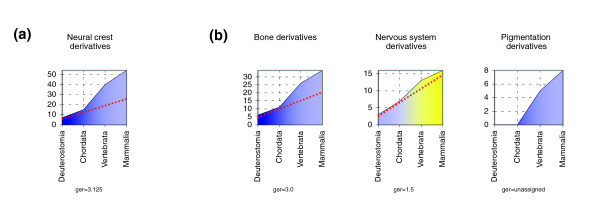
Gene emergence plots of neural crest derivatives. Graphs and gene emergence rate (ger) values associated both with **(a) **the total collection of neural crest genes and **(b) **the different bone, nervous system, and pigmentation derivatives.

For each of the tissue-specific gene programs studied, we ordered the ger values at the chordate-vertebrate transition (Figure 2a). Notably, tissues/systems ontogenetically derived from ventral mesoderm, and hence considered modern vertebrate innovations [2,17,30,31], such as the hematopoietic, immune, or renal/urinary system, exhibit graphs that peak at the chordate-vertebrate transition (Figure 2b). In contrast, other tissues already present in all chordates, namely the epidermis or endodermal derivatives such as liver, respiratory, and digestive systems, have a flat profile, with lower ger values (Figure 2b). Both the profile of the neural crest gene emergence plot (Figure 3) and its ger value (3.1) indicate that the neural crest is among the most innovative vertebrate tissues (Figure 2a). This concept can be extended to each individual neural crest lineage, in particular to pigmented or bone derivatives, as deduced from their respective gene emergence plots (Figure 3). Interestingly, compared with the other crest derivatives, the ger value of the gene set associated with the peripheral nervous system derivatives is lower (1.6). This may best be explained by co-option from the ancestral program of neural development. In summary, our gene emergence plots that reliably reflect evolutionary innovation highlight the novelty of neural crest as a tissue.

### Emergence of neural crest molecules defining novel cellular functions

The notion of neural crest as a tissue with a high rate of gene innovation apparently contradicts our finding that all known neural crest specifiers can be traced back at least to metazoans. To further address this point, we focused on the collection of neural crest 'new genes' to gain insight into their molecular nature and function.

Neural crest has been postulated as a fourth germ layer [32]. This concept builds on neural crest pluripotency and the fact that in vertebrates it gives rise to novel cell types such as the skeletal derivatives or the specialized melanocytes [11]. Consistently, in the collection of vertebrate/mammalian new genes, we found molecules defining the physiology of these novel cell types. This is the case for the genes *Ru *(Hermansky-Pudlak syndrome 6) and *silver*, which encode components of the specialized melanocyte lysosomes, the melanosomes. Similarly, several new genes encode extracellular proteins that constitute part of the bone matrix (for example, bone gla protein and the phosphoglycoprotein mepe) and enamel, the outermost covering of teeth and the hardest tissue in the body (for example, ameloblastin and amelogenin).

### Emergence of ligands for neural crest lineage specification

Strikingly, 50% of neural crest genes appearing first in vertebrates encode extracellular ligands. This remarkable enrichment (confirmed by exploring GO term frequency; see Additional data file 3) is in accordance with our previous whole-transcriptome GO analysis (Table 1). It suggests that diversification of receptor ligands played an important role during vertebrate evolution in general and neural crest evolution in particular. Individual analysis of the function of these peptides during the development of the neural crest demonstrates that they control the commitment of precursors to the different lineages.

Conserved signaling pathways have an early influence on the phenotypic diversification of premigratory neural crest cells [13]. Bmp2/4 can directly induce autonomic neurogenesis [33,34], while Wnt signaling participates in melanocyte specification [35]. Superimposed on this, a second network of 'modern' vertebrate specific cytokines, produced locally, acts not only in neural crest cell fate specification but also in the migratory behavior and survival of all neural crest lineages [12]. Melanocyte specification and survival depend on soluble proteins such as steel factor (kit ligand), endothelin-3, α-melanocyte stimulating hormone, and nonagouti [36]; gliogenesis in the peripheral nervous system is controlled by neuregulins and endothelin-3 [37,38]; the development of autonomic and sensory neurons is controlled by neurothropins (brain-derived neurotropic factor, neurothropin-3, and neurothropin-4) and GDNF family members (GDNF and neurturin) [39,40]; and, finally, the differentiation of the skeletal lineage is specified by endothelin-1 [41]. Our sequential blast pipeline analysis shows that the vast majority (9/11) of the above-mentioned cell fate specification ligands emerged in vertebrates or, to a lesser extent (steel factor and nonagouti), in mammals.

Interestingly, the blast pipeline uncovered a positive hit in the echinoderm *Strongylocentrotus purpuratus *genome for the neurotropin family members brain-derived neurotropic factor and neurothropin-3. Because it has been proposed that neurotropins constitute a vertebrate innovation [42], we performed a ClustalX alignment [43] of mouse neurotropins against the echinoderm sequence ( Additional data file 4). This revealed that the particular array of cysteines conserved in all neurotropins, the so-called 'cysteine knot' [44], is also present in the echinoderm sequence and therefore identifies it as a putative growth factor. However, the limited amino acid identity (33%) and the lack of conservation in critical residues required for neurotropin binding to Trk receptors indicate that the echinoderm neurotropin-related protein cannot be considered a *bona fide *neurotropin. This suggests that neurotropins evolved from divergent ligands present in ancestral chordates. In fact, the example of neurotropins may be just part of a more general mechanism because other 'new cytokines' can be related to pre-existing growth factors. Supporting this view, GDNF and neurturin are divergent members of the TGF-β superfamily of ligands, as indicated by their particular cysteine knot and hence folding [44]. Similarly, despite their limited homology, neuregulins belong to the epidermal growth factor superfamily of ligands [45].

**Figure 4:**
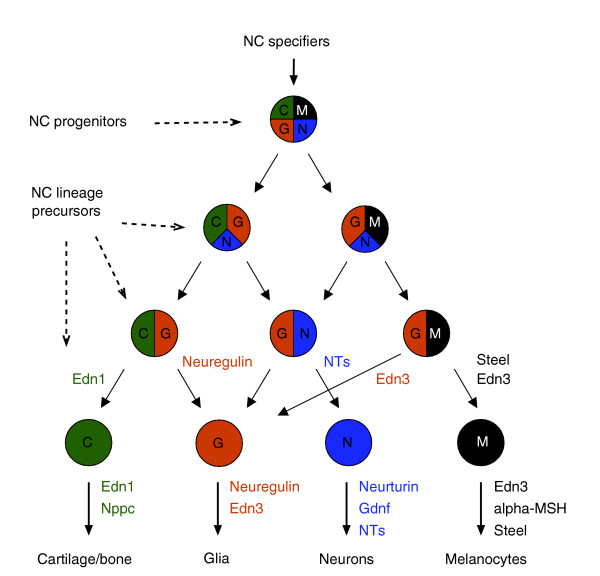
Emerging ligands control the specification of neural crest precursors. The progressive determination of neural crest (NC) precursors into different cell lineages is represented in the scheme with a code of colors. Superimposed on this, the collection of new growth factors appearing first in vertebrates is depicted. The role of each ligand in controlling the specification/survival of each particular neural crest derivative is indicated with a corresponding code of colors. alpha-MSH, alpha-melanocyte-stimulating hormone; End, endothelin; GDNF, glial-derived neurotropic factor; NT, neurotropin; Nppc, natriuretic peptide precursor.

Taken together, our data show that the cytokine network acting in neural crest cell fate specification is mainly a vertebrate innovation (Figure 4). Furthermore, these analyses indicate that an important proportion of the 'new ligands' are derived from fast evolving growth factors.

### Phylogenetic analysis of the emergence of Pfam domains

The comparative analysis of gene emergence plots highlights a high rate of gene innovation for the neural crest during vertebrate evolution. In fact, there are reasons to believe that our estimation on the rate of gene emergence may be conservative. In the sequential blast pipeline analysis, the presence of an ancestral conserved domain will shadow the appearance of evolutionarily more recent domains within the same molecule. This may be particularly relevant in the case of large multidomain proteins such as receptors.

To overcome this constraint and to complement our studies, we conducted a phylogenetic analysis of the Pfam motifs (defined by multiple alignment of proteins [46]) occurring in the collection of 615 neural crest genes. From a total of 8,183 Pfam domains annotated in EnsEMBL, 499 are present in the set of 615 neural crest genes. We screened for these motifs in the seven different categories, detecting homology through two different approaches: blasting Pfam consensus sequences (threshold of E = 10^-4^) and searching for hidden Markov models (HMMs) using HMMER software with standard parameters [46]. We compiled a table including all neural crest genes with their Pfam domains and when they occur first in the defined seven temporal classes, as detected using either of the methods (Additional data file 5). A list including only those genes that contain a Pfam domain emerging in vertebrates is compiled in Table 3. Pfam domain detection supports and refines our sequential blast pipeline results. Thus, GDNF and neurturin were identified as divergent members of the TGF-β superfamily, and the kit-ligand and nonagouti domains were detected as vertebrate novelties (previously detected as mammalian innovations; Table 2). Furthermore, the analysis also confirmed the ClustalX alignments demonstrating that the neurotropin domain (nerve growth factor; Table 3) is indeed a vertebrate innovation. In summary, our domain-based approach (more sensitive and accurate, but limited to annotated Pfam domains) complements the sequential blast analysis (Table 2), providing independent confirmation of the emergence in vertebrates of growth factors that are involved in the specification/survival of the neural crest cells (Table 3).

**Table 3:** Neural crest associated Pfam domains emerging in vertebrates

			Group						
			
Symbol	Gene	blast	pro	euk	met	deu	chr	ver	mam
Slc12a6	Solute carrier family 12, member 6	pro	AA_permease	-	-	-	-	KCl_Cotrans_1	-
Apc	Adenomatosis polyposis coli	pro	-	Arm	APC_crr APC_15aa	-	-	EB1_binding APC_basic SAMP	-
Asph	Aspartate-beta-hydroxylase	pro	Asp_Arg_Hydrox	-	-	-	-	Asp-B-Hydro_N	-
Top2b	Topoisomerase (DNA) II beta	pro	DNA_topoisoIV DNA_gyraseB HATPase_c	-	-	-	-	DTHCT	-
Nef3	Neurofilament 3, medium	pro	-	-	Filament	-	-	Filament_head	-
Nefl	Neurofilament, light polypeptide	pro	-	-	Filament	-	-	Filament_head	-
Cryab	Crystallin, alpha B	pro	HSP20	-	-	-	-	Crystallin	-
Rabggta	Rab geranylgeranyl transferase, a subunit	pro	LRR_1	LRR_2 PPTA	-	-	-	RabGGT_insert	-

Otx1	Orthodenticle homolog 1	euk	-	Homeobox	-	-	-	TF_Otx	-
Otx2	Orthodenticle homolog 2	euk	-	Homeobox	-	-	-	TF_Otx	-
Zfp98	Zinc finger protein 98	euk	zf-C2H2	-	-	-	-	SCAN	-

Prph1	Peripherin 1	met	-	-	Filament	-	-	Filament_head	-
Gfra1	Glial cell line derived neurotrophic factor family receptor alpha 1	met	-	-	-	-	-	GDNF	-
Cdx1	Caudal type homeo box 1	met	-	Homeobox	-	-	-	Caudal_act	-
Cdx2	Caudal type homeo box 2	met	-	Homeobox	-	-	-	Caudal_act	-
Hoxb9	Homeo box B9	met	-	Homeobox	-	-	-	Hox9_act	-
Hoxa9	Homeo box A9	met	-	Homeobox	-	-	-	Hox9_act	-
Nr3c1	Nuclear receptor subfamily 3, group C, member 1	met	-	-	Hormone_recep zf-C4	-	-	GCR	-
Pdgfa	Platelet derived growth factor, alpha	met	-	-	PDGF	-	-	PDGF_N	-

Bdnf	Brain derived neurotrophic factor	deu	-	-	-	-	-	NGF	-
Ntf3	Neurotropin 3	deu	-	-	-	-	-	NGF	-
P2rx7	Purinergic receptor P2X, ligand-gated ion channel, 7	deu	-	-	-	-	-	P2X_receptor	-

Hapln1	Hyaluronan and proteoglycan link protein 1	cor	-	-	V-set	-	-	Xlink	-

Nppc	Natriuretic peptide precursor type C	ver	-	-	-	-	-	ANP	-
Calca	Calcitonin/calcitonin-related polypeptide, alpha	ver	-	-	-	-	-	Calc_CGRP_IAPP	-
Iapp	Islet amyloid polypeptide	ver	-	-	-	-	-	Calc_CGRP_IAPP	-
Cart	Cocaine and amphetamine regulated transcript	ver	-	-	-	-	-	CART	-
Edn1	Endothelin 1	ver	-	-	-	-	-	Endothelin	-
Edn3	Endothelin 3	ver	-	-	-	-	-	Endothelin	-
Nrg1	Neuregulin 1	ver	I-set	EGF	ig V-set	-	-	Neuregulin	-
Pomc1	Pro-opiomelanocortin-alpha	ver	-	-	-	-	-	Op_neuropeptide ACTH_domain	-
Pthlh	Parathyroid hormone-like peptide	ver	-	-	-	-	-	Parathyroid	-
Pth	Parathyroid hormone	ver	-	-	-	-	-	Parathyroid	-
Pde6g	Phosphodiesterase 6G, cGMP-specific, rod, gamma	ver	-	-	-	-	-	PDE6_gamma	-

a	Nonagouti	mam	-	-	-	-	-	Agouti	-
Osm	Oncostatin M	mam	-	-	-	-	-	LIF_OSM	-
Kitl	Kit ligand	mam	-	-	-	-	-	SCF	-

The table summarizes a list of those neural crest genes having at least one Pfam domain appearing first in vertebrates. All the corresponding Pfam domains of these genes, when these domains have appeared, and the classification of the genes according to our previous sequential blast analysis (blast) are indicated. cor, chordata; deu, deuterostomia; euk, eukaryota; mam, mammalia; met, metazoa; pro, prokaryota; ver, vertebrata.

In addition, the domain-based approach also detected 'new Pfam motifs' masked in the sequential blast pipeline studies by the presence of an ancient domain. An example is the appearance in vertebrates of regulatory domains, such as TF_Otx, caudal_act, and Hox9_act, which are present in homeobox-containing transcription factors that belong to the Otx, Cdx, and Hox9 families, respectively (Table 3). We have shown that half of the neural crest genes appearing first in vertebrates encode extracellular ligands. This is contrasted by the Pfam domain analysis of the corresponding receptors. Only a single domain in ligand receptors is identified as a vertebrate novelty, namely the GDNF domain, which is present in the GDNF and neurturin coreceptor termed GFRalpha-1. This observation suggests that receptor evolution requires only subtle changes (in the sequence of their extracellular domains) to allow interaction with the 'new ligands', changes that are too subtle to be detected as discrete 'new domains' in our analysis.

### Final remarks: toward a comprehensive hypothesis on neural crest evolution

Our understanding of how developmental regulatory pathways evolved in metazoans is now building upon steadily accumulating sequence collections that cover representative taxonomic groups. Here we have developed and applied a bioinformatics approach that allows us first to define components of the neural crest developmental gene program and then to analyze their phylogeny. Our evolutionary study, as for others based on comparative genomics, is limited by the quality of the available resources. The validity of the conclusions, beyond individual evolutionary relationships among genes, arise from the global picture provided by the properties of large datasets in which no systematic bias has been introduced. In our study we have considered several potential sources of bias. An important limitation in comparative studies is the arbitrary definition of the components of a particular gene network or gene program. Often this definition is directly inferred from the literature [23]. To avoid this, the phenotypic analysis of mutants offers the most reliable read out of gene function, and at the same time it provides an unbiased definition. The fact that 'less conspicuous' phenotypic features, such as phenotypes associated with the immune or hematopoietic system, are as well annotated as the more obvious ones in pigmentation or skin indicates that there is no global bias in our analysis toward the detection of a given phenotype. Another possible caveat when interpreting studies of this type may come from massive gene loss in sister phyla, which will result in the false impression of new genes emerging in the phylum considered. These losses are particularly apparent in protostomia [47]. In our analysis, focused on deuterostomia groups, these effects are well buffered by filtering the data not only through tunicates but also through echinoderm and cephalochordate sequences. The fact that it is highly unlikely that the same gene is independently lost in all three phylogenetic branches levels potential bias through gene loss and gives robustness to our approach.

Our data show that new genes, either resulting from gene divergence or *de novo *gene evolution, are linked to the appearance of novel molecular and cellular functions. Comparative study of different tissues shows the highest gene emergence rates for those tissues considered vertebrate innovations, such as neural crest and ventral mesoderm derivatives [17]. For the neural crest gene program, we show that half of the genes appearing first in vertebrates encode growth factors with a reported role in committing precursor fate (Figure 4). Our whole-genome analysis also shows that GO terms such as 'hormone activity', 'receptor binding', 'extracellular space', and 'cytokine response' are highly enriched in the collection of genes that emerge in vertebrates. Therefore, the expansion of the ligand toolkit during evolution does not appear to be limited to the neural crest. Rather, it also occurred in other vertebrate-specific tissues, which evolved from ancestral chordates. Examples of this are the vertebrate-specific interleukins and hematopoietic cytokines that control fate, maturation, and survival of the complex lymphoid and blood cell lineages. Taken together, our data indicate that the appearance of new growth factors satisfied an evolutionary requirement for signal diversification, beyond the ancestral network of signaling peptides.

The diversification of ancestral ligands may represent an independent evolutionary advantage distinct from a parallel diversification of receptors. The independent evolution of derived growth factor genes, now under the control of divergent promoter sequences, introduces additional complexity in the spatial and temporal regulation of signaling. Divergent ligands may also interact very differently with extracellular matrix components, with concomitant changes in gradient shape and presentation to the receptors. Ligands with those new properties can now take advantage of existing receptors and downstream signaling pathways that are present in competent cells. This further refinement of the activity of growth factors may be particularly important in lineage-rich tissues, in which it is crucial to discriminate inductive signals involved in cell fate determination.

Previous theories on neural crest evolution have mainly focused on the ontogenetic and phylogenetic origin of the tissue from the dorsal area of the ancestral chordate neural tube. Along these lines, the rewiring of the genes involved in the neural crest specification network has been invoked as the main evolutionary driving force [6]. Our data now expand this view by suggesting that new signaling molecules were required to control further the development of the neural crest into its different derivatives, which are essential components of the actual vertebrate body plan.

## Materials and methods

### Blast searches and assignment of temporal categories

To estimate the emergence of mammalian genes we analyzed the set of all 23658 known mouse protein sequences from EnsEMBL v31. We identified any gene product related to the mouse reference sequences in 225 different genomes by using blast [48]. A complete list of genomes and their origin is given in supplementary information (Additional data file 6). Genomes were downloaded from Cogent, EnsEMBL, and NCBI. These genomes were grouped into seven temporal categories based on their evolutionary origin (Table 4).

**Table 4:** Temporal categories of downloaded genomes

Group	Name	Genomes	Sequences	Source
Prokaryota	Archaea	21 archaeal	48625 pep	Cogent241
	Bacteria	191 bacterial	568028 pep	Cogent241
Eukaryota	Eukaryota	3 (2 yeast, plasmodium)	16597 pep	Cogent241
Metazoa	Metazoa	3 (2 insect, nematode)	19957 pep	Cogent241
Deuterostomia	Deuterostoma	1 (sea urchin)	527735 nuc	NCBI
Chordata	Urochordata	1 (ciona)	21574 pep	EnsEMBL_v31
	Cephalochordata	1 (branchiostoma)	321472 ests	NCBI
Vertebrata	Vertebrata	3 (fish genomes)	93151 pep	EnsEMBL_v32
Mammalia	Mouse	1 (mouse)	23658 pep	EnsEMBL_v31

est, expressed sequence tag; nuc, nucleotide; pep, peptide.

The first appearance of mouse proteins during evolution was assessed through a sequential blast pipeline using a relaxed cutoff value (E = 10^-4^) and standard parameters (blastp for protein and tblastn for nucleotide databases) to detect homology in more distant species [26,27]. We assigned each of the 23,658 mouse proteins to one of the seven evolutionary categories according to when their first hit occurred (in which taxonomic group). Genes already assigned to a temporal category were excluded from further blast analysis. The remaining genes were then subsequently blasted against the following genomes until eventually all mouse genes were classified.

To account for any possible effect due to mouse-specific gene loss biasing our analysis, we performed the following control. In addition to using the mouse gene set as an input for the sequential blast pipeline, we also launched the filtering process using other vertebrate groups, namely chicken, xenopus, and zebrafish genomes. Independent of the input used, we observed a similar distribution of genes in the various evolutionary categories (Additional data file 7). This finding indicates that there is no evident specific gene loss in the mouse. This necessary control further corroborates the choice of the mouse genome as a representative vertebrate.

### Gene Ontology analysis

We looked for GO terms that were statistically over-represented in our temporal categories. Each of these gene sets was compared with the whole set of GO annotated mouse genes. We used mouse MGI GO annotation available at the GoStat web server [49] for this analysis [29]. GoStat compares the occurrence of each GO term for each different temporal category and for the reference genes, and performs a Fisher's exact test to judge whether the observed difference is significant. A complete list of all over-represented and under-represented GO annotations is provided in Additional data file 1.

### Retrieving phenotype annotations

The list of genes described by phenotype ontology was obtained from the MGI report (3.22 release): MRK_Pheno_Ensembl.rpt [50]. This table represents the MGI marker associations with Phenotype Annotations and EnsEMBL sequence. The main phenotypical categories stored in the Mammalian Phenotype Ontology are the following: adipose tissue phenotype (MP:0005375), behavior/neurologic phenotype (MP:0005386), cardiovascular system phenotype (MP:0005385), craniofacial phenotype (MP:0005382), digestive/alimentary phenotype (MP:0005381), endocrine/exocrine gland phenotype (MP:0005379), hearing/ear phenotype (MP:0005377), hematopoietic system phenotype (MP:0005397), immune system phenotype (MP:0005387), limbs/digits/tail phenotype (MP:0005371), liver/biliary system phenotype (MP:0005370), muscle phenotype (MP:0005369), nervous system phenotype (MP:0003631), pigmentation phenotype (MP:0001186), renal/urinary system phenotype (MP:0005367), reproductive system phenotype (MP:0005389), respiratory system phenotype (MP:0005388), skeleton phenotype (MP:0005390), skin/coat/nails phenotype (MP:0005393), and vision/eye phenotype (MP:0005391).

### Gene emergence rate calculation

In order to quantify the relative change in the number of 'new genes' arising at a given temporal category, we define the gene emergence rate (ger) as the ratio between the number of genes emerging in the analyzed temporal category (vertebrates in our case) and the number of genes emerging in the previous temporal category (chordates in our case). Thus, for the transition from chordates to vertebrates, the ger value is defined as follows:

$ger_{\left(cho=>ver\right)}=\frac{N_{ver}-N_{cor}}{N_{cor}-N_{deu}}$

Where *N*_*deu *_is the cumulative number of 'new genes' at the level of deuterostomes, *N*_*cho *_is the cumulative number of novel genes at the level of chordates, and *N*_*ver *_is the cumulative number of 'new genes' at the level of vertebrates.

### Assignment of Pfam domains to temporal categories through HMM and blast searches

To derive a more detailed view of the evolution of proteins involved in neural crest development, we examined when the protein domains found in the list of neural crest genes are first detectable in our temporal categories. First, we downloaded the Pfam annotations for the identified 615 neural crest genes from EnsEMBL (version 32). Of the total of 8183 Pfam domains, 499 are present (annotated in EnsEMBL) in the set of 615 genes. We downloaded consensus sequences and HMMs for these domains from Pfam (version 19.0) [51].

Pfam domains were searched in the temporal category databases using two methods: blasting the Pfam consensus sequence with an E value threshold of 10^-4 ^and searching HMM using HMMER software [52] applying standard parameters. In general, the HMM search was more sensitive and able to detect a domain earlier than the Pfam consensus blast analysis. For the expressed sequence tag databases, we used only the blast search. A full table, including all neural crest genes with their Pfam domains and their appearance as detected by either of the methods, was compiled (Additional data file 5).

## Additional data files

The following additional data are available with the online version of this paper. Additional data file 1 is a table including a full list of statistically over-represented GO annotations of genes belonging to each of the seven categories (cutoff *P *< 0.001, sample count ≥ 15). Additional data file 2 is a table listing the 615 neural crest genes compiled using Phenotype Ontology annotations for each of the seven temporal categories considered in this work. Additional data file 3 is a table showing statistically over-represented GO annotations of the set of neural crest developmental genes that emerged in vertebrates (cutoff *P *< 0.001). Additional data file 4 shows ClustalX alignment of mouse neurotropins against the echinoderm peptide. Additional data file 5 shows phylogenetic analysis of neural crest Pfam domains emergence through evolution. Additional data file 6 provides a complete list of genomes of species included in this work and their respective sources. Additional data file 7 sequential blast analysis using other vertebrate groups as a control for the gene phylogeny analysis.

## Supplementary Material

Additional data file 1 The table includes a full list of the statistically over-represented GO annotations of genes belonging to each of the seven categories (cutoff *P *< 0.001, sample count = 15). Click here for file

Additional data file 2 The table comprises a full list of the 615 neural crest genes compiled using Phenotype Ontology annotations for each of the seven temporal categories considered in this work: prokaryota (pro), eukaryota (euk), metazoa (met), deuterostomia (deu), chordata (cor), vertebrata (ver), and mammalia (mam). Click here for file

Additional data file 3 The table shows statistically over-represented GO annotations of the set of neural crest developmental genes that emerged in vertebrates (cutoff *P *< 0.001). Click here for file

Additional data file 4 ClustalX alignment of mouse neurotropins against the echinoderm peptide. The comparison reveals a limited amino acid identity. Click here for file

Additional data file 5 Phylogenetic analysis of neural crest Pfam domains emergence through evolution. The table shows a full list of the compiled 615 genes involved in neural crest development and the first appearance of their Pfam domains in the different clades. All the corresponding Pfam domains of these genes, when these domains have appeared, and the classification of the genes according to our previous sequential blast analysis (blast; color-coded) are indicated. Click here for file

Additional data file 6 A complete list of genomes of species included in this work and their respective source is compiled in this table. Abbreviations: arc (archaeobacteria), bac (bacteria), euk (eukaryota), met (metazoa), deu (deuterostomia), cor (chordata), ver (vertebrata). Click here for file

Additional data file 7 As a control of our gene phylogeny analysis, we also run the sequential blast pipeline using other vertebrate groups, namely (chicken, xenopus and zebrafish genomes). The tables show the number or percentage of genes assigned to each evolutionary category. The graphical representation of the gene phylogeny for the four vertebrate species analyzed revealed a very similar gene loss/emergence profile. Click here for file
