# Supplementary material for: New genes in the evolution of the neural crest differentiation program
Source: Genome Biol. 2007 Mar 12;8(3):R36. doi: 10.1186/gb-2007-8-3-r36 (PMC1868935; doi:10.1186/gb-2007-8-3-r36)
Supplement: Additional data file 1 — The table includes a full list of the statistically over-represented GO annotations of genes belonging to each of the seven categories (cutoff P < 0.001, sample count = 15). [file gb-2007-8-3-r36-S1.pdf]

GO ID	GO term	count sample	count total	P-Value
**prokaryota**				
GO:0050875	cellular physiological process	3219	8198	0
GO:0008152	metabolism	2576	5906	0
GO:0044237	cellular metabolism	2369	5566	0
GO:0044238	primary metabolism	2192	5312	0
GO:0043170	macromolecule metabolism	1569	3298	0
GO:0044260	cellular macromolecule metabolism	1158	2500	0
GO:0019538	protein metabolism	1149	2486	0
GO:0044267	cellular protein metabolism	1138	2469	0
GO:0000166	nucleotide binding	1070	1577	0
GO:0016787	hydrolase activity	1037	1876	0
GO:0017076	purine nucleotide binding	993	1436	0
GO:0016740	transferase activity	960	1638	0
GO:0030554	adenyl nucleotide binding	854	1162	0
GO:0005524	ATP binding	823	1126	0
GO:0016772	transferase activity, transferring phosphorus-containing groups	605	952	0
GO:0016301	kinase activity	554	846	0
GO:0016773	phosphotransferase activity, alcohol group as acceptor	459	632	0
GO:0016491	oxidoreductase activity	459	654	0
GO:0006793	phosphorus metabolism	441	658	0
GO:0006796	phosphate metabolism	441	658	0
GO:0004672	protein kinase activity	412	546	0
GO:0016310	phosphorylation	402	550	0
GO:0006468	protein amino acid phosphorylation	374	498	0
GO:0004674	protein serine/threonine kinase activity	367	463	0
GO:0004872	receptor activity	313	2793	0
GO:0050874	organismal physiological process	269	2571	0
GO:0004713	protein-tyrosine kinase activity	256	300	0
GO:0007166	cell surface receptor linked signal transduction	212	2253	0
GO:0004888	transmembrane receptor activity	156	2007	0
GO:0007186	G-protein coupled receptor protein signaling pathway	72	1763	0
GO:0007600	sensory perception	54	1438	0
GO:0004930	G-protein coupled receptor activity	27	1693	0
GO:0001584	rhodopsin-like receptor activity	14	1478	0
GO:0007606	sensory perception of chemical stimulus	6	1231	0
GO:0007608	sensory perception of smell	5	1182	0
GO:0005737	cytoplasm	1314	3087	5,66E-80
GO:0019752	carboxylic acid metabolism	264	367	3,65E-75
GO:0006082	organic acid metabolism	264	367	3,65E-75
GO:0000287	magnesium ion binding	198	250	3,87E-69
GO:0009058	biosynthesis	510	958	3,23E-66
GO:0043283	biopolymer metabolism	916	2067	5,84E-63
GO:0006091	generation of precursor metabolites and energy	307	490	1,44E-62
GO:0005739	mitochondrion	428	784	1,33E-59
GO:0016818	hydrolase activity, acting on acid anhydrides, in phosphorus-con	268	413	1,48E-59
GO:0016817	hydrolase activity, acting on acid anhydrides	268	414	3,36E-59
GO:0016462	pyrophosphatase activity	266	410	4,37E-59
GO:0006810	transport	988	2323	9,60E-57
GO:0017111	nucleoside-triphosphatase activity	247	384	1,49E-53
GO:0008233	peptidase activity	357	642	3,29E-52
GO:0005975	carbohydrate metabolism	218	328	3,59E-51
GO:0016887	ATPase activity	177	244	6,71E-51
GO:0051179	localization	1045	2554	1,06E-49
GO:0051234	establishment of localization	1038	2534	1,28E-49
GO:0005506	iron ion binding	176	247	1,37E-48
GO:0044249	cellular biosynthesis	432	849	8,91E-48
GO:0016829	lyase activity	119	140	1,26E-47
GO:0006508	proteolysis	303	533	4,28E-47
GO:0006811	ion transport	334	609	1,01E-46
GO:0042623	ATPase activity, coupled	163	226	3,19E-46
GO:0009987	cellular process	3459	10723	3,33E-45
GO:0015075	ion transporter activity	339	633	5,23E-44
GO:0008236	serine-type peptidase activity	144	193	5,58E-44
GO:0006807	nitrogen compound metabolism	184	280	7,30E-42
GO:0044255	cellular lipid metabolism	220	360	1,35E-41
GO:0004175	endopeptidase activity	247	425	1,06E-40
GO:0004252	serine-type endopeptidase activity	136	184	1,23E-40
GO:0051186	cofactor metabolism	125	163	1,89E-40
GO:0009308	amine metabolism	174	266	4,23E-39
GO:0006118	electron transport	207	342	4,46E-38
GO:0006629	lipid metabolism	248	442	7,50E-37
GO:0044262	cellular carbohydrate metabolism	152	227	4,11E-36
GO:0015291	porter activity	127	176	6,55E-36
GO:0044248	cellular catabolism	208	352	8,42E-36
GO:0043167	ion binding	1075	2795	9,10E-36
GO:0046872	metal ion binding	1075	2795	9,10E-36
GO:0015290	electrochemical potential-driven transporter activity	127	177	1,82E-35
GO:0006732	coenzyme metabolism	109	142	2,70E-35

GO:0006519	amino acid and derivative metabolism	152	230	5,49E-35
GO:0043412	biopolymer modification	565	1295	2,00E-34
GO:0006812	cation transport	227	404	8,01E-34
GO:0008324	cation transporter activity	256	475	1,24E-33
GO:0006464	protein modification	545	1256	2,82E-32
GO:0006520	amino acid metabolism	124	179	6,82E-32
GO:0015293	symporter activity	77	89	9,71E-32
GO:0009056	catabolism	237	437	1,07E-31
GO:0006631	fatty acid metabolism	98	128	1,62E-31
GO:0016853	isomerase activity	99	130	1,77E-31
GO:0008238	exopeptidase activity	74	85	7,19E-31
GO:0009117	nucleotide metabolism	112	158	2,27E-30
GO:0031420	alkali metal ion binding	108	150	2,40E-30
GO:0004386	helicase activity	91	117	2,98E-30
GO:0015672	monovalent inorganic cation transport	159	260	5,73E-30
GO:0030001	metal ion transport	187	325	8,65E-30
GO:0016614	oxidoreductase activity, acting on CH-OH group of donors	84	106	5,07E-29
GO:0048037	cofactor binding	62	68	3,40E-28
GO:0005386	carrier activity	213	398	3,09E-27
GO:0016616	oxidoreductase activity, acting on the CH-OH group of donors, N	78	98	3,68E-27
GO:0015980	energy derivation by oxidation of organic compounds	89	120	1,16E-26
GO:0006066	alcohol metabolism	122	190	4,98E-26
GO:0016705	oxidoreductase activity, acting on paired donors, with incorporat	73	92	3,19E-25
GO:0008610	lipid biosynthesis	111	169	4,83E-25
GO:0050662	coenzyme binding	53	58	4,03E-24
GO:0042598	vesicular fraction	87	124	6,17E-23
GO:0006092	main pathways of carbohydrate metabolism	67	85	8,62E-23
GO:0005792	microsome	85	121	1,78E-22
GO:0016835	carbon-oxygen lyase activity	51	57	2,75E-22
GO:0008026	ATP-dependent helicase activity	63	80	2,21E-21
GO:0007156	homophilic cell adhesion	59	74	1,60E-20
GO:0019199	transmembrane receptor protein kinase activity	50	58	2,53E-20
GO:0006725	aromatic compound metabolism	68	92	3,77E-20
GO:0008237	metallopeptidase activity	103	166	4,58E-20
GO:0016741	transferase activity, transferring one-carbon groups	90	138	6,47E-20
GO:0009165	nucleotide biosynthesis	77	111	9,08E-20
GO:0006457	protein folding	116	198	2,45E-19
GO:0030955	potassium ion binding	67	92	3,22E-19
GO:0016820	hydrolase activity, acting on acid anhydrides, catalyzing transme	84	128	7,13E-19
GO:0042626	ATPase activity, coupled to transmembrane movement of substa	83	126	7,91E-19
GO:0043492	ATPase activity, coupled to movement of substances	83	126	7,91E-19
GO:0015698	inorganic anion transport	81	122	9,86E-19
GO:0008168	methyltransferase activity	87	135	1,09E-18
GO:0004497	monooxygenase activity	76	112	1,59E-18
GO:0043169	cation binding	931	2589	1,59E-18
GO:0030145	manganese ion binding	72	105	5,44E-18
GO:0006820	anion transport	90	144	7,12E-18
GO:0015370	solute:sodium symporter activity	35	36	9,30E-18
GO:0016836	hydro-lyase activity	42	48	1,46E-17
GO:0016627	oxidoreductase activity, acting on the CH-CH group of donors	36	38	1,95E-17
GO:0019001	guanyl nucleotide binding	151	291	2,24E-17
GO:0044270	nitrogen compound catabolism	40	45	3,12E-17
GO:0009310	amine catabolism	40	45	3,12E-17
GO:0015294	solute:cation symporter activity	36	39	1,29E-16
GO:0005525	GTP binding	147	285	1,43E-16
GO:0051188	cofactor biosynthesis	59	82	1,85E-16
GO:0006813	potassium ion transport	83	133	2,06E-16
GO:0005622	intracellular	2144	6664	2,08E-16
GO:0046483	heterocycle metabolism	44	54	5,53E-16
GO:0005996	monosaccharide metabolism	70	106	5,72E-16
GO:0019318	hexose metabolism	69	104	6,36E-16
GO:0051082	unfolded protein binding	77	122	1,12E-15
GO:0015268	alpha-type channel activity	174	360	1,39E-15
GO:0004177	aminopeptidase activity	34	37	1,49E-15
GO:0005216	ion channel activity	162	330	2,31E-15
GO:0004714	transmembrane receptor protein tyrosine kinase activity	39	46	2,54E-15
GO:0005342	organic acid transporter activity	51	69	4,23E-15
GO:0046943	carboxylic acid transporter activity	51	69	4,23E-15
GO:0004702	receptor signaling protein serine/threonine kinase activity	30	31	4,68E-15
GO:0009063	amino acid catabolism	33	36	5,02E-15
GO:0005244	voltage-gated ion channel activity	83	138	7,03E-15
GO:0015267	channel or pore class transporter activity	180	381	8,75E-15
GO:0008235	metalloexopeptidase activity	38	46	3,60E-14
GO:0004263	chymotrypsin activity	38	46	3,60E-14
GO:0005267	potassium channel activity	68	107	4,36E-14
GO:0006814	sodium ion transport	63	97	8,75E-14
GO:0005783	endoplasmic reticulum	217	489	9,46E-14
GO:0016052	carbohydrate catabolism	46	62	1,01E-13
GO:0044275	cellular carbohydrate catabolism	46	62	1,01E-13

GO:0005777	peroxisome	48	66	1,04E-13
GO:0042579	microbody	48	66	1,04E-13
GO:0004295	trypsin activity	37	45	1,13E-13
GO:0016788	hydrolase activity, acting on ester bonds	242	560	1,29E-13
GO:0000267	cell fraction	183	398	1,74E-13
GO:0006790	sulfur metabolism	40	51	1,97E-13
GO:0006821	chloride transport	43	57	2,66E-13
GO:0019205	nucleobase, nucleoside, nucleotide kinase activity	31	35	3,20E-13
GO:0009108	coenzyme biosynthesis	50	72	7,46E-13
GO:0007264	small GTPase mediated signal transduction	110	212	1,20E-12
GO:0005624	membrane fraction	165	357	2,10E-12
GO:0051213	dioxygenase activity	32	38	2,26E-12
GO:0016702	oxidoreductase activity, acting on single donors with incorporati	32	38	2,26E-12
GO:0044271	nitrogen compound biosynthesis	41	55	2,37E-12
GO:0009309	amine biosynthesis	41	55	2,37E-12
GO:0005275	amine transporter activity	43	59	2,48E-12
GO:0008509	anion transporter activity	54	82	3,12E-12
GO:0006084	acetyl-CoA metabolism	29	33	3,62E-12
GO:0008757	S-adenosylmethionine-dependent methyltransferase activity	47	68	6,02E-12
GO:0016701	oxidoreductase activity, acting on single donors with incorporati	32	39	9,47E-12
GO:0006399	tRNA metabolism	50	75	1,15E-11
GO:0016070	RNA metabolism	150	322	1,20E-11
GO:0005249	voltage-gated potassium channel activity	49	73	1,26E-11
GO:0031402	sodium ion binding	48	71	1,39E-11
GO:0016903	oxidoreductase activity, acting on the aldehyde or oxo group of	29	34	1,74E-11
GO:0003723	RNA binding	180	405	1,76E-11
GO:0046164	alcohol catabolism	37	49	1,86E-11
GO:0016886	ligase activity, forming phosphoric ester bonds	42	59	1,99E-11
GO:0046394	carboxylic acid biosynthesis	41	57	2,01E-11
GO:0016053	organic acid biosynthesis	41	57	2,01E-11
GO:0008652	amino acid biosynthesis	30	36	2,30E-11
GO:0006006	glucose metabolism	51	78	2,31E-11
GO:0046365	monosaccharide catabolism	36	48	5,47E-11
GO:0019320	hexose catabolism	36	48	5,47E-11
GO:0006007	glucose catabolism	36	48	5,47E-11
GO:0046942	carboxylic acid transport	41	58	5,50E-11
GO:0015849	organic acid transport	41	58	5,50E-11
GO:0006633	fatty acid biosynthesis	39	54	5,74E-11
GO:0008202	steroid metabolism	66	113	6,28E-11
GO:0006974	response to DNA damage stimulus	88	166	6,99E-11
GO:0005261	cation channel activity	105	209	7,71E-11
GO:0006281	DNA repair	76	137	7,85E-11
GO:0051540	metal cluster binding	25	28	8,42E-11
GO:0051536	iron-sulfur cluster binding	25	28	8,42E-11
GO:0046356	acetyl-CoA catabolism	22	23	9,27E-11
GO:0009057	macromolecule catabolism	116	238	9,58E-11
GO:0006260	DNA replication	60	100	1,03E-10
GO:0005230	extracellular ligand-gated ion channel activity	44	65	1,13E-10
GO:0006694	steroid biosynthesis	39	55	1,56E-10
GO:0045211	postsynaptic membrane	47	72	1,93E-10
GO:0043229	intracellular organelle	1836	5785	2,16E-10
GO:0043226	organelle	1837	5789	2,23E-10
GO:0009719	response to endogenous stimulus	89	171	2,23E-10
GO:0016798	hydrolase activity, acting on glycosyl bonds	59	99	2,39E-10
GO:0009060	aerobic respiration	21	22	3,16E-10
GO:0006099	tricarboxylic acid cycle	21	22	3,16E-10
GO:0004180	carboxypeptidase activity	30	38	3,31E-10
GO:0006730	one-carbon compound metabolism	25	29	4,12E-10
GO:0051187	cofactor catabolism	25	29	4,12E-10
GO:0004812	tRNA ligase activity	38	54	4,24E-10
GO:0016875	ligase activity, forming carbon-oxygen bonds	38	54	4,24E-10
GO:0016876	ligase activity, forming aminoacyl-tRNA and related compounds	38	54	4,24E-10
GO:0008452	RNA ligase activity	38	54	4,24E-10
GO:0015171	amino acid transporter activity	37	52	4,39E-10
GO:0005328	neurotransmitter:sodium symporter activity	19	19	4,96E-10
GO:0005326	neurotransmitter transporter activity	19	19	4,96E-10
GO:0016337	cell-cell adhesion	64	112	5,52E-10
GO:0009109	coenzyme catabolism	23	26	9,15E-10
GO:0016860	intramolecular oxidoreductase activity	31	41	1,21E-09
GO:0009259	ribonucleotide metabolism	45	70	1,21E-09
GO:0006163	purine nucleotide metabolism	45	70	1,21E-09
GO:0044265	cellular macromolecule catabolism	103	212	1,87E-09
GO:0008094	DNA-dependent ATPase activity	22	25	3,08E-09
GO:0045333	cellular respiration	22	25	3,08E-09
GO:0031072	heat shock protein binding	28	36	3,08E-09
GO:0050660	FAD binding	19	20	3,71E-09
GO:0005489	electron transporter activity	88	175	3,78E-09
GO:0043231	intracellular membrane-bound organelle	1619	5092	5,52E-09
GO:0043227	membrane-bound organelle	1620	5097	6,15E-09

GO:0004759	serine esterase activity	26	33	8,41E-09
GO:0016769	transferase activity, transferring nitrogenous groups	26	33	8,41E-09
GO:0005615	extracellular space	694	2021	8,68E-09
GO:0005343	organic acid:sodium symporter activity	17	17	8,68E-09
GO:0007242	intracellular signaling cascade	277	714	8,86E-09
GO:0016859	cis-trans isomerase activity	27	35	9,31E-09
GO:0004601	peroxidase activity	28	37	1,00E-08
GO:0016684	oxidoreductase activity, acting on peroxide as acceptor	28	37	1,00E-08
GO:0006733	oxidoreduction coenzyme metabolism	21	24	1,01E-08
GO:0015203	polyamine transporter activity	29	39	1,04E-08
GO:0005279	amino acid-polyamine transporter activity	29	39	1,04E-08
GO:0006096	glycolysis	30	41	1,05E-08
GO:0015036	disulfide oxidoreductase activity	18	19	1,25E-08
GO:0006766	vitamin metabolism	35	52	2,14E-08
GO:0016765	transferase activity, transferring alkyl or aryl (other than methyl	35	52	2,14E-08
GO:0016645	oxidoreductase activity, acting on the CH-NH group of donors	19	21	2,18E-08
GO:0015837	amine transport	34	50	2,34E-08
GO:0005509	calcium ion binding	282	735	2,41E-08
GO:0006767	water-soluble vitamin metabolism	26	34	2,76E-08
GO:0003755	peptidyl-prolyl cis-trans isomerase activity	26	34	2,76E-08
GO:0006720	isoprenoid metabolism	17	18	4,33E-08
GO:0019866	organelle inner membrane	115	254	5,22E-08
GO:0004298	threonine endopeptidase activity	18	20	7,32E-08
GO:0046777	protein amino acid autophosphorylation	18	20	7,32E-08
GO:0051287	NAD binding	18	20	7,32E-08
GO:0016877	ligase activity, forming carbon-sulfur bonds	18	20	7,32E-08
GO:0016540	protein autoprocessing	18	20	7,32E-08
GO:0043038	amino acid activation	31	45	7,34E-08
GO:0043039	tRNA aminoacylation	31	45	7,34E-08
GO:0006418	tRNA aminoacylation for protein translation	31	45	7,34E-08
GO:0031966	mitochondrial membrane	110	242	8,40E-08
GO:0005882	intermediate filament	51	90	8,40E-08
GO:0006259	DNA metabolism	184	452	1,02E-07
GO:0009072	aromatic amino acid family metabolism	19	22	1,07E-07
GO:0009064	glutamine family amino acid metabolism	19	22	1,07E-07
GO:0009150	purine ribonucleotide metabolism	39	63	1,29E-07
GO:0045111	intermediate filament cytoskeleton	51	91	1,48E-07
GO:0004518	nuclease activity	70	138	1,52E-07
GO:0009260	ribonucleotide biosynthesis	38	61	1,52E-07
GO:0015276	ligand-gated ion channel activity	48	84	1,57E-07
GO:0005743	mitochondrial inner membrane	104	228	1,69E-07
GO:0009059	macromolecule biosynthesis	216	549	1,77E-07
GO:0016830	carbon-carbon lyase activity	23	30	2,25E-07
GO:0004890	GABA-A receptor activity	24	32	2,38E-07
GO:0016125	sterol metabolism	34	53	2,81E-07
GO:0015296	anion:cation symporter activity	16	17	3,19E-07
GO:0007214	gamma-aminobutyric acid signaling pathway	16	17	3,19E-07
GO:0009069	serine family amino acid metabolism	16	17	3,19E-07
GO:0051539	4 iron, 4 sulfur cluster binding	14	14	3,19E-07
GO:0016667	oxidoreductase activity, acting on sulfur group of donors	14	14	3,19E-07
GO:0019362	pyridine nucleotide metabolism	18	21	3,43E-07
GO:0009975	cyclase activity	18	21	3,43E-07
GO:0008203	cholesterol metabolism	32	49	3,64E-07
GO:0016620	oxidoreductase activity, acting on the aldehyde or oxo group of	20	25	5,44E-07
GO:0006836	neurotransmitter transport	28	41	5,66E-07
GO:0005516	calmodulin binding	52	96	5,70E-07
GO:0006575	amino acid derivative metabolism	38	63	6,01E-07
GO:0019748	secondary metabolism	21	27	6,13E-07
GO:0031012	extracellular matrix	152	368	6,39E-07
GO:0005578	extracellular matrix (sensu Metazoa)	152	368	6,39E-07
GO:0042625	ATPase activity, coupled to transmembrane movement of ions	49	89	6,54E-07
GO:0015934	large ribosomal subunit	23	31	6,83E-07
GO:0016917	GABA receptor activity	24	33	6,85E-07
GO:0006164	purine nucleotide biosynthesis	37	61	7,12E-07
GO:0005839	proteasome core complex (sensu Eukaryota)	16	18	7,74E-07
GO:0006749	glutathione metabolism	16	18	7,74E-07
GO:0016229	steroid dehydrogenase activity	16	18	7,74E-07
GO:0005740	mitochondrial envelope	114	261	7,79E-07
GO:0030574	collagen catabolism	15	16	9,87E-07
GO:0009141	nucleoside triphosphate metabolism	35	57	1,01E-06
GO:0004707	MAP kinase activity	13	13	1,04E-06
GO:0006221	pyrimidine nucleotide biosynthesis	17	20	1,08E-06
GO:0006220	pyrimidine nucleotide metabolism	17	20	1,08E-06
GO:0004553	hydrolase activity, hydrolyzing O-glycosyl compounds	44	78	1,09E-06
GO:0006865	amino acid transport	29	44	1,16E-06
GO:0020037	heme binding	56	108	1,50E-06
GO:0046906	tetrapyrrole binding	56	108	1,50E-06
GO:0004091	carboxylesterase activity	26	38	1,60E-06
GO:0016712	oxidoreductase activity, acting on paired donors, with incorporat	24	34	1,84E-06

GO:0016776	phosphotransferase activity, phosphate group as acceptor	21	28	1,89E-06
GO:0008483	transaminase activity	21	28	1,89E-06
GO:0004527	exonuclease activity	31	49	1,92E-06
GO:0006800	oxygen and reactive oxygen species metabolism	31	49	1,92E-06
GO:0016746	transferase activity, transferring acyl groups	70	145	2,26E-06
GO:0016810	hydrolase activity, acting on carbon-nitrogen (but not peptide) b	43	77	2,35E-06
GO:0008415	acyltransferase activity	66	135	2,68E-06
GO:0003995	acyl-CoA dehydrogenase activity	14	15	3,08E-06
GO:0006752	group transfer coenzyme metabolism	31	50	4,04E-06
GO:0016849	phosphorus-oxygen lyase activity	17	21	4,33E-06
GO:0046456	icosanoid biosynthesis	17	21	4,33E-06
GO:0007155	cell adhesion	183	469	4,45E-06
GO:0005057	receptor signaling protein activity	42	76	5,04E-06
GO:0004181	metallocarboxypeptidase activity	19	25	5,37E-06
GO:0004722	protein serine/threonine phosphatase activity	20	27	5,54E-06
GO:0016747	transferase activity, transferring groups other than amino-acyl g	66	137	5,64E-06
GO:0008408	3'-5' exonuclease activity	15	17	5,65E-06
GO:0005773	vacuole	64	132	6,16E-06
GO:0016485	protein processing	28	44	6,51E-06
GO:0031980	mitochondrial lumen	32	53	6,64E-06
GO:0005759	mitochondrial matrix	32	53	6,64E-06
GO:0019201	nucleotide kinase activity	13	14	9,70E-06
GO:0000323	lytic vacuole	57	115	9,76E-06
GO:0005764	lysosome	57	115	9,76E-06
GO:0009152	purine ribonucleotide biosynthesis	33	56	1,05E-05
GO:0006769	nicotinamide metabolism	15	18	1,11E-05
GO:0004089	carbonate dehydratase activity	15	18	1,11E-05
GO:0006396	RNA processing	106	249	1,12E-05
GO:0015405	P-P-bond-hydrolysis-driven transporter activity	51	100	1,12E-05
GO:0006833	water transport	11	11	1,13E-05
GO:0042044	fluid transport	11	11	1,13E-05
GO:0005372	water transporter activity	11	11	1,13E-05
GO:0043449	alkene metabolism	16	20	1,32E-05
GO:0006261	DNA-dependent DNA replication	21	30	1,44E-05
GO:0015082	di-, tri-valent inorganic cation transporter activity	21	30	1,44E-05
GO:0042440	pigment metabolism	17	22	1,46E-05
GO:0019842	vitamin binding	31	52	1,52E-05
GO:0009144	purine nucleoside triphosphate metabolism	31	52	1,52E-05
GO:0019200	carbohydrate kinase activity	19	26	1,54E-05
GO:0006690	icosanoid metabolism	19	26	1,54E-05
GO:0046914	transition metal ion binding	597	1791	1,74E-05
GO:0005976	polysaccharide metabolism	26	41	1,83E-05
GO:0044264	cellular polysaccharide metabolism	26	41	1,83E-05
GO:0016407	acetyltransferase activity	28	46	2,67E-05
GO:0005829	cytosol	112	270	2,70E-05
GO:0004222	metalloendopeptidase activity	47	92	2,76E-05
GO:0008272	sulfate transport	12	13	2,91E-05
GO:0004383	guanylate cyclase activity	12	13	2,91E-05
GO:0016628	oxidoreductase activity, acting on the CH-CH group of donors, N	12	13	2,91E-05
GO:0051537	2 iron, 2 sulfur cluster binding	12	13	2,91E-05
GO:0009199	ribonucleoside triphosphate metabolism	30	51	3,47E-05
GO:0015250	water channel activity	10	10	3,71E-05
GO:0016854	racemase and epimerase activity	10	10	3,71E-05
GO:0019206	nucleoside kinase activity	10	10	3,71E-05
GO:0008299	isoprenoid biosynthesis	10	10	3,71E-05
GO:0009116	nucleoside metabolism	15	19	3,99E-05
GO:0000502	proteasome complex (sensu Eukaryota)	19	27	4,16E-05
GO:0016831	carboxy-lyase activity	17	23	4,42E-05
GO:0016126	sterol biosynthesis	17	23	4,42E-05
GO:0008076	voltage-gated potassium channel complex	25	40	4,45E-05
GO:0000270	peptidoglycan metabolism	13	15	4,75E-05
GO:0016709	oxidoreductase activity, acting on paired donors, with incorporat	13	15	4,75E-05
GO:0043450	alkene biosynthesis	13	15	4,75E-05
GO:0007046	ribosome biogenesis	46	91	5,22E-05
GO:0016779	nucleotidyltransferase activity	49	99	5,89E-05
GO:0046148	pigment biosynthesis	14	17	6,35E-05
GO:0005234	glutamate-gated ion channel activity	14	17	6,35E-05
GO:0031090	organelle membrane	158	411	7,53E-05
GO:0009205	purine ribonucleoside triphosphate metabolism	29	50	7,70E-05
GO:0006412	protein biosynthesis	186	496	7,74E-05
GO:0006979	response to oxidative stress	21	32	8,08E-05
GO:0031975	envelope	137	349	8,51E-05
GO:0031967	organelle envelope	137	349	8,51E-05
GO:0005231	excitatory extracellular ligand-gated ion channel activity	25	41	8,78E-05
GO:0005024	transforming growth factor beta receptor activity	11	12	8,78E-05
GO:0004675	transmembrane receptor protein serine/threonine kinase activity	11	12	8,78E-05
GO:0006570	tyrosine metabolism	11	12	8,78E-05
GO:0016323	basolateral plasma membrane	28	48	9,32E-05
GO:0015103	inorganic anion transporter activity	19	28	0,000101432

GO:0015662	ATPase activity, coupled to transmembrane movement of ions, p	35	65	0,00010483
GO:0016782	transferase activity, transferring sulfur-containing groups	27	46	0,000114445
GO:0000315	organellar large ribosomal subunit	14	18	0,000118427
GO:0004970	ionotropic glutamate receptor activity	14	18	0,000118427
GO:0005762	mitochondrial large ribosomal subunit	14	18	0,000118427
GO:0008374	O-acyltransferase activity	17	24	0,000118645
GO:0000163	protein phosphatase type 1 activity	9	9	0,000118691
GO:0006635	fatty acid beta-oxidation	9	9	0,000118691
GO:0016878	acid-thiol ligase activity	9	9	0,000118691
GO:0008239	dipeptidyl-peptidase activity	9	9	0,000118691
GO:0016806	dipeptidyl-peptidase and tripeptidyl-peptidase activity	9	9	0,000118691
GO:0016805	dipeptidase activity	9	9	0,000118691
GO:0044272	sulfur compound biosynthesis	16	22	0,000121548
GO:0004364	glutathione transferase activity	16	22	0,000121548
GO:0006695	cholesterol biosynthesis	15	20	0,000121548
GO:0004182	carboxypeptidase A activity	15	20	0,000121548
GO:0006364	rRNA processing	23	37	0,000122231
GO:0042364	water-soluble vitamin biosynthesis	12	14	0,000130842
GO:0016866	intramolecular transferase activity	12	14	0,000130842
GO:0016289	CoA hydrolase activity	12	14	0,000130842
GO:0019439	aromatic compound catabolism	12	14	0,000130842
GO:0005242	inward rectifier potassium channel activity	12	14	0,000130842
GO:0003678	DNA helicase activity	12	14	0,000130842
GO:0019370	leukotriene biosynthesis	12	14	0,000130842
GO:0015645	fatty-acid ligase activity	12	14	0,000130842
GO:0015674	di-, tri-valent inorganic cation transport	56	120	0,000138113
GO:0009142	nucleoside triphosphate biosynthesis	28	49	0,000162229
GO:0019395	fatty acid oxidation	13	16	0,000165528
GO:0016863	intramolecular oxidoreductase activity, transposing C=C bonds	13	16	0,000165528
GO:0031418	L-ascorbic acid binding	13	16	0,000165528
GO:0016791	phosphoric monoester hydrolase activity	88	210	0,000183813
GO:0006112	energy reserve metabolism	20	31	0,000194867
GO:0019829	cation-transporting ATPase activity	27	47	0,000199433
GO:0016775	phosphotransferase activity, nitrogenous group as acceptor	19	29	0,000224781
GO:0050381	unspecific monooxygenase activity	19	29	0,000224781
GO:0043285	biopolymer catabolism	71	163	0,000240194
GO:0046873	metal ion transporter activity	39	77	0,000247018
GO:0015298	solute:cation antiporter activity	10	11	0,000258098
GO:0015299	solute:hydrogen antiporter activity	10	11	0,000258098
GO:0006576	biogenic amine metabolism	28	50	0,000284067
GO:0009190	cyclic nucleotide biosynthesis	15	21	0,000334885
GO:0003724	RNA helicase activity	11	13	0,000376324
GO:0006100	tricarboxylic acid cycle intermediate metabolism	11	13	0,000376324
GO:0005003	ephrin receptor activity	11	13	0,000376324
GO:0006558	L-phenylalanine metabolism	8	8	0,000380804
GO:0015114	phosphate transporter activity	8	8	0,000380804
GO:0042559	pteridine and derivative biosynthesis	8	8	0,000380804
GO:0000178	exosome (RNase complex)	8	8	0,000380804
GO:0042578	phosphoric ester hydrolase activity	105	263	0,000394537
GO:0009206	purine ribonucleoside triphosphate biosynthesis	26	46	0,000432288
GO:0009145	purine nucleoside triphosphate biosynthesis	26	46	0,000432288
GO:0009201	ribonucleoside triphosphate biosynthesis	26	46	0,000432288
GO:0016072	rRNA metabolism	23	39	0,000445465
GO:0005247	voltage-gated chloride channel activity	12	15	0,000445465
GO:0006778	porphyrin metabolism	12	15	0,000445465
GO:0009110	vitamin biosynthesis	12	15	0,000445465
GO:0006691	leukotriene metabolism	12	15	0,000445465
GO:0042168	heme metabolism	12	15	0,000445465
GO:0004683	calmodulin regulated protein kinase activity	13	17	0,000484293
GO:0016758	transferase activity, transferring hexosyl groups	54	119	0,000544208
GO:0007028	cytoplasm organization and biogenesis	51	111	0,000569983
GO:0004867	serine-type endopeptidase inhibitor activity	42	87	0,00057616
GO:0005941	unlocalized protein complex	24	42	0,000663148
GO:0050661	NADP binding	9	10	0,000762454
GO:0006835	dicarboxylic acid transport	9	10	0,000762454
GO:0006783	heme biosynthesis	9	10	0,000762454
GO:0019363	pyridine nucleotide biosynthesis	9	10	0,000762454
GO:0004179	membrane alanyl aminopeptidase activity	9	10	0,000762454
GO:0000272	polysaccharide catabolism	9	10	0,000762454
GO:0006779	porphyrin biosynthesis	9	10	0,000762454
GO:0009074	aromatic amino acid family catabolism	9	10	0,000762454
GO:0044247	cellular polysaccharide catabolism	9	10	0,000762454
GO:0008484	sulfuric ester hydrolase activity	9	10	0,000762454
GO:0016284	alanine aminopeptidase activity	9	10	0,000762454
GO:0016408	C-acyltransferase activity	9	10	0,000762454
GO:0008276	protein methyltransferase activity	15	22	0,000823843
GO:0040029	regulation of gene expression, epigenetic	25	45	0,000918243
GO:0004289	subtilase activity	13	18	0,000964261
GO:0005201	extracellular matrix structural constituent	31	60	0,000995605

GO:0004467	long-chain-fatty-acid-CoA ligase activity	10	12	0,001026005
GO:0009220	pyrimidine ribonucleotide biosynthesis	10	12	0,001026005
GO:0015300	solute:solute antiporter activity	10	12	0,001026005
GO:0019751	polyol metabolism	10	12	0,001026005
GO:0009218	pyrimidine ribonucleotide metabolism	10	12	0,001026005
GO:0045103	intermediate filament-based process	10	12	0,001026005
GO:0016646	oxidoreductase activity, acting on the CH-NH group of donors, N	10	12	0,001026005
GO:0006071	glycerol metabolism	10	12	0,001026005
GO:0009225	nucleotide-sugar metabolism	10	12	0,001026005
GO:0008028	monocarboxylic acid transporter activity	10	12	0,001026005
GO:0005254	chloride channel activity	18	29	0,001136525
GO:0009112	nucleobase metabolism	11	14	0,001170162
GO:0016458	gene silencing	11	14	0,001170162
GO:0016706	oxidoreductase activity, acting on paired donors, with incorporat	11	14	0,001170162
GO:0009123	nucleoside monophosphate metabolism	11	14	0,001170162
GO:0006740	NADPH regeneration	7	7	0,001170162
GO:0004372	glycine hydroxymethyltransferase activity	7	7	0,001170162
GO:0004029	aldehyde dehydrogenase (NAD) activity	7	7	0,001170162
GO:0006032	chitin catabolism	7	7	0,001170162
GO:0045005	maintenance of fidelity during DNA-dependent DNA replication	7	7	0,001170162
GO:0046348	amino sugar catabolism	7	7	0,001170162
GO:0003906	DNA-(apurinic or apyrimidinic site) lyase activity	7	7	0,001170162
GO:0006046	N-acetylglucosamine catabolism	7	7	0,001170162
GO:0017153	sodium:dicarboxylate symporter activity	7	7	0,001170162
GO:0016165	lipoxygenase activity	7	7	0,001170162
GO:0046581	intercellular canaliculus	7	7	0,001170162
GO:0015377	cation:chloride symporter activity	7	7	0,001170162
GO:0016840	carbon-nitrogen lyase activity	7	7	0,001170162
GO:0006098	pentose-phosphate shunt	7	7	0,001170162
GO:0006559	L-phenylalanine catabolism	7	7	0,001170162
GO:0016405	CoA-ligase activity	7	7	0,001170162
GO:0009065	glutamine family amino acid catabolism	7	7	0,001170162
GO:0006043	glucosamine catabolism	7	7	0,001170162
GO:0016634	oxidoreductase activity, acting on the CH-CH group of donors, o	7	7	0,001170162
GO:0015359	amino acid permease activity	7	7	0,001170162
GO:0005677	chromatin silencing complex	7	7	0,001170162
GO:0042254	ribosome biogenesis and assembly	46	100	0,001181349
GO:0008033	tRNA processing	17	27	0,001297876
GO:0016811	hydrolase activity, acting on carbon-nitrogen (but not peptide) b	23	41	0,001346645
GO:0000160	two-component signal transduction system (phosphorelay)	20	34	0,001432435
GO:0006817	phosphate transport	27	51	0,00149769
GO:0008170	N-methyltransferase activity	19	32	0,001758983
GO:0016051	carbohydrate biosynthesis	30	59	0,001828372
GO:0016789	carboxylic ester hydrolase activity	49	110	0,002034639
GO:0006040	amino sugar metabolism	14	21	0,002059731
GO:0009166	nucleotide catabolism	14	21	0,002059731
GO:0003899	DNA-directed RNA polymerase activity	18	30	0,00212876
GO:0015116	sulfate transporter activity	8	9	0,00212876
GO:0017110	nucleoside-diphosphatase activity	8	9	0,00212876
GO:0045814	negative regulation of gene expression, epigenetic	8	9	0,00212876
GO:0004602	glutathione peroxidase activity	8	9	0,00212876
GO:0006342	chromatin silencing	8	9	0,00212876
GO:0006760	folic acid and derivative metabolism	8	9	0,00212876
GO:0042558	pteridine and derivative metabolism	8	9	0,00212876
GO:0016801	hydrolase activity, acting on ether bonds	8	9	0,00212876
GO:0031507	heterochromatin formation	8	9	0,00212876
GO:0019438	aromatic compound biosynthesis	8	9	0,00212876
GO:0004730	pseudouridylate synthase activity	8	9	0,00212876
GO:0005310	dicarboxylic acid transporter activity	8	9	0,00212876
GO:0009070	serine family amino acid biosynthesis	8	9	0,00212876
GO:0008271	sulfate porter activity	8	9	0,00212876
GO:0005840	ribosome	69	167	0,002225404
GO:0006090	pyruvate metabolism	13	19	0,002271818
GO:0045202	synapse	58	136	0,002472969
GO:0005977	glycogen metabolism	17	28	0,002557906
GO:0006073	glucan metabolism	17	28	0,002557906
GO:0016290	palmitoyl-CoA hydrolase activity	9	11	0,002700134
GO:0004016	adenylate cyclase activity	9	11	0,002700134
GO:0000302	response to reactive oxygen species	9	11	0,002700134
GO:0030551	cyclic nucleotide binding	12	17	0,002765287
GO:0006119	oxidative phosphorylation	24	45	0,002894252
GO:0004028	aldehyde dehydrogenase activity	11	15	0,002894252
GO:0006041	glucosamine metabolism	11	15	0,002894252
GO:0006044	N-acetylglucosamine metabolism	11	15	0,002894252
GO:0009147	pyrimidine nucleoside triphosphate metabolism	10	13	0,002894252
GO:0004889	nicotinic acetylcholine-activated cation-selective channel activity	10	13	0,002894252
GO:0006829	zinc ion transport	10	13	0,002894252
GO:0009161	ribonucleoside monophosphate metabolism	10	13	0,002894252
GO:0009124	nucleoside monophosphate biosynthesis	10	13	0,002894252

GO:0015992	proton transport	29	58	0,003361315
GO:0016668	oxidoreductase activity, acting on sulfur group of donors, NAD o	6	6	0,003681371
GO:0006081	aldehyde metabolism	6	6	0,003681371
GO:0006298	mismatch repair	6	6	0,003681371
GO:0016624	oxidoreductase activity, acting on the aldehyde or oxo group of	6	6	0,003681371
GO:0006544	glycine metabolism	6	6	0,003681371
GO:0016803	ether hydrolase activity	6	6	0,003681371
GO:0006012	galactose metabolism	6	6	0,003681371
GO:0004303	estradiol 17-beta-dehydrogenase activity	6	6	0,003681371
GO:0004568	chitinase activity	6	6	0,003681371
GO:0004463	leukotriene-A4 hydrolase activity	6	6	0,003681371
GO:0006560	proline metabolism	6	6	0,003681371
GO:0016857	racemase and epimerase activity, acting on carbohydrates and (	6	6	0,003681371
GO:0016406	carnitine O-acyltransferase activity	6	6	0,003681371
GO:0004274	dipeptidyl-peptidase IV activity	6	6	0,003681371
GO:0005283	sodium:amino acid symporter activity	6	6	0,003681371
GO:0046915	transition metal ion transporter activity	14	22	0,00429871
GO:0016638	oxidoreductase activity, acting on the CH-NH2 group of donors	14	22	0,00429871
GO:0005625	soluble fraction	24	46	0,00456798
GO:0007169	transmembrane receptor protein tyrosine kinase signaling pathw	45	102	0,004573786
GO:0015399	primary active transporter activity	69	171	0,005022573
GO:0046364	monosaccharide biosynthesis	13	20	0,005038786
GO:0019319	hexose biosynthesis	13	20	0,005038786
GO:0046165	alcohol biosynthesis	13	20	0,005038786
GO:0008146	sulfotransferase activity	21	39	0,005730205
GO:0009187	cyclic nucleotide metabolism	16	27	0,005797636
GO:0015085	calcium ion transporter activity	7	8	0,006051276
GO:0004685	calcium- and calmodulin-dependent protein kinase activity	7	8	0,006051276
GO:0006563	L-serine metabolism	7	8	0,006051276
GO:0019104	DNA N-glycosylase activity	7	8	0,006051276
GO:0006030	chitin metabolism	7	8	0,006051276
GO:0005388	calcium-transporting ATPase activity	7	8	0,006051276
GO:0006525	arginine metabolism	7	8	0,006051276
GO:0006857	oligopeptide transport	7	8	0,006051276
GO:0019674	NAD metabolism	7	8	0,006051276
GO:0000051	urea cycle intermediate metabolism	7	8	0,006051276
GO:0006013	mannose metabolism	7	8	0,006051276
GO:0006354	RNA elongation	7	8	0,006051276
GO:0009084	glutamine family amino acid biosynthesis	7	8	0,006051276
GO:0001518	voltage-gated sodium channel complex	7	8	0,006051276
GO:0006818	hydrogen transport	31	65	0,00632875
GO:0006643	membrane lipid metabolism	38	84	0,00655122
GO:0043037	translation	63	155	0,006712875
GO:0005044	scavenger receptor activity	15	25	0,006937127
GO:0004673	protein histidine kinase activity	15	25	0,006937127
GO:0042054	histone methyltransferase activity	10	14	0,006937127
GO:0004293	tissue kallikrein activity	10	14	0,006937127
GO:0016208	AMP binding	10	14	0,006937127
GO:0004536	deoxyribonuclease activity	10	14	0,006937127
GO:0042219	amino acid derivative catabolism	10	14	0,006937127
GO:0046039	GTP metabolism	8	10	0,006940072
GO:0009208	pyrimidine ribonucleoside triphosphate metabolism	8	10	0,006940072
GO:0009262	deoxyribonucleotide metabolism	8	10	0,006940072
GO:0004003	ATP-dependent DNA helicase activity	8	10	0,006940072
GO:0046036	CTP metabolism	8	10	0,006940072
GO:0046051	UTP metabolism	8	10	0,006940072
GO:0009132	nucleoside diphosphate metabolism	8	10	0,006940072
GO:0006241	CTP biosynthesis	8	10	0,006940072
GO:0006739	NADP metabolism	8	10	0,006940072
GO:0004550	nucleoside diphosphate kinase activity	8	10	0,006940072
GO:0006228	UTP biosynthesis	8	10	0,006940072
GO:0016868	intramolecular transferase activity, phosphotransferases	8	10	0,006940072
GO:0005355	glucose transporter activity	8	10	0,006940072
GO:0009209	pyrimidine ribonucleoside triphosphate biosynthesis	8	10	0,006940072
GO:0006183	GTP biosynthesis	8	10	0,006940072
GO:0009066	aspartate family amino acid metabolism	9	12	0,007093674
GO:0015491	cation:cation antiporter activity	9	12	0,007093674
GO:0042402	biogenic amine catabolism	9	12	0,007093674
GO:0009156	ribonucleoside monophosphate biosynthesis	9	12	0,007093674
GO:0016874	ligase activity	134	371	0,007447046
GO:0042445	hormone metabolism	27	55	0,007447046
GO:0030163	protein catabolism	62	153	0,007832207
GO:0006310	DNA recombination	19	35	0,008651819
GO:0005761	mitochondrial ribosome	21	40	0,00873511
GO:0046034	ATP metabolism	21	40	0,00873511
GO:0000313	organellar ribosome	21	40	0,00873511

eukaryota

GO:0005622	intracellular	1820	6664	0

GO:0043226	organelle	1587	5789	0
GO:0043229	intracellular organelle	1586	5785	0
GO:0043227	membrane-bound organelle	1419	5097	0
GO:0043231	intracellular membrane-bound organelle	1417	5092	0
GO:0005634	nucleus	1054	3267	0
GO:0046914	transition metal ion binding	644	1791	0
GO:0008270	zinc ion binding	619	1416	0
GO:0004888	transmembrane receptor activity	23	2007	0
GO:0043169	cation binding	799	2589	3,45E-85
GO:0006351	transcription, DNA-dependent	559	1588	5,29E-85
GO:0006355	regulation of transcription, DNA-dependent	552	1563	1,23E-84
GO:0006350	transcription	580	1677	2,88E-84
GO:0045449	regulation of transcription	566	1622	3,62E-84
GO:0019219	regulation of nucleobase, nucleoside, nucleotide and nucleic acid	569	1638	1,34E-83
GO:0006512	ubiquitin cycle	222	423	4,53E-81
GO:0019222	regulation of metabolism	597	1812	1,87E-75
GO:0031323	regulation of cellular metabolism	582	1750	1,87E-75
GO:0003676	nucleic acid binding	738	2432	1,68E-73
GO:0046872	metal ion binding	815	2795	1,13E-71
GO:0043167	ion binding	815	2795	1,13E-71
GO:0050875	cellular physiological process	1860	8198	7,75E-71
GO:0006139	nucleobase, nucleoside, nucleotide and nucleic acid metabolism	736	2461	1,12E-69
GO:0051244	regulation of cellular physiological process	705	2352	8,03E-67
GO:0050791	regulation of physiological process	715	2444	3,23E-62
GO:0050794	regulation of cellular process	722	2477	4,33E-62
GO:0003677	DNA binding	535	1670	2,95E-61
GO:0016881	acid-amino acid ligase activity	133	224	1,13E-60
GO:0050789	regulation of biological process	753	2683	1,95E-56
GO:0004842	ubiquitin-protein ligase activity	123	209	3,65E-55
GO:0016879	ligase activity, forming carbon-nitrogen bonds	133	253	2,72E-48
GO:0044238	primary metabolism	1240	5312	2,04E-43
GO:0030695	GTPase regulator activity	122	245	1,19E-39
GO:0044237	cellular metabolism	1268	5566	2,65E-38
GO:0005654	nucleoplasm	191	479	4,12E-38
GO:0005667	transcription factor complex	161	384	4,17E-36
GO:0031981	nuclear lumen	212	573	5,93E-35
GO:0046907	intracellular transport	203	544	3,37E-34
GO:0000151	ubiquitin ligase complex	91	170	5,82E-34
GO:0051649	establishment of cellular localization	203	548	1,52E-33
GO:0051641	cellular localization	204	553	2,42E-33
GO:0015031	protein transport	206	564	8,79E-33
GO:0045184	establishment of protein localization	210	583	3,50E-32
GO:0008152	metabolism	1296	5906	3,47E-30
GO:0043233	organelle lumen	222	645	6,31E-30
GO:0031974	membrane-enclosed lumen	222	645	6,31E-30
GO:0008104	protein localization	212	607	8,62E-30
GO:0016567	protein ubiquitination	82	161	1,01E-27
GO:0043283	biopolymer metabolism	538	2067	1,61E-27
GO:0016874	ligase activity	145	371	2,42E-27
GO:0016043	cell organization and biogenesis	416	1503	3,20E-27
GO:0005096	GTPase activator activity	68	123	6,72E-27
GO:0003700	transcription factor activity	242	761	1,38E-25
GO:0030117	membrane coat	32	39	3,10E-24
GO:0048475	coated membrane	32	39	3,10E-24
GO:0030120	vesicle coat	31	37	3,24E-24
GO:0008047	enzyme activator activity	79	164	7,64E-24
GO:0030118	clathrin coat	28	33	3,15E-22
GO:0006464	protein modification	347	1256	3,15E-22
GO:0004843	ubiquitin-specific protease activity	37	53	8,15E-22
GO:0030125	clathrin vesicle coat	27	32	3,10E-21
GO:0016192	vesicle-mediated transport	115	297	4,15E-21
GO:0043412	biopolymer modification	352	1295	4,61E-21
GO:0004221	ubiquitin thiolesterase activity	34	48	1,72E-20
GO:0008565	protein transporter activity	59	116	7,94E-20
GO:0006511	ubiquitin-dependent protein catabolism	53	100	2,79E-19
GO:0019941	modification-dependent protein catabolism	54	103	3,04E-19
GO:0005083	small GTPase regulator activity	69	149	3,90E-19
GO:0030135	coated vesicle	50	93	1,08E-18
GO:0008639	small protein conjugating enzyme activity	26	33	2,02E-18
GO:0005085	guanyl-nucleotide exchange factor activity	52	100	3,06E-18
GO:0042578	phosphoric ester hydrolase activity	101	263	3,91E-18
GO:0004840	ubiquitin conjugating enzyme activity	24	30	2,29E-17
GO:0005856	cytoskeleton	208	704	6,02E-17
GO:0051301	cell division	73	171	6,56E-17
GO:0015629	actin cytoskeleton	69	161	4,05E-16
GO:0003702	RNA polymerase II transcription factor activity	35	58	4,18E-16
GO:0007049	cell cycle	165	536	1,76E-15
GO:0030136	clathrin-coated vesicle	44	85	2,89E-15
GO:0007010	cytoskeleton organization and biogenesis	126	381	7,08E-15

GO:0016311	dephosphorylation	50	105	1,13E-14
GO:0005623	cell	2148	11358	2,26E-14
GO:0006470	protein amino acid dephosphorylation	49	103	2,38E-14
GO:0009987	cellular process	2041	10723	4,54E-14
GO:0006366	transcription from RNA polymerase II promoter	104	301	5,44E-14
GO:0006886	intracellular protein transport	122	373	6,33E-14
GO:0008642	ubiquitin-like activating enzyme activity	22	30	7,07E-14
GO:0048193	Golgi vesicle transport	35	63	7,14E-14
GO:0043228	non-membrane-bound organelle	309	1211	1,07E-13
GO:0043232	intracellular non-membrane-bound organelle	309	1211	1,07E-13
GO:0006996	organelle organization and biogenesis	224	818	1,47E-13
GO:0008641	small protein activating enzyme activity	23	33	2,13E-13
GO:0015630	microtubule cytoskeleton	84	231	4,77E-13
GO:0016790	thiolester hydrolase activity	35	66	1,05E-12
GO:0044257	cellular protein catabolism	54	127	2,19E-12
GO:0051603	proteolysis during cellular protein catabolism	54	127	2,19E-12
GO:0004721	phosphoprotein phosphatase activity	58	141	2,57E-12
GO:0016788	hydrolase activity, acting on ester bonds	162	560	3,77E-12
GO:0006888	ER to Golgi transport	28	48	4,20E-12
GO:0051234	establishment of localization	566	2534	6,81E-12
GO:0005643	nuclear pore	21	31	1,18E-11
GO:0046930	pore complex	21	31	1,18E-11
GO:0051179	localization	568	2554	1,44E-11
GO:0000278	mitotic cell cycle	62	159	1,44E-11
GO:0006810	transport	523	2323	1,57E-11
GO:0000087	M phase of mitotic cell cycle	48	112	3,19E-11
GO:0005874	microtubule	59	151	4,47E-11
GO:0007017	microtubule-based process	56	142	9,36E-11
GO:0007067	mitosis	47	111	1,07E-10
GO:0016791	phosphoric monoester hydrolase activity	74	210	1,48E-10
GO:0030705	cytoskeleton-dependent intracellular transport	38	82	1,50E-10
GO:0051020	GTPase binding	27	49	1,57E-10
GO:0007018	microtubule-based movement	36	76	1,82E-10
GO:0000279	M phase	59	155	2,26E-10
GO:0006357	regulation of transcription from RNA polymerase II promoter	86	260	3,44E-10
GO:0004428	inositol or phosphatidylinositol kinase activity	19	29	5,73E-10
GO:0030163	protein catabolism	57	153	1,80E-09
GO:0016564	transcriptional repressor activity	47	117	1,89E-09
GO:0019899	enzyme binding	39	90	2,35E-09
GO:0016071	mRNA metabolism	65	186	4,61E-09
GO:0005905	coated pit	21	36	4,87E-09
GO:0016538	cyclin-dependent protein kinase regulator activity	16	19	5,73E-09
GO:0006397	mRNA processing	59	165	8,89E-09
GO:0005794	Golgi apparatus	121	423	1,07E-08
GO:0005515	protein binding	672	3209	1,99E-08
GO:0045045	secretory pathway	56	156	1,99E-08
GO:0005737	cytoplasm	649	3087	2,10E-08
GO:0000059	protein import into nucleus, docking	13	14	2,19E-08
GO:0000775	chromosome, pericentric region	21	38	3,34E-08
GO:0008234	cysteine-type peptidase activity	51	139	3,74E-08
GO:0043170	macromolecule metabolism	686	3298	4,38E-08
GO:0043285	biopolymer catabolism	57	163	5,56E-08
GO:0046903	secretion	63	187	6,48E-08
GO:0008081	phosphoric diester hydrolase activity	27	57	7,64E-08
GO:0051726	regulation of cell cycle	85	279	1,01E-07
GO:0000074	regulation of progression through cell cycle	85	279	1,01E-07
GO:0045934	negative regulation of nucleobase, nucleoside, nucleotide and nu	56	162	1,35E-07
GO:0016481	negative regulation of transcription	54	155	1,70E-07
GO:0006913	nucleocytoplasmic transport	35	85	1,73E-07
GO:0044267	cellular protein metabolism	525	2469	2,41E-07
GO:0006650	glycerophospholipid metabolism	18	27	2,43E-07
GO:0005795	Golgi stack	94	322	2,46E-07
GO:0003779	actin binding	64	199	5,48E-07
GO:0004629	phospholipase C activity	11	12	5,59E-07
GO:0001727	lipid kinase activity	16	23	5,88E-07
GO:0005802	Golgi trans face	16	23	5,88E-07
GO:0031252	leading edge	25	54	6,08E-07
GO:0019538	protein metabolism	525	2486	6,51E-07
GO:0044260	cellular macromolecule metabolism	527	2500	7,88E-07
GO:0004725	protein tyrosine phosphatase activity	34	85	8,16E-07
GO:0031267	small GTPase binding	21	42	8,16E-07
GO:0005884	actin filament	14	19	1,30E-06
GO:0006396	RNA processing	75	249	1,37E-06
GO:0031982	vesicle	58	180	2,13E-06
GO:0008134	transcription factor binding	47	137	2,82E-06
GO:0007242	intracellular signaling cascade	175	714	2,91E-06
GO:0005798	Golgi-associated vesicle	16	25	3,20E-06
GO:0051169	nuclear transport	30	74	3,24E-06
GO:0051243	negative regulation of cellular physiological process	117	441	3,32E-06

GO:0005875	microtubule associated complex	35	92	3,32E-06
GO:0030027	lamellipodium	20	41	3,47E-06
GO:0003712	transcription cofactor activity	41	115	4,03E-06
GO:0031410	cytoplasmic vesicle	56	175	4,63E-06
GO:0031988	membrane-bound vesicle	55	171	4,72E-06
GO:0030384	phosphoinositide metabolism	13	18	5,14E-06
GO:0006606	protein import into nucleus	21	45	5,97E-06
GO:0051170	nuclear import	21	45	5,97E-06
GO:0043118	negative regulation of physiological process	120	460	6,53E-06
GO:0004437	inositol or phosphatidylinositol phosphatase activity	16	26	6,53E-06
GO:0009892	negative regulation of metabolism	66	219	7,51E-06
GO:0016563	transcriptional activator activity	44	129	8,19E-06
GO:0005525	GTP binding	81	285	8,94E-06
GO:0031324	negative regulation of cellular metabolism	57	182	8,94E-06
GO:0005452	inorganic anion exchanger activity	8	8	8,94E-06
GO:0008092	cytoskeletal protein binding	78	272	9,15E-06
GO:0017038	protein import	22	49	9,15E-06
GO:0016023	cytoplasmic membrane-bound vesicle	53	166	9,90E-06
GO:0006323	DNA packaging	59	191	1,02E-05
GO:0006259	DNA metabolism	117	452	1,41E-05
GO:0051258	protein polymerization	16	31	1,55E-05
GO:0030133	transport vesicle	12	17	1,98E-05
GO:0016568	chromatin modification	36	101	2,06E-05
GO:0003743	translation initiation factor activity	29	75	2,12E-05
GO:0019001	guanyl nucleotide binding	81	291	2,45E-05
GO:0006325	establishment and/or maintenance of chromatin architecture	56	183	2,84E-05
GO:0012505	endomembrane system	60	200	2,85E-05
GO:0045892	negative regulation of transcription, DNA-dependent	39	115	4,22E-05
GO:0008536	Ran GTPase binding	7	7	4,77E-05
GO:0030036	actin cytoskeleton organization and biogenesis	39	116	5,61E-05
GO:0004434	inositol or phosphatidylinositol phosphodiesterase activity	8	9	6,15E-05
GO:0004435	phosphoinositide phospholipase C activity	8	9	6,15E-05
GO:0006914	autophagy	11	16	7,62E-05
GO:0003714	transcription corepressor activity	19	43	7,90E-05
GO:0005681	spliceosome complex	32	91	0,000115203
GO:0045941	positive regulation of transcription	53	177	0,000117877
GO:0044265	cellular macromolecule catabolism	61	212	0,000126307
GO:0045935	positive regulation of nucleobase, nucleoside, nucleotide and nu	53	178	0,000143669
GO:0031975	envelope	91	349	0,000151429
GO:0031967	organelle envelope	91	349	0,000151429
GO:0048489	synaptic vesicle transport	11	17	0,000171479
GO:0043087	regulation of GTPase activity	12	20	0,000210772
GO:0000375	RNA splicing, via transesterification reactions	37	113	0,000213057
GO:0000377	RNA splicing, via transesterification reactions with bulged adeno	37	113	0,000213057
GO:0000398	nuclear mRNA splicing, via spliceosome	37	113	0,000213057
GO:0016307	phosphatidylinositol phosphate kinase activity	8	10	0,000234651
GO:0015301	anion:anion antiporter activity	8	10	0,000234651
GO:0015380	anion exchanger activity	8	10	0,000234651
GO:0030130	clathrin coat of trans-Golgi network vesicle	8	10	0,000234651
GO:0015106	bicarbonate transporter activity	8	10	0,000234651
GO:0000159	protein phosphatase type 2A complex	6	6	0,000241016
GO:0004835	tubulin-tyrosine ligase activity	6	6	0,000241016
GO:0046839	phospholipid dephosphorylation	6	6	0,000241016
GO:0004571	mannosyl-oligosaccharide 1,2-alpha-mannosidase activity	6	6	0,000241016
GO:0006497	protein amino acid lipidation	15	32	0,000242494
GO:0003887	DNA-directed DNA polymerase activity	15	32	0,000242494
GO:0030029	actin filament-based process	39	122	0,000253645
GO:0005694	chromosome	72	266	0,000265364
GO:0005938	cell cortex	14	29	0,000266269
GO:0005669	transcription factor TFIID complex	10	15	0,000266269
GO:0016308	1-phosphatidylinositol-4-phosphate 5-kinase activity	7	8	0,00027292
GO:0005100	Rho GTPase activator activity	7	8	0,00027292
GO:0045893	positive regulation of transcription, DNA-dependent	43	139	0,000274023
GO:0003777	microtubule motor activity	23	60	0,000288641
GO:0016070	RNA metabolism	84	322	0,000291137
GO:0006413	translational initiation	17	39	0,000300218
GO:0005783	endoplasmic reticulum	119	489	0,000313857
GO:0008380	RNA splicing	38	119	0,000321803
GO:0051336	regulation of hydrolase activity	19	46	0,000322084
GO:0004114	3',5'-cyclic-nucleotide phosphodiesterase activity	13	24	0,000377825
GO:0003723	RNA binding	101	405	0,000414263
GO:0046474	glycerophospholipid biosynthesis	9	13	0,000417802
GO:0042158	lipoprotein biosynthesis	15	33	0,000420791
GO:0030286	dynein complex	14	28	0,000595609
GO:0005543	phospholipid binding	18	44	0,000634658
GO:0008320	protein carrier activity	8	11	0,000645093
GO:0008289	lipid binding	49	170	0,000782669
GO:0031090	organelle membrane	101	411	0,000796691
GO:0009057	macromolecule catabolism	64	238	0,000901311

GO:0003924	GTPase activity	35	111	0,000916758
GO:0004197	cysteine-type endopeptidase activity	34	107	0,000936592
GO:0006904	vesicle docking during exocytosis	9	14	0,000936592
GO:0048278	vesicle docking	9	14	0,000936592
GO:0030140	trans-Golgi network transport vesicle	9	14	0,000936592
GO:0008180	signalosome complex	7	9	0,000942175
GO:0005635	nuclear envelope	26	75	0,00097767
GO:0004112	cyclic-nucleotide phosphodiesterase activity	13	26	0,001035594
GO:0048523	negative regulation of cellular process	125	531	0,00104905
GO:0006352	transcription initiation	12	23	0,001110806
GO:0008601	protein phosphatase type 2A regulator activity	6	7	0,001221389
GO:0015924	mannosyl-oligosaccharide mannosidase activity	6	7	0,001221389
GO:0042393	histone binding	6	7	0,001221389
GO:0006897	endocytosis	41	138	0,001275475
GO:0042175	nuclear envelope-endoplasmic reticulum network	20	53	0,001282564
GO:0000122	negative regulation of transcription from RNA polymerase II pro	28	84	0,001312136
GO:0006644	phospholipid metabolism	21	57	0,001412371
GO:0035004	phosphoinositide 3-kinase activity	8	12	0,001517813
GO:0015108	chloride transporter activity	8	12	0,001517813
GO:0006839	mitochondrial transport	8	12	0,001517813
GO:0008287	protein serine/threonine phosphatase complex	9	15	0,001860896
GO:0045944	positive regulation of transcription from RNA polymerase II pron	35	115	0,002190809
GO:0016459	myosin	16	40	0,002395696
GO:0016747	transferase activity, transferring groups other than amino-acyl g	40	137	0,002424459
GO:0030258	lipid modification	7	10	0,002474431
GO:0005200	structural constituent of cytoskeleton	29	91	0,002786151
GO:0005478	intracellular transporter activity	8	13	0,003190858
GO:0004468	lysine N-acetyltransferase activity	8	13	0,003190858
GO:0004402	histone acetyltransferase activity	8	13	0,003190858
GO:0043543	protein amino acid acylation	8	13	0,003190858
GO:0042157	lipoprotein metabolism	16	41	0,003568731
GO:0000785	chromatin	42	148	0,00358976
GO:0006643	membrane lipid metabolism	27	84	0,003713751
GO:0016251	general RNA polymerase II transcription factor activity	6	8	0,00379627
GO:0006302	double-strand break repair	6	8	0,00379627
GO:0007264	small GTPase mediated signal transduction	56	212	0,003940142
GO:0016591	DNA-directed RNA polymerase II, holoenzyme	17	45	0,004002558
GO:0048519	negative regulation of biological process	131	581	0,00531556
GO:0005942	phosphoinositide 3-kinase complex	7	11	0,005358162
GO:0016303	phosphatidylinositol 3-kinase activity	7	11	0,005358162
GO:0006506	GPI anchor biosynthesis	5	6	0,005358162
GO:0016925	protein sumoylation	5	6	0,005358162
GO:0046489	phosphoinositide biosynthesis	5	6	0,005358162
GO:0008135	translation factor activity, nucleic acid binding	35	120	0,005741068
GO:0000776	kinetochore	9	17	0,005817349
GO:0000075	cell cycle checkpoint	9	17	0,005817349
GO:0003705	RNA polymerase II transcription factor activity, enhancer bindin	8	14	0,00588154
GO:0046934	phosphatidylinositol-4,5-bisphosphate 3-kinase activity	4	4	0,005946567
GO:0005789	endoplasmic reticulum membrane	18	50	0,006010854
GO:0016779	nucleotidyltransferase activity	30	99	0,006322049
GO:0003713	transcription coactivator activity	20	58	0,006447575
GO:0004620	phospholipase activity	15	39	0,006583104
GO:0008415	acyltransferase activity	38	135	0,007794197
GO:0003682	chromatin binding	21	63	0,008625806
GO:0004194	pepsin A activity	6	9	0,008947528
GO:0019904	protein domain specific binding	12	29	0,009198002
GO:0016746	transferase activity, transferring acyl groups	40	145	0,009272999
GO:0000910	cytokinesis	9	18	0,00944697

**metazoa**				
GO:0016020	membrane	1768	6163	0
GO:0031224	intrinsic to membrane	1524	4932	0
GO:0016021	integral to membrane	1523	4930	0
GO:0007154	cell communication	1234	3201	0
GO:0007165	signal transduction	1211	3059	0
GO:0004872	receptor activity	1143	2793	0
GO:0007166	cell surface receptor linked signal transduction	1061	2253	0
GO:0004888	transmembrane receptor activity	926	2007	0
GO:0007186	G-protein coupled receptor protein signaling pathway	906	1763	0
GO:0004930	G-protein coupled receptor activity	870	1693	0
GO:0001584	rhodopsin-like receptor activity	808	1478	0
GO:0050877	neurophysiological process	692	1581	0
GO:0007600	sensory perception	661	1438	0
GO:0007606	sensory perception of chemical stimulus	600	1231	0
GO:0007608	sensory perception of smell	597	1182	0
GO:0004984	olfactory receptor activity	594	1165	0
GO:0050874	organismal physiological process	871	2571	2,24E-73
GO:0001653	peptide receptor activity	95	111	1,69E-62
GO:0008528	peptide receptor activity, G-protein coupled	95	111	1,69E-62

GO:0005623	cell	2681	11358	1,35E-47
GO:0042277	peptide binding	96	138	3,20E-44
GO:0001608	nucleotide receptor activity, G-protein coupled	50	53	7,54E-38
GO:0001614	purinergic nucleotide receptor activity	50	53	7,54E-38
GO:0016502	nucleotide receptor activity	50	53	7,54E-38
GO:0045028	purinergic nucleotide receptor activity, G-protein coupled	50	53	7,54E-38
GO:0042923	neuropeptide binding	48	52	4,38E-35
GO:0008188	neuropeptide receptor activity	48	52	4,38E-35
GO:0009653	morphogenesis	297	788	8,38E-33
GO:0009987	cellular process	2482	10723	1,15E-29
GO:0009887	organ morphogenesis	182	429	2,84E-28
GO:0004983	neuropeptide Y receptor activity	38	41	8,92E-28
GO:0003707	steroid hormone receptor activity	41	47	2,66E-27
GO:0004879	ligand-dependent nuclear receptor activity	41	47	2,66E-27
GO:0030154	cell differentiation	269	747	2,70E-25
GO:0003700	transcription factor activity	268	761	3,12E-23
GO:0048513	organ development	287	845	9,60E-22
GO:0050794	regulation of cellular process	688	2477	4,41E-21
GO:0030594	neurotransmitter receptor activity	59	97	7,34E-21
GO:0042165	neurotransmitter binding	59	97	7,34E-21
GO:0050789	regulation of biological process	730	2683	8,67E-20
GO:0048519	negative regulation of biological process	205	581	1,00E-17
GO:0051244	regulation of cellular physiological process	639	2352	8,93E-17
GO:0050791	regulation of physiological process	659	2444	1,66E-16
GO:0016526	G-protein coupled receptor activity, unknown ligand	27	33	5,46E-16
GO:0008227	amine receptor activity	35	50	5,72E-16
GO:0048523	negative regulation of cellular process	186	531	1,26E-15
GO:0048731	system development	159	444	2,14E-14
GO:0030246	carbohydrate binding	101	247	6,41E-14
GO:0007389	pattern specification	77	172	1,36E-13
GO:0019222	regulation of metabolism	497	1812	1,90E-13
GO:0048514	blood vessel morphogenesis	53	105	1,62E-12
GO:0043119	positive regulation of physiological process	146	416	2,93E-12
GO:0048518	positive regulation of biological process	171	511	5,32E-12
GO:0007399	nervous system development	142	404	5,36E-12
GO:0019219	regulation of nucleobase, nucleoside, nucleotide and nucleic acic	449	1638	5,41E-12
GO:0045449	regulation of transcription	445	1622	6,00E-12
GO:0001525	angiogenesis	48	93	6,17E-12
GO:0006350	transcription	457	1677	8,89E-12
GO:0031323	regulation of cellular metabolism	474	1750	9,09E-12
GO:0001944	vasculature development	58	123	1,07E-11
GO:0016055	Wnt receptor signaling pathway	49	97	1,23E-11
GO:0005515	protein binding	803	3209	1,75E-11
GO:0001568	blood vessel development	57	121	1,75E-11
GO:0045941	positive regulation of transcription	75	177	1,78E-11
GO:0006351	transcription, DNA-dependent	434	1588	2,15E-11
GO:0045935	positive regulation of nucleobase, nucleoside, nucleotide and nu	75	178	2,64E-11
GO:0007223	frizzled-2 signaling pathway	19	20	3,35E-11
GO:0006355	regulation of transcription, DNA-dependent	427	1563	3,55E-11
GO:0005529	sugar binding	75	179	3,87E-11
GO:0048522	positive regulation of cellular process	147	434	7,03E-11
GO:0008083	growth factor activity	62	141	1,65E-10
GO:0005667	transcription factor complex	132	384	2,47E-10
GO:0009968	negative regulation of signal transduction	35	63	3,03E-10
GO:0051242	positive regulation of cellular physiological process	136	400	3,25E-10
GO:0045893	positive regulation of transcription, DNA-dependent	60	139	1,14E-09
GO:0043118	negative regulation of physiological process	150	460	1,58E-09
GO:0051243	negative regulation of cellular physiological process	145	441	1,58E-09
GO:0019956	chemokine binding	20	24	1,59E-09
GO:0001637	G-protein chemoattractant receptor activity	20	24	1,59E-09
GO:0004950	chemokine receptor activity	20	24	1,59E-09
GO:0000902	cellular morphogenesis	109	308	1,66E-09
GO:0004935	adrenoceptor activity	16	17	2,81E-09
GO:0006357	regulation of transcription from RNA polymerase II promoter	95	260	2,91E-09
GO:0005654	nucleoplasm	154	479	3,15E-09
GO:0045165	cell fate commitment	36	69	3,22E-09
GO:0006366	transcription from RNA polymerase II promoter	106	301	4,35E-09
GO:0009790	embryonic development	95	263	6,56E-09
GO:0045944	positive regulation of transcription from RNA polymerase II pron	51	115	7,49E-09
GO:0006486	protein amino acid glycosylation	35	68	1,08E-08
GO:0008373	sialyltransferase activity	17	20	2,26E-08
GO:0043413	biopolymer glycosylation	35	70	3,82E-08
GO:0009893	positive regulation of metabolism	83	228	5,30E-08
GO:0031325	positive regulation of cellular metabolism	80	219	8,41E-08
GO:0007411	axon guidance	28	52	1,17E-07
GO:0040008	regulation of growth	41	90	1,26E-07
GO:0040011	locomotion	79	217	1,27E-07
GO:0016477	cell migration	68	179	1,47E-07
GO:0048699	neurogenesis	75	204	1,74E-07

GO:0009892	negative regulation of metabolism	79	219	2,16E-07
GO:0006928	cell motility	77	212	2,21E-07
GO:0051674	localization of cell	77	212	2,21E-07
GO:0008283	cell proliferation	97	285	2,41E-07
GO:0009100	glycoprotein metabolism	38	83	3,59E-07
GO:0009101	glycoprotein biosynthesis	35	74	3,70E-07
GO:0031981	nuclear lumen	170	573	5,05E-07
GO:0030182	neuron differentiation	67	181	7,02E-07
GO:0042127	regulation of cell proliferation	67	181	7,02E-07
GO:0009966	regulation of signal transduction	54	137	9,02E-07
GO:0003677	DNA binding	426	1670	9,49E-07
GO:0004993	serotonin receptor activity	13	15	1,20E-06
GO:0004926	non-G-protein coupled 7TM receptor activity	10	10	2,01E-06
GO:0048468	cell development	79	229	2,45E-06
GO:0030173	integral to Golgi membrane	16	22	2,89E-06
GO:0031228	intrinsic to Golgi membrane	16	22	2,89E-06
GO:0043233	organelle lumen	184	645	3,55E-06
GO:0031974	membrane-enclosed lumen	184	645	3,55E-06
GO:0045125	bioactive lipid receptor activity	11	12	3,82E-06
GO:0016757	transferase activity, transferring glycosyl groups	71	202	4,08E-06
GO:0000904	cellular morphogenesis during differentiation	52	135	4,23E-06
GO:0008361	regulation of cell size	34	77	6,95E-06
GO:0018298	protein-chromophore linkage	9	9	9,11E-06
GO:0007218	neuropeptide signaling pathway	33	75	1,16E-05
GO:0019955	cytokine binding	30	66	1,35E-05
GO:0016493	C-C chemokine receptor activity	13	17	1,56E-05
GO:0019957	C-C chemokine binding	13	17	1,56E-05
GO:0001619	lysosphingolipid and lysophosphatidic acid receptor activity	10	11	1,60E-05
GO:0007507	heart development	37	89	1,87E-05
GO:0016049	cell growth	32	73	1,89E-05
GO:0007409	axonogenesis	39	97	3,13E-05
GO:0009952	anterior/posterior pattern formation	26	56	3,99E-05
GO:0019958	C-X-C chemokine binding	8	8	4,02E-05
GO:0016494	C-X-C chemokine receptor activity	8	8	4,02E-05
GO:0005104	fibroblast growth factor receptor binding	15	25	4,06E-05
GO:0030509	BMP signaling pathway	15	23	5,09E-05
GO:0001649	osteoblast differentiation	12	16	5,26E-05
GO:0007178	transmembrane receptor protein serine/threonine kinase signali	29	66	5,38E-05
GO:0005158	insulin receptor binding	9	10	6,29E-05
GO:0009888	tissue development	63	185	6,95E-05
GO:0048666	neuron development	52	145	7,08E-05
GO:0031175	neurite morphogenesis	44	117	7,56E-05
GO:0048667	neuron morphogenesis during differentiation	44	117	7,56E-05
GO:0007417	central nervous system development	39	100	8,07E-05
GO:0001633	secretin-like receptor activity	13	19	9,78E-05
GO:0009792	embryonic development (sensu Metazoa)	43	115	0,000116907
GO:0007155	cell adhesion	134	469	0,000129709
GO:0009880	embryonic pattern specification	17	32	0,00014019
GO:0015280	amiloride-sensitive sodium channel activity	7	7	0,000170256
GO:0003810	protein-glutamine gamma-glutamyltransferase activity	7	7	0,000170256
GO:0048531	beta-1,3-galactosyltransferase activity	7	7	0,000170256
GO:0031324	negative regulation of cellular metabolism	61	182	0,000177619
GO:0008285	negative regulation of cell proliferation	29	69	0,000181098
GO:0005496	steroid binding	21	44	0,000181402
GO:0007498	mesoderm development	15	27	0,000197086
GO:0000139	Golgi membrane	24	54	0,000274643
GO:0051248	negative regulation of protein metabolism	23	51	0,00028811
GO:0001501	skeletal development	43	119	0,000349913
GO:0008194	UDP-glycosyltransferase activity	32	81	0,000382612
GO:0019932	second-messenger-mediated signaling	32	82	0,000532172
GO:0001558	regulation of cell growth	26	62	0,000532172
GO:0007420	brain development	33	86	0,00063864
GO:0005520	insulin-like growth factor binding	12	19	0,000642723
GO:0045934	negative regulation of nucleobase, nucleoside, nucleotide and nu	54	162	0,000648199
GO:0016563	transcriptional activator activity	45	129	0,00069886
GO:0016481	negative regulation of transcription	52	155	0,000718078
GO:0001609	adenosine receptor activity, G-protein coupled	6	6	0,000739463
GO:0018149	peptide cross-linking	6	6	0,000739463
GO:0004977	melanocortin receptor activity	6	6	0,000739463
GO:0001993	norepinephrine-epinephrine regulation of blood pressure	6	6	0,000739463
GO:0008499	UDP-galactose:beta-N-acetylglucosamine beta-1,3-galactosyltra	6	6	0,000739463
GO:0004936	alpha-adrenergic receptor activity	6	6	0,000739463
GO:0048332	mesoderm morphogenesis	9	12	0,000744496
GO:0015081	sodium ion transporter activity	19	41	0,000832039
GO:0003954	NADH dehydrogenase activity	19	41	0,000832039
GO:0050136	NADH dehydrogenase (quinone) activity	19	41	0,000832039
GO:0008137	NADH dehydrogenase (ubiquinone) activity	19	41	0,000832039
GO:0007188	G-protein signaling, coupled to cAMP nucleotide second messeng	18	38	0,000844756
GO:0001503	ossification	27	67	0,000939064

GO:0031214	biomineral formation	27	67	0,000939064
GO:0007182	common-partner SMAD protein phosphorylation	7	8	0,000939064
GO:0005066	transmembrane receptor protein tyrosine kinase signaling protei	7	8	0,000939064
GO:0046849	bone remodeling	29	74	0,001027967
GO:0008201	heparin binding	22	51	0,00108433
GO:0019838	growth factor binding	19	42	0,001345313
GO:0051129	negative regulation of cell organization and biogenesis	16	33	0,001428979
GO:0016655	oxidoreductase activity, acting on NADH or NADPH, quinone or s	19	43	0,002123007
GO:0019933	cAMP-mediated signaling	19	43	0,002123007
GO:0051726	regulation of cell cycle	82	279	0,002127269
GO:0000074	regulation of progression through cell cycle	82	279	0,002127269
GO:0000119	mediator complex	12	21	0,002165532
GO:0008378	galactosyltransferase activity	13	25	0,002187417
GO:0031301	integral to organelle membrane	17	37	0,002350558
GO:0006874	calcium ion homeostasis	16	34	0,002407744
GO:0007267	cell-cell signaling	66	216	0,002428702
GO:0001990	regulation of blood pressure by hormones	8	11	0,002428702
GO:0030902	hindbrain development	8	11	0,002428702
GO:0001707	mesoderm formation	8	11	0,002428702
GO:0050886	endocrine physiological process	8	11	0,002428702
GO:0001976	fast regulation of arterial pressure	8	11	0,002428702
GO:0007167	enzyme linked receptor protein signaling pathway	59	189	0,002610964
GO:0048511	rhythmic process	21	50	0,002612522
GO:0008015	circulation	20	47	0,002875668
GO:0004994	somatostatin receptor activity	5	5	0,003053251
GO:0004952	dopamine receptor activity	5	5	0,003053251
GO:0001601	peptide YY receptor activity	5	5	0,003053251
GO:0004415	hyalurononglucosaminidase activity	5	5	0,003053251
GO:0035240	dopamine binding	5	5	0,003053251
GO:0016918	retinal binding	7	9	0,003073608
GO:0005543	phospholipid binding	19	44	0,003093229
GO:0005544	calcium-dependent phospholipid binding	11	19	0,00315942
GO:0007187	G-protein signaling, coupled to cyclic nucleotide second messen(	18	41	0,003352559
GO:0016011	dystroglycan complex	6	7	0,003491304
GO:0004908	interleukin-1 receptor activity	6	7	0,003491304
GO:0019966	interleukin-1 binding	6	7	0,003491304
GO:0016010	dystrophin-associated glycoprotein complex	6	7	0,003491304
GO:0051261	protein depolymerization	16	35	0,003725201
GO:0035282	segmentation	15	32	0,003844065
GO:0008021	synaptic vesicle	20	48	0,004091718
GO:0008217	blood pressure regulation	11	20	0,005199173
GO:0001704	formation of primary germ layer	8	12	0,005199173
GO:0030693	caspase activity	8	12	0,005199173
GO:0006469	negative regulation of protein kinase activity	12	23	0,005550726
GO:0000122	negative regulation of transcription from RNA polymerase II pro	30	84	0,00607525
GO:0019935	cyclic-nucleotide-mediated signaling	19	46	0,006360501
GO:0050817	coagulation	19	46	0,006360501
GO:0001569	patterning of blood vessels	9	15	0,006824188
GO:0048646	anatomical structure formation	9	15	0,006824188
GO:0008284	positive regulation of cell proliferation	29	81	0,006949148
GO:0051093	negative regulation of development	21	53	0,006999387
GO:0030514	negative regulation of BMP signaling pathway	7	10	0,007170772
GO:0030672	synaptic vesicle membrane	7	10	0,007170772
GO:0019840	isoprenoid binding	7	10	0,007170772
GO:0005501	retinoid binding	7	10	0,007170772
GO:0045667	regulation of osteoblast differentiation	7	10	0,007170772
GO:0007179	transforming growth factor beta receptor signaling pathway	17	40	0,007662151
GO:0016758	transferase activity, transferring hexosyl groups	39	119	0,008380411
GO:0051348	negative regulation of transferase activity	12	24	0,008388384
GO:0007599	hemostasis	19	47	0,008837014
GO:0001764	neuron migration	15	34	0,009070026
GO:0050878	regulation of body fluids	21	54	0,009466257
GO:0004945	angiotensin type II receptor activity	6	8	0,00961483
GO:0001595	angiotensin receptor activity	6	8	0,00961483
GO:0009948	anterior/posterior axis specification	6	8	0,00961483
GO:0035295	tube development	34	101	0,009778851
GO:0040012	regulation of locomotion	18	44	0,009778851

**deuterostomia**				
GO:0004931	ATP-gated cation channel activity	5	6	4,74E-05
GO:0009607	response to biotic stimulus	45	979	0,00100739
GO:0006952	defense response	44	950	0,00100739
GO:0004800	thyroxine 5'-deiodinase activity	3	3	0,002093473
GO:0030106	MHC class I receptor activity	5	15	0,002209497
GO:0006955	immune response	35	736	0,002495027
GO:0030178	negative regulation of Wnt receptor signaling pathway	4	9	0,003585659
GO:0042981	regulation of apoptosis	16	246	0,003971402
GO:0008430	selenium binding	6	29	0,004113225
GO:0008517	folic acid transporter activity	3	4	0,004113225

GO:0043067	regulation of programmed cell death	16	250	0,004324535
GO:0008219	cell death	23	434	0,005584392
GO:0012501	programmed cell death	22	407	0,005584392
GO:0016265	death	23	442	0,006736191
GO:0042612	MHC class I protein complex	4	12	0,006736191
GO:0030111	regulation of Wnt receptor signaling pathway	4	12	0,006736191
GO:0006817	phosphate transport	7	51	0,008990529
**cordata**				
GO:0005911	intercellular junction	38	131	5,96E-33
GO:0005921	gap junction	20	24	1,97E-29
GO:0030054	cell junction	38	164	2,28E-29
GO:0005922	connexon complex	17	18	2,57E-27
GO:0005243	gap-junction forming channel activity	17	18	2,57E-27
GO:0015285	connexon channel activity	17	18	2,57E-27
GO:0005923	tight junction	17	60	2,44E-14
GO:0016327	apicolateral plasma membrane	17	76	1,45E-12
GO:0043296	apical junction complex	17	76	1,45E-12
GO:0005615	extracellular space	74	2021	7,43E-10
GO:0005886	plasma membrane	60	1580	1,61E-08
GO:0004668	protein-arginine deiminase activity	5	5	1,25E-07
GO:0005164	tumor necrosis factor receptor binding	7	16	3,45E-07
GO:0016021	integral to membrane	132	4930	5,45E-07
GO:0031224	intrinsic to membrane	132	4932	5,45E-07
GO:0015268	alpha-type channel activity	20	360	4,69E-06
GO:0007267	cell-cell signaling	17	216	1,34E-05
GO:0016813	hydrolase activity, acting on carbon-nitrogen (but not peptide) b	5	10	1,68E-05
GO:0015267	channel or pore class transporter activity	20	381	1,94E-05
GO:0005102	receptor binding	24	507	2,32E-05
GO:0016020	membrane	145	6163	0,000441062
GO:0005125	cytokine activity	14	212	0,000865763
GO:0005540	hyaluronic acid binding	4	16	0,004439783
GO:0004953	icosanoid receptor activity	3	9	0,011335112
GO:0004954	prostanoid receptor activity	3	9	0,011335112
GO:0035050	embryonic heart tube development	3	11	0,01940706
GO:0006927	transformed cell apoptosis	2	3	0,02104147
GO:0001730	2'-5'-oligoadenylate synthetase activity	2	3	0,02104147
GO:0005539	glycosaminoglycan binding	6	66	0,023761608
GO:0030247	polysaccharide binding	6	73	0,036462296
GO:0001871	pattern binding	6	76	0,039712929
GO:0004960	thromboxane receptor activity	2	5	0,048939877
GO:0048535	lymph node development	3	17	0,049307599
GO:0004896	hematopoietin/interferon-class (D200-domain) cytokine recepto	5	57	0,051278229
GO:0007423	sensory organ development	3	19	0,061545214
GO:0005212	structural constituent of eye lens	3	23	0,090289671
GO:0004955	prostaglandin receptor activity	2	8	0,097500035
**vertebrata**				
GO:0005102	receptor binding	130	507	0
GO:0016503	pheromone receptor activity	59	111	0
GO:0005179	hormone activity	53	115	0
GO:0042221	response to chemical stimulus	90	329	9,81E-79
GO:0009628	response to abiotic stimulus	92	414	2,94E-59
GO:0005615	extracellular space	230	2021	1,24E-45
GO:0005550	pheromone binding	50	94	1,49E-38
GO:0005125	cytokine activity	52	212	5,02E-38
GO:0005549	odorant binding	50	99	3,45E-37
GO:0001664	G-protein-coupled receptor binding	36	47	3,23E-36
GO:0019236	response to pheromone	46	85	5,07E-36
GO:0042379	chemokine receptor binding	29	36	2,80E-30
GO:0008009	chemokine activity	29	36	2,80E-30
GO:0007626	locomotory behavior	38	152	2,32E-28
GO:0007610	behavior	46	228	6,80E-25
GO:0006954	inflammatory response	33	135	8,42E-24
GO:0006935	chemotaxis	36	94	1,05E-21
GO:0042330	taxis	36	94	1,05E-21
GO:0004896	hematopoietin/interferon-class (D200-domain) cytokine recepto	26	57	6,83E-18
GO:0005515	protein binding	250	3209	3,14E-16
GO:0004872	receptor activity	221	2793	7,52E-15
GO:0006955	immune response	80	736	5,50E-13
GO:0019965	interleukin binding	17	37	9,39E-12
GO:0009607	response to biotic stimulus	95	979	1,78E-11
GO:0005184	neuropeptide hormone activity	12	19	2,06E-10
GO:0004907	interleukin receptor activity	15	34	4,61E-10
GO:0016021	integral to membrane	321	4930	3,19E-09
GO:0031224	intrinsic to membrane	321	4932	3,23E-09
GO:0006952	defense response	88	950	3,23E-09
GO:0019955	cytokine binding	19	66	5,11E-09
GO:0009605	response to external stimulus	52	488	6,47E-08

GO:0046649	lymphocyte activation	19	112	1,99E-07
GO:0007218	neuropeptide signaling pathway	18	75	3,14E-07
GO:0045321	immune cell activation	20	125	4,51E-07
GO:0004869	cysteine protease inhibitor activity	11	27	4,98E-07
GO:0001775	cell activation	20	126	5,57E-07
GO:0005887	integral to plasma membrane	71	796	1,36E-06
GO:0031226	intrinsic to plasma membrane	71	798	1,50E-06
GO:0005886	plasma membrane	119	1580	4,53E-06
GO:0051239	regulation of organismal physiological process	22	158	4,84E-06
GO:0016020	membrane	366	6163	9,95E-06
GO:0050874	organismal physiological process	174	2571	1,77E-05
GO:0009611	response to wounding	40	392	1,88E-05
GO:0048534	hemopoietic or lymphoid organ development	20	149	4,45E-05
GO:0004888	transmembrane receptor activity	140	2007	4,67E-05
GO:0019884	antigen presentation, exogenous antigen	8	22	8,97E-05
GO:0042591	antigen presentation, exogenous antigen via MHC class II	6	12	0,000156827
GO:0019882	antigen presentation	10	40	0,000253649
GO:0050880	regulation of blood vessel size	7	19	0,000305609
GO:0051707	response to other organism	51	594	0,000372492
GO:0009613	response to pest, pathogen or parasite	49	570	0,000522173
GO:0042110	T cell activation	12	63	0,000655764
GO:0030097	hemopoiesis	17	133	0,000691646
GO:0030098	lymphocyte differentiation	11	54	0,000697702
GO:0050900	immune cell migration	7	22	0,000834677
GO:0045580	regulation of T cell differentiation	6	16	0,000997524
GO:0008015	circulation	10	47	0,001001543
GO:0007268	synaptic transmission	16	125	0,001123514
GO:0030593	neutrophil chemotaxis	5	11	0,001382084
GO:0030595	immune cell chemotaxis	6	17	0,00142073
GO:0004862	cAMP-dependent protein kinase inhibitor activity	3	3	0,001599137
GO:0050780	dopamine receptor binding	3	3	0,001599137
GO:0050863	regulation of T cell activation	9	41	0,001686827
GO:0045582	positive regulation of T cell differentiation	5	12	0,002088875
GO:0003956	NAD(P)+-protein-arginine ADP-ribosyltransferase activity	4	7	0,002207245
GO:0030217	T cell differentiation	7	26	0,002288288
GO:0019226	transmission of nerve impulse	17	144	0,002559127
GO:0015026	coreceptor activity	4	8	0,004065421
GO:0045619	regulation of lymphocyte differentiation	6	21	0,004483939
GO:0009986	cell surface	15	125	0,00484056
GO:0004857	enzyme inhibitor activity	19	177	0,005392091
GO:0019886	antigen processing, exogenous antigen via MHC class II	5	15	0,005889717
GO:0045621	positive regulation of lymphocyte differentiation	5	15	0,005889717
GO:0007173	epidermal growth factor receptor signaling pathway	4	9	0,006197448
GO:0050913	sensory perception of bitter taste	4	9	0,006197448
GO:0050870	positive regulation of T cell activation	7	32	0,007507915
GO:0050909	sensory perception of taste	6	24	0,008359414
GO:0030333	antigen processing	7	33	0,008816943
GO:0042101	T cell receptor complex	4	10	0,009244656

**mamalia**				
GO:0005615	extracellular space	198	2021	6,14E-53
GO:0005102	receptor binding	80	507	1,79E-46
GO:0005125	cytokine activity	48	212	1,79E-46
GO:0009607	response to biotic stimulus	104	979	1,03E-30
GO:0006952	defense response	102	950	1,03E-30
GO:0042742	defense response to bacteria	34	70	2,51E-28
GO:0009617	response to bacteria	34	78	2,22E-26
GO:0005126	hematopoietin/interferon-class (D200-domain) cytokine recepto	20	33	6,10E-19
GO:0008083	growth factor activity	26	141	2,98E-18
GO:0051707	response to other organism	60	594	1,67E-15
GO:0009613	response to pest, pathogen or parasite	54	570	5,26E-12
GO:0006955	immune response	61	736	5,48E-10
GO:0051239	regulation of organismal physiological process	22	158	8,97E-10
GO:0005179	hormone activity	23	115	1,11E-09
GO:0005149	interleukin-1 receptor binding	9	12	1,13E-09
GO:0030547	receptor inhibitor activity	7	7	4,23E-09
GO:0050909	sensory perception of taste	11	24	1,04E-08
GO:0031424	keratinization	11	25	1,73E-08
GO:0005152	interleukin-1 receptor antagonist activity	6	6	7,37E-08
GO:0030353	fibroblast growth factor receptor antagonist activity	6	6	7,37E-08
GO:0048019	receptor antagonist activity	6	6	7,37E-08
GO:0030545	receptor regulator activity	7	9	8,71E-08
GO:0045321	immune cell activation	21	125	1,26E-07
GO:0001775	cell activation	21	126	1,40E-07
GO:0050865	regulation of cell activation	14	54	1,40E-07
GO:0051249	regulation of lymphocyte activation	14	54	1,40E-07
GO:0050863	regulation of T cell activation	12	41	3,69E-07
GO:0046649	lymphocyte activation	19	112	4,90E-07
GO:0009913	epidermal cell differentiation	11	35	6,13E-07

GO:0042110	T cell activation	14	63	1,02E-06
GO:0050776	regulation of immune response	17	95	1,12E-06
GO:0048730	epidermis morphogenesis	11	40	2,58E-06
GO:0050870	positive regulation of T cell activation	10	32	2,58E-06
GO:0009986	cell surface	19	125	2,59E-06
GO:0050867	positive regulation of cell activation	11	41	3,11E-06
GO:0051251	positive regulation of lymphocyte activation	11	41	3,11E-06
GO:0009888	tissue development	20	185	7,81E-06
GO:0042098	T cell proliferation	9	30	1,32E-05
GO:0050670	regulation of lymphocyte proliferation	9	30	1,32E-05
GO:0042129	regulation of T cell proliferation	8	23	1,53E-05
GO:0006950	response to stress	62	949	2,15E-05
GO:0009897	external side of plasma membrane	14	84	3,06E-05
GO:0008544	epidermis development	12	62	3,19E-05
GO:0046651	lymphocyte proliferation	10	42	3,19E-05
GO:0048729	tissue morphogenesis	11	52	3,36E-05
GO:0007398	ectoderm development	12	66	6,21E-05
GO:0005882	intermediate filament	14	90	6,70E-05
GO:0006915	apoptosis	32	401	7,05E-05
GO:0045111	intermediate filament cytoskeleton	14	91	7,47E-05
GO:0045937	positive regulation of phosphate metabolism	6	14	7,93E-05
GO:0042327	positive regulation of phosphorylation	6	14	7,93E-05
GO:0050731	positive regulation of peptidyl-tyrosine phosphorylation	6	14	7,93E-05
GO:0012501	programmed cell death	32	407	0,000102671
GO:0050671	positive regulation of lymphocyte proliferation	7	22	0,000124506
GO:0008219	cell death	33	434	0,000182007
GO:0045764	positive regulation of amino acid metabolism	6	16	0,000183949
GO:0001934	positive regulation of protein amino acid phosphorylation	6	16	0,000183949
GO:0050778	positive regulation of immune response	11	65	0,00027354
GO:0016265	death	33	442	0,000291344
GO:0048302	regulation of isotype switching to IgG isotypes	4	6	0,000322606
GO:0048304	positive regulation of isotype switching to IgG isotypes	4	6	0,000322606
GO:0048291	isotype switching to IgG isotypes	4	6	0,000322606
GO:0042102	positive regulation of T cell proliferation	6	18	0,000372997
GO:0050864	regulation of B cell activation	7	27	0,000488499
GO:0042506	tyrosine phosphorylation of Stat5 protein	3	3	0,000602435
GO:0051247	positive regulation of protein metabolism	9	48	0,000604119
GO:0042981	regulation of apoptosis	21	246	0,000807096
GO:0042113	B cell activation	9	50	0,000807096
GO:0043067	regulation of programmed cell death	21	250	0,00111149
GO:0051240	positive regulation of organismal physiological process	11	77	0,001138946
GO:0045830	positive regulation of isotype switching	4	8	0,001213937
GO:0045191	regulation of isotype switching	4	8	0,001213937
GO:0030098	lymphocyte differentiation	9	54	0,001418321
GO:0018108	peptidyl-tyrosine phosphorylation	8	45	0,001977793
GO:0018212	peptidyl-tyrosine modification	8	45	0,001977793
GO:0045190	isotype switching	4	9	0,001977793
GO:0051047	positive regulation of secretion	3	4	0,001977793
GO:0005164	tumor necrosis factor receptor binding	5	16	0,002022878
GO:0005104	fibroblast growth factor receptor binding	6	25	0,002261225
GO:0007260	tyrosine phosphorylation of STAT protein	4	10	0,003069003
GO:0048518	positive regulation of biological process	34	511	0,00316109
GO:0048522	positive regulation of cellular process	30	434	0,003308674
GO:0030097	hemopoiesis	14	133	0,003308674
GO:0050730	regulation of peptidyl-tyrosine phosphorylation	6	27	0,003320031
GO:0043119	positive regulation of physiological process	29	416	0,003440774
GO:0048534	hemopoietic or lymphoid organ development	14	149	0,003440774
GO:0009615	response to virus	7	38	0,003553886
GO:0051242	positive regulation of cellular physiological process	28	400	0,003992871
GO:0001673	male germ cell nucleus	3	5	0,004129988
GO:0042104	positive regulation of activated T cell proliferation	3	5	0,004129988
GO:0050853	B cell receptor signaling pathway	3	5	0,004129988
GO:0050871	positive regulation of B cell activation	5	20	0,005463151
GO:0008284	positive regulation of cell proliferation	10	81	0,005967926
GO:0005515	protein binding	149	3209	0,006200022
GO:0046006	regulation of activated T cell proliferation	3	6	0,007518942
GO:0050798	activated T cell proliferation	3	6	0,007518942
GO:0051241	negative regulation of organismal physiological process	6	32	0,007556851
GO:0001932	regulation of protein amino acid phosphorylation	6	32	0,007556851
GO:0006521	regulation of amino acid metabolism	6	32	0,007556851
GO:0042325	regulation of phosphorylation	6	34	0,009843539
GO:0051174	regulation of phosphorus metabolism	6	34	0,009843539
GO:0019220	regulation of phosphate metabolism	6	34	0,009843539
GO:0050851	antigen receptor-mediated signaling pathway	4	14	0,009843539
GO:0045058	T cell selection	4	14	0,009843539
GO:0050829	defense response to Gram-negative bacteria	2	2	0,009843539
GO:0005138	interleukin-6 receptor binding	2	2	0,009843539
GO:0030502	negative regulation of bone mineralization	2	2	0,009843539
GO:0050832	defense response to fungi	2	2	0,009843539

GO:0001781	neutrophil apoptosis	2	2	0,009843539
GO:0030345	structural constituent of tooth enamel	2	2	0,009843539
GO:0042523	positive regulation of tyrosine phosphorylation of Stat5 protein	2	2	0,009843539
GO:0030021	extracellular matrix structural constituent conferring compressio	2	2	0,009843539
GO:0001780	neutrophil homeostasis	2	2	0,009843539
GO:0042522	regulation of tyrosine phosphorylation of Stat5 protein	2	2	0,009843539
