# Supplementary material for: New genes in the evolution of the neural crest differentiation program
Source: Genome Biol. 2007 Mar 12;8(3):R36. doi: 10.1186/gb-2007-8-3-r36 (PMC1868935; doi:10.1186/gb-2007-8-3-r36)
Supplement: Additional data file 2 — The table comprises a full list of the 615 neural crest genes compiled using Phenotype Ontology annotations for each of the seven temporal categories considered in this work: prokaryota (pro), eukaryota (euk), metazoa (met), deuterostomia (deu), chordata (cor), vertebrata (ver), and mammalia (mam). [file gb-2007-8-3-r36-S2.pdf]

Prokaryota

3'-phosphoadenosine 5'-phosphosulfate synthase 2
3-phosphoinositide dependent protein kinase-1
6-pyruvoyl-tetrahydropterin synthase
acid phosphatase 2, lysosomal
acid phosphatase 5, tartrate resistant
activin A receptor, type II-like 1
activin receptor IIA
activin receptor IIB
adenomatosis polyposis coli
adenosine deaminase
aldehyde dehydrogenase family 1, subfamily A2
alkaline phosphatase 2, liver
ankyrin 1, erythroid
apoptotic peptidase activating factor 1
aryl hydrocarbon receptor nuclear translocator
arylsulfatase B
aspartate-beta-hydroxylase
aspartoacylase (aminoacylase) 2
ATPase, Cu++ transporting, alpha polypeptide
ATP-binding cassette, sub-family A (ABC1), member 4
ATP-binding cassette, sub-family D (ALD), member 1
ATP/GTP binding protein 1
attractin
AXL receptor tyrosine kinase
Bardet-Biedl syndrome 2 homolog (human)
Bardet-Biedl syndrome 4 homolog (human)
biglycan
bone morphogenetic protein 1
bone morphogenetic protein receptor, type 1A
bone morphogenetic protein receptor, type 1B
breast cancer 1
cadherin EGF LAG seven-pass G-type receptor 1
calcium channel, voltage-dependent, L type, alpha 1S subunit
calcium-sensing receptor
carbonic anhydrase 2
cathepsin K
chloride channel 3
chloride channel 5
chloride channel 7
choline acetyltransferase
c-mer proto-oncogene tyrosine kinase
conserved helix-loop-helix ubiquitous kinase
CREB binding protein
crystallin, alpha B
c-src tyrosine kinase
cyclic nucleotide gated channel alpha 3
cyclic nucleotide gated channel beta 1b
cystic fibrosis transmembrane conductance regulator homolog
cytochrome P450, family 19, subfamily a, polypeptide 1
cytochrome P450, family 26, subfamily a, polypeptide 1
cytochrome P450, family 26, subfamily b, polypeptide 1
cytochrome P450, family 27, subfamily b, polypeptide 1
cytotoxic granule-associated RNA binding protein 1
decorin
deleted in colorectal carcinoma
dentin matrix protein 1
dentin sialophosphoprotein
discoidin domain receptor family, member 1
discs, large homolog 1 (Drosophila)
dopachrome tautomerase
dystonin
dystrophin, muscular dystrophy
early B-cell factor 3
enamelin
endothelin converting enzyme 1
Eph receptor A7
epidermal growth factor receptor
excision repair cross-complementing rodent repair deficiency, complementation group 2
excision repaiross-complementing rodent repair deficiency, complementation group 8

fibroblast growth factor receptor 1
fibroblast growth factor receptor 2
fibromodulin
FK506 binding protein 8
gamma-aminobutyric acid (GABA-A) receptor, subunit beta 3
gamma-glutamyltransferase 1
glucosidase, alpha, acid
glucosidase, beta, acid
glucuronyl C5-epimerase
glutathione S-transferase, alpha 4
glyceronephosphate O-acyltransferase
GNAS (guanine nucleotide binding protein, alpha stimulating) complex locus
G protein-coupled receptpr kinase 1
growth factor receptor bound protein 2-associated protein 2
gulonolactone (L-) oxidase
hedgehog acyltransferase
hexosaminidase A
hexosaminidase B
histone deacetylase 4
huntingtin interacting protein 1
huntingtin interacting protein 1 related
inhibitor of kappaB kinase beta
insulin-like growth factor I receptor
integrin alpha V
integrin linked kinase
keratin complex 2, basic, gene 17
kit oncogene
klotho
lamin A
lamin B1
laminin, alpha 3
laminin, alpha 5
lecithin-retinol acyltransferase (phosphatidylcholine-retinol-O-acyltransferase)
low density lipoprotein receptor-related protein 5
low density lipoprotein receptor-related protein 6
lumican
matrilin 1, cartilage matrix protein 1
matrix metallopeptidase 13
matrix metallopeptidase 14 (membrane-inserted)
matrix metallopeptidase 2
matrix metallopeptidase 20 (enamelysin)
matrix metallopeptidase 3
matrix metallopeptidase 9
mesoderm specific transcript
mitogen activated protein kinase kinase kinase 3
Musashi homolog 1(Drosophila)
myeloid/lymphoid or mixed-lineage leukemia
myosin, heavy polypeptide 1, skeletal muscle, adult
myosin Va
myosin XV
natriuretic peptide receptor 2
neuraminidase 1
neurofilament 3, medium
neurofilament, light polypeptide
neurotrophic tyrosine kinase, receptor, type 2
neurotrophic tyrosine kinase, receptor, type 3
N-myc downstream regulated gene 1
N-sulfoglucosamine sulfohydrolase (sulfamidase)
nuclear factor of kappa light polypeptide gene enhancer in B-cells inhibitor, epsilon
pantothenate kinase 2 (Hallervorden-Spatz syndrome)
patched homolog 1
periaxin
perlecan (heparan sulfate proteoglycan 2)
peroxisome biogenesis factor 7
phosphate regulating gene with homologies to endopeptidases on the X chromosome (hypophosphatemia, vitamin D resistant rickets)
phosphodiesterase 6B, cGMP, rod receptor, beta polypeptide
phospholipase C, beta 4
pink-eyed dilution
plasminogen activator, tissue
plasminogen activator, urokinase

platelet derived growth factor receptor, alpha polypeptide
plexin A3
plexin A4
polo-like kinase 2 (Drosophila)
polycystic kidney disease 1 homolog
potassium inwardly-rectifying channel, subfamily J, member 2
potassium voltage-gated channel, subfamily Q, member 1
pregnancy-associated plasma protein A
procollagen, type I, alpha 1
procollagen, type II, alpha 1
procollagen, type IX, alpha 1
progressive ankylosis
proprotein convertase subtilisin/kexin type 6
protective protein for beta-galactosidase
protein kinase, cGMP-dependent, type II
protocadherin 15
PTK7 protein tyrosine kinase 7
Rab38, member of RAS oncogene family
Rab geranylgeranyl transferase, a subunit
RAD50 homolog (S. cerevisiae)
receptor-interacting serine-threonine kinase 4
receptor-like tyrosine kinase
receptor tyrosine kinase-like orphan receptor 1
receptor tyrosine kinase-like orphan receptor 2
retinal pigment epithelium 65
retinol binding protein 3, interstitial
ret proto-oncogene
ribosomal protein S6 kinase polypeptide 3
Rous sarcoma oncogene
ryanodine receptor 1, skeletal muscle
sodium channel, voltage-gated, type VIII, alpha
solute carrier family 12, member 2
solute carrier family 12, member 6
solute carrier family 12, member 7
solute carrier family 26, member 4
solute carrier family 30 (zinc transporter), member 5
solute carrier family 34 (sodium phosphate), member 1
solute carrier family 37 (glycerol-6-phosphate transporter), member 4
solute carrier family 3, member 1
solute carrier family 45, member 2
solute carrier family 5 (choline transporter), member 7
solute carrier family 6 (neurotransmitter transporter, dopamine), member 3
solute carrier family 6 (neurotransmitter transporter, taurine), member 6
solute carrier family 9 (sodium/hydrogen exchanger), isoform 3 regulator 1
Son of sevenless homolog 1 (Drosophila)
spastic paraplegia 7 homolog (human)
spectrin alpha 1
spectrin beta 2
sphingomyelin phosphodiesterase 3, neutral
splicing factor 3b, subunit 1
stathmin 1
sterol-C5-desaturase (fungal ERG3, delta-5-desaturase) homolog (S. cerevisae)
SWI/SNF-related, matrix-associated actin-dependent regulator of chromatin, subfamily a, containing DEAD/H box 1`
SWI/SNF related, matrix associated, actin dependent regulator of chromatin, subfamily a, member 2
T-cell, immune regulator 1
tetratricopeptide repeat domain 7
thrombospondin 1
thrombospondin 2
thrombospondin 3
thymoma viral proto-oncogene 1
thymoma viral proto-oncogene 2
topoisomerase (DNA) II beta
transforming, acidic coiled-coil containing protein 3
tubulin-specific chaperone e
TYRO3 protein tyrosine kinase 3
tyrosinase
tyrosinase-related protein 1
utrophin
v-erb-b2 erythroblastic leukemia viral oncogene homolog 2, neuro/glioblastoma derived oncogene homolog (avian)
v-raf murine sarcoma 3611 viral oncogene homolog

whirlin
X-ray repair complementing defective repair in Chinese hamster cells 2
Yamaguchi sarcoma viral (v-yes) oncogene homolog 1
Y box protein 1
zeta-chain (TCR) associated protein kinase

Eukaryota
actin, alpha 1, skeletal muscle
activating transcription factor 2
adaptor-related protein complex 3, beta 1 subunit
adaptor-related protein complex 3, delta 1 subunit
a disintegrin and metallopeptidase domain 17
a disintegrin and metallopeptidase domain 19 (meltrin beta)
a disintegrin-like and metallopeptidase (reprolysin type) with thrombospondin type 1 motif, 20
aristaless 4
aryl hydrocarbon receptor-interacting protein-like 1
ataxin 7
ATX1 (antioxidant protein 1) homolog 1 (yeast)
budding uninhibited by benzimidazoles 1 homolog, beta (S. cerevisiae)
calcium binding protein 4
calponin 1
cartilage homeo protein 1
catenin (cadherin associated protein), beta 1, 88kDa
cathepsin D
ceroid lipofuscinosis, neuronal 3, juvenile (Batten, Spielmeyer-Vogt disease)
choroidermia
cofilin 1, non-muscle
crumbs homolog 1 (Drosophila)
cyclin D1
cyclin F
cystinosis, nephropathic
deformed epidermal autoregulatory factor 1 (Drosophila)
delta-like 3 (Drosophila)
dishevelled 2, dsh homolog (Drosophila)
distal-less homeobox 5
distal-less homeobox 6
early growth response 2
empty spiracles homolog 2 (Drosophila)
even skipped homeotic gene 2 homolog
fibulin 5
forkhead box A2
forkhead box C1
forkhead box C2
forkhead box F2
forkhead box G1
forkhead box I1
forkhead box N1
forkhead box N4
GATA binding protein 3
GLI-Kruppel family member GLI1
GLI-Kruppel family member GLI3
growth factor receptor bound protein 2
guanine nucleotide binding protein, alpha q polypeptide
homeo box A10
homeo box A11
homeo box B7
homeo box B8
homeo box C8
homeo box D11
homeo box D12
homeo box gene expressed in ES cells
homeo box, msh-like 1
homeo box, msh-like 2
hypermethylated in cancer 1
inositol polyphosphate phosphatase-like 1
integrin beta 3
jagged 2
Jun-B oncogene
Jun oncogene
kinesin family member 1B
Kruppel-like factor 10

Kruppel-like factor 7 (ubiquitous)
latent transforming growth factor beta binding protein 3
LIM domain only 4
LIM homeobox protein 1
LIM homeobox protein 8
LIM homeobox transcription factor 1 alpha
LIM homeobox transcription factor 1 beta
lymphoid enhancer binding factor 1
lysosomal trafficking regulator
mahogunin, ring finger 1
matrilin 2
matrilin 3
mesenchyme homeobox 1
microphthalmia-associated transcription factor
myeloid/lymphoid or mixed lineage-leukemia translocation to 3 homolog (Drosophila)
myosin VIIa
myotubularin related protein 2
MYST histone acetyltransferase monocytic leukemia 4
neurofibromatosis 1
odd-skipped related 2 (Drosophila)
orthodenticle homolog 1 (Drosophila)
orthodenticle homolog 2 (Drosophila)
paired box gene 3
paired box gene 6
paired box gene 7
paired-like homeodomain transcription factor 3
paired related homeobox protein-like 1
palmitoyl-protein thioesterase 1
peroxisomal membrane protein 3
phosphatidylinositol-4-phosphate 5-kinase, type II, beta
phospholipase C, gamma 2
polycomb group ring finger 2
polycomb group ring finger 4
polymerase (DNA directed), gamma
POU domain, class 3, transcription factor 4
POU domain, class 4, transcription factor 1
pre B-cell leukemia transcription factor 1
protein tyrosine phosphatase, non-receptor type 11
protein tyrosine phosphatase, receptor type, E
RAD23b homolog (S. cerevisiae)
RAS p21 protein activator 1
retina and anterior neural fold homeobox
ring finger protein 1
ring finger protein 2
sal-like 3 (Drosophila)
sequestosome 1
serum response factor
sine oculis-related homeobox 1 homolog (Drosophila)
sine oculis-related homeobox 3 homolog (Drosophila)
SMAD specific E3 ubiquitin protein ligase 1
snail homolog 2 (Drosophila)
solute carrier family 4 (anion exchanger), member 2
solute carrier family 4, sodium bicarbonate cotransporter, member 7
SRY-box containing gene 10
SRY-box containing gene 18
SRY-box containing gene 3
SRY-box containing gene 5
SRY-box containing gene 8
SRY-box containing gene 9
synaptojanin 1
synovial apoptosis inhibitor 1, synoviolin
syntaxin binding protein 1
Tnf receptor-associated factor 6
trans-acting transcription factor 3
trans-acting transcription factor 7
trans-acting transcription factor 8
transcription factor A, mitochondrial
transcription factor E3
tubby-like protein 3
ubiquitin carboxy-terminal hydrolase L1

Unc4.1 homeobox (C. elegans)
vacuolar protein sorting 33A (yeast)
v-crk sarcoma virus CT10 oncogene homolog (avian)-like
Wiskott-Aldrich syndrome homolog (human)
xeroderma pigmentosum, complementation group A
zinc finger protein 36
zinc finger protein 53
zinc finger protein 98
zinc finger protein of the cerebellum 1
**Metazoa**
achaete-scute complex homolog-like 1 (Drosophila)
activated leukocyte cell adhesion molecule
a disintegrin-like and metallopeptidase (reprolysin type) with thrombospondin type 1 motif, 2
a disintegrin-like and metallopeptidase (reprolysin type) with thrombospondin type 1 motif, 5 (aggrecanase-2)
adrenergic receptor, beta 1
adrenergic receptor, beta 2
amino-terminal enhancer of split
androgen receptor
annexin A7
aristaless 3
avian musculoaponeurotic fibrosarcoma (v-maf) AS42 oncogene homolog
axin 1
axin2
bagpipe homeobox gene 1 homolog (Drosophila)
BarH-like 1 (Drosophila)
BarH-like homeobox 2
B-cell translocation gene 2, anti-proliferative
Bcl2-associated X protein
bone morphogenetic protein 2
bone morphogenetic protein 3
bone morphogenetic protein 4
bone morphogenetic protein 5
bone morphogenetic protein 6
bone morphogenetic protein 7
calcitonin receptor
calcium channel, voltage-dependent, beta 1 subunit
calcium channel, voltage-dependent, beta 2 subunit
cappuccino
caspase 2
caspase 3
caudal type homeo box 1
caudal type homeo box 2
CCAAT/enhancer binding protein (C/EBP), epsilon
CD 81 antigen
CD9 antigen
C. elegans ceh-10 homeo domain containing homolog
cell adhesion molecule-related/down-regulated by oncogenes
cellular retinoic acid binding protein II
cholinergic receptor, nicotinic, alpha polypeptide 9
chordin
chromobox homolog 2 (Drosophila Pc class)
colony stimulating factor 1 (macrophage)
connective tissue growth factor
core binding factor beta
cripto, FRL-1, cryptic family 1
C-type lectin domain family 3, member b
cyclic nucleotide phosphodiesterase 1
cyclin-dependent kinase inhibitor 1B (P27)
cyclin-dependent kinase inhibitor 1C (P57)
dishevelled, dsh homolog 1 (Drosophila)
dopamine receptor 2
dopamine receptor 5
dystrobrevin alpha
E1A binding protein p300
E2F transcription factor 4
E2F transcription factor 5
early B-cell factor 2
ectodysplasin-A
endothelial differentiation, lysophosphatidic acid G-protein-coupled receptor, 2
endothelin receptor type A

endothelin receptor type B
engrailed 1
ephrin A5
eyes absent 1 homolog (Drosophila)
fibroblast growth factor 18
fibroblast growth factor 6
fibroblast growth factor 8
follistatin
glial cell line derived neurotrophic factor family receptor alpha 1
glial cell line derived neurotrophic factor family receptor alpha 2
glucose-6-phosphatase, catalytic
glypican 3
G protein coupled receptor 24
gremlin 1
growth differentiation factor 5
growth differentiation factor 6
hairy and enhancer of split 1 (Drosophila)
hairy and enhancer of split 5 (Drosophila)
hairy and enhancer of split 7 (Drosophila)
heart and neural crest derivatives expressed transcript 2
heat shock protein 2
Hermansky-Pudlak syndrome 3 homolog (human)
Hermansky-Pudlak syndrome 5 homolog (human)
homeo box A1
homeo box A2
homeo box A9
homeo box B1
homeo box B13
homeo box B3
homeo box B4
homeo box B5
homeo box B6
homeo box B9
homeo box D13
Indian hedgehog
inhibin alpha
inhibin beta-A
inhibin beta-B
insulin receptor substrate 2
interleukin 8 receptor, beta
leukemia inhibitory factor receptor
limb and neural patterns
limb region 1
LIM domain binding 1
LIM homeobox protein 2
lunatic fringe gene homolog (Drosophila)
lysosomal membrane glycoprotein 1
lysosomal membrane glycoprotein 2
mab-21-like 2 (C. elegans)
MAD homolog 2 (Drosophila)
MAD homolog 3 (Drosophila)
mannoside acetylglucosaminyltransferase 2
max binding protein
melanocortin 4 receptor
melanophilin
mesenchyme homeobox 2
mesoderm posterior 2
mesogenin 1
myogenic factor 5
myogenic factor 6
myogenin
nerve growth factor receptor (TNFR superfamily, member 16)
neural retina leucine zipper gene
neuroblastoma, suppression of tumorigenicity 1
neurochondrin
neurogenic differentiation 4
neuropilin 1
NK2 transcription factor related, locus 5 (Drosophila)
NK6 transcription factor related, locus 2 (Drosophila)
nuclear factor of activated T-cells, cytoplasmic, calcineurin-dependent 2

nuclear factor of activated T-cells, cytoplasmic, calcineurin-dependent 3
nuclear factor of activated T-cells, cytoplasmic, calcineurin-dependent 4
nuclear receptor subfamily 2, group E, member 3
nuclear receptor subfamily 2, group F, member 2
nuclear receptor subfamily 3, group C, member 1
occludin
osteopetrosis associated transmembrane protein 1
paired box gene 9
peripherin 1
platelet derived growth factor, alpha
platelet-derived growth factor, C polypeptide
polyhomeotic-like 1 (Drosophila)
presenilin 1
proteoglycan 4 (megakaryocyte stimulating factor, articular superficial zone protein)
radical fringe gene homolog (Drosophila)
retinaldehyde binding protein 1
retinitis pigmentosa 1 homolog (human)
retinitis pigmentosa GTPase regulator interacting protein 1
retinoblastoma 1
retinoblastoma-like 1 (p107)
retinoblastoma-like 2
retinoic acid induced 1
retinoic acid receptor, alpha
retinoic acid receptor, beta
retinoic acid receptor, gamma
retinoid X receptor alpha
retinol binding protein 1, cellular
retinoschisis 1 homolog (human)
rhodopsin
runt related transcription factor 2
secreted acidic cysteine rich glycoprotein
secreted frizzled-related sequence protein 1
sema domain, immunoglobulin domain (Ig), short basic domain, secreted, (semaphorin) 3A
sema domain, immunoglobulin domain (Ig), short basic domain, secreted, (semaphorin) 3C
sema domain, immunoglobulin domain (Ig), transmembrane domain (TM) and short cytoplasmic domain, (semaphorin) 4A
serine peptidase inhibitor, Kazal type 5
SFFV proviral integration 1
shroom
sine oculis-related homeobox 4 homolog (Drosophila)
Sloan-Kettering viral oncogene homolog
smoothened homolog (Drosophila)
sonic hedgehog
sprouty homolog 2 (Drosophila)
sprouty protein with EVH-1 domain 1, related sequence
src homology 2 domain-containing transforming protein C2
src homology 2 domain-containing transforming protein C3
SRY-box containing gene 11
SRY-box containing gene 6
T-box 1
T-box 15
T-box18
T-cell acute lymphocytic leukemia 1
T-cell leukemia, homeobox 1
thyroid hormone receptor beta
Tnf receptor associated factor 4
transcription factor AP-2, alpha
transcription factor EC
transducer of ErbB-2.1
transformation related protein 63
transforming growth factor, beta 2
transforming growth factor, beta 3
transglutaminase 1, K polypeptide
tumor necrosis factor, alpha-induced protein 3
tumor necrosis factor receptor superfamily, member 1a
twisted gastrulation homolog 1 (Drosophila)
twist gene homolog 1 (Drosophila)
twist homolog 2 (Drosophila)
UDP-GlcNAc:betaGal beta-1,3-N-acetylglucosaminyltransferase 1
Usher syndrome 1C homolog (human)
vascular endothelial growth factor A

vitamin D receptor
v-myc myelocytomatosis viral related oncogene, neuroblastoma derived (avian)
wingless-related MMTV integration site 3A
wingless-related MMTV integration site 5A
wingless-related MMTV integration site 7A

Deuterostomia
brain derived neurotrophic factor
Fanconi anemia, complementation group A
fos-like antigen 2
neurotrophin 3
noggin
purinergic receptor P2X, ligand-gated ion channel, 7
rod outer segment membrane protein 1

Chordata
activating transcription factor 4
Cbp/p300-interacting transactivator, with Glu/Asp-rich carboxy-terminal domain, 2
claudin 14
epilepsy, progressive myoclonic epilepsy, type 2 gene alpha
fos-like antigen 1
gap junction membrane channel protein beta 6
hyaluronan and proteoglycan link protein 1
transforming growth factor, beta receptor III

Vertebrata
BCL2-like 11 (apoptosis facilitator)
calcitonin/calcitonin-related polypeptide, alpha
chemokine (C-C motif) receptor 10
cocaine and amphetamine regulated transcript
endothelin 1
endothelin 3
formin 1
glial cell line derived neurotrophic factor
gonadotropin releasing hormone 1
Hermansky-Pudlak syndrome 6
integrin, alpha 10
islet amyloid polypeptide
leukocyte cell derived chemotaxin 1
matrix Gla protein
melanoma inhibitory activity 1
myelin protein zero
natriuretic peptide precursor type C
neuregulin 1
neurturin
parathyroid hormone
parathyroid hormone-like peptide
phosphodiesterase 6G, cGMP-specific, rod, gamma
pro-opiomelanocortin-alpha
silver
tenomodulin
Treacher Collins Franceschetti syndrome 1, homolog

Mammalia
adrenocortical dysplasia
ameloblastin
amelogenin X chromosome
BH3 interacting domain death agonist
colony stimulating factor 2 (granulocyte-macrophage)
harakiri, BCL2 interacting protein (contains only BH3 domain)
kit ligand
leptin
matrix extracellular phosphoglycoprotein with ASARM motif (bone)
MyoD family inhibitor
nonagouti
oncostatin M
programmed cell death 1
TYRO protein tyrosine kinase binding protein
