# Supplementary material for: New genes in the evolution of the neural crest differentiation program
Source: Genome Biol. 2007 Mar 12;8(3):R36. doi: 10.1186/gb-2007-8-3-r36 (PMC1868935; doi:10.1186/gb-2007-8-3-r36)
Supplement: Additional data file 3 — The table shows statistically over-represented GO annotations of the set of neural crest developmental genes that emerged in vertebrates (cutoff P < 0.001). [file gb-2007-8-3-r36-S3.pdf]

GO ID	GO term	count sam	count total	P-Value
**ver**				
GO:0005102	receptor binding	13	512	7.12E-11
GO:0005179	hormone activity	8	114	1.25E-09
GO:0005615	extracellular space	15	1994	5.35E-06
GO:0005515	protein binding	17	3602	3.64E-05
GO:0050880	regulation of blood vessel size	3	21	0.000372955
GO:0051239	regulation of organismal physiological process	5	177	0.000516212
GO:0051216	cartilage development	3	28	0.000580814
GO:0044403	symbiosis, encompassing mutualism through parasitisr	2	4	0.000580814
GO:0044419	interspecies interaction between organisms	2	4	0.000580814
GO:0009405	pathogenesis	2	4	0.000580814
GO:0006874	calcium ion homeostasis	3	35	0.000805894
GO:0019229	regulation of vasoconstriction	2	5	0.000805894
GO:0001501	skeletal development	4	129	0.001492868
GO:0006940	regulation of smooth muscle contraction	2	8	0.0018857
GO:0008015	circulation	3	51	0.0018857
GO:0008083	growth factor activity	4	146	0.001964728
GO:0042592	homeostasis	4	160	0.002536032
GO:0007204	elevation of cytosolic calcium ion concentration	2	11	0.002536032
GO:0051480	cytosolic calcium ion homeostasis	2	11	0.002536032
GO:0042310	vasoconstriction	2	11	0.002536032
GO:0030005	di-, tri-valent inorganic cation homeostasis	3	63	0.002536032
GO:0006875	metal ion homeostasis	3	68	0.002934435
GO:0030003	cation homeostasis	3	70	0.002934435
GO:0006939	smooth muscle contraction	2	13	0.002934435
GO:0007218	neuropeptide signaling pathway	3	71	0.002934435
GO:0030154	cell differentiation	7	799	0.002934435
GO:0006873	cell ion homeostasis	3	74	0.00318259
GO:0050801	ion homeostasis	3	88	0.005118803
GO:0005184	neuropeptide hormone activity	2	19	0.005619797
GO:0019725	cell homeostasis	3	93	0.005619797
GO:0007267	cell-cell signaling	4	230	0.005790154
GO:0046850	regulation of bone remodeling	2	22	0.006865497
GO:0006937	regulation of muscle contraction	2	24	0.007938764
GO:0009190	cyclic nucleotide biosynthesis	2	25	0.008367082
