# Supplementary material for: New genes in the evolution of the neural crest differentiation program
Source: Genome Biol. 2007 Mar 12;8(3):R36. doi: 10.1186/gb-2007-8-3-r36 (PMC1868935; doi:10.1186/gb-2007-8-3-r36)
Supplement: Additional data file 4 — ClustalX alignment of mouse neurotropins against the echinoderm peptide. The comparison reveals a limited amino acid identity. [file gb-2007-8-3-r36-S4.pdf]

```
Mouse_Ngfb/1-307                    1 -------MLCLKPVKLGSLEVGHGQHGGVLACGRAVQGAGWHAGPKLTSVSGPNKGFAKD 53
Mouse_Ntf3/1-258                    1 -------MSILFYVIF-------------LAYLRGIQGN-----------SMDQRSLPED 29
Mouse_Bdnf/1-249                    1 -------MTILFLTMV-------------ISYFGCMKAA-------------PMKEVNVH 27
Mouse_Ntf5/1-209                    1 -------MLPRHSCSL-------------LLFLFLLPSV-------------PMEP---- 23
Sea_Urchin_AAGJ01195706.1/1-204     1 -----------------------------MRMNAN------------------------- 6
Mouse_Tgfb1/1-390                   1 MPPSGLRLLPLLLPLPWLLVLTPGRPAAGLSTCKTIDMELVKRKRIEAIRGQILSKLRLA 60

Mouse_Ngfb/1-307                   54 AAFYTGRSEVHSVMSMLFYTLITAFLIGVQAEPYTDSNVPEGDSVPEAHWTKLQHSLDTA 113
Mouse_Ntf3/1-258                   30 -----------SLNSLIIKLIQADILKNKLSKQMVDVKENYQSTLPKAEAPR-----EPE 73
Mouse_Bdnf/1-249                   28 -----------GQGNLAYPGVRTHGTLESVNGPRAGSRGLTTTSLADTFEHVIEELLDED 76
Mouse_Ntf5/1-209                   24 -----------------HP---PSSTLPPFLAP--------------------------- 36
Sea_Urchin_AAGJ01195706.1/1-204     7 ----------------AVPSPLDSVNMMPGVFP--------------------------- 23
Mouse_Tgfb1/1-390                  61 SP---------PSQGEVPPGPLPEAVLALYNSTRDRVAGESADPEPEPEADYYAKEVTRV 111

Mouse_Ngfb/1-307                  114 LRRARSAPTAPIAARVTGQTRNITVDPRLFKKRRLHSPRVLFSTQPPPTSSD----TLDL 169
Mouse_Ntf3/1-258                   74 QGEATRSEFQPMIATDTELLR---------QQRRYNSPRVLLSDSTPLEPPP----LYLM 120
Mouse_Bdnf/1-249                   77 QKVRPNEENH--------------------KDADLYTSRVMLSSQVPLEPPL----LFLL 112
Mouse_Ntf5/1-209                   37 -------------------------------EWDLLSPRVALSRGAPAGPPL----LFLL 61
Sea_Urchin_AAGJ01195706.1/1-204    24 ---------------------------------GYMSQRVVFSAERPQTPPWPGDHSFKP 50
Mouse_Tgfb1/1-390                 112 LMVDRNNAIYEKTKDIS--------------HSIYMFFNTSDIREAVPEPPLLSRAELRL 157

Mouse_Ngfb/1-307                  170 DFQAHGTIPFNRTHRSKR--SSTHPVFHMGEFS---------VCDSVSVWVG-------- 210
Mouse_Ntf3/1-258                  121 EDYVGNPVVANRTSPRRK--RYAEHKSHRGEYS---------VCDSESLWVT-------- 161
Mouse_Bdnf/1-249                  113 EE--YKNYLDA-ANMSMR-VRRHSDPARRGELS---------VCDSISEWVTAA------ 153
Mouse_Ntf5/1-209                   62 EAGAYGEPAGAPANRSRRGVSETAPASRRGELA---------VCDAVSGWVT-------- 104
Sea_Urchin_AAGJ01195706.1/1-204    51 EAFDEEGDSANKTDDSEVAHSRRRRSSSVSDLEE--------VCESTSGWIV-------- 94
Mouse_Tgfb1/1-390                 158 QRLKSSVEQHVELYQKYSNNSWRYLGNRLLTPTDTPEWLSFDVTGVVRQWLNQGDGIQGF 217

Mouse_Ngfb/1-307                  211 -----------DKTTATDIKGKEVTVLAEVNINNSV--FRQYFFETKCRASNPVESG--- 254
Mouse_Ntf3/1-258                  162 -----------DKSSAIDIRGHQVTVLGEIKTGNSP--VKQYFYETRCKEARPVKNG--- 205
Mouse_Bdnf/1-249                  154 -----------DKKTAVDMSGGTVTVLEKVPVSKGQ--LKQYFYETKCNPMGYTKEG--- 197
Mouse_Ntf5/1-209                  105 -----------DRRTAVDLRGREVEVLGEVPAAGGSP-LRQYFFETRCKAESAGEGGPGV 152
Sea_Urchin_AAGJ01195706.1/1-204    95 ------------KKWGTDMYGQNVTILSEIMTAGNIQ-VTQWFYETACARPQGLHGVQR- 140
Mouse_Tgfb1/1-390                 218 RFSAHCSCDSKDNKLHVEINGISPKRRGDLGTIHDMNRPFLLLMATPLERAQHLHSSR-- 275

Mouse_Ngfb/1-307                  255 ----CRGIDSKHWNSYCTTTHTFVKALTTDE-KQAAWRFIR------------------- 290
Mouse_Ntf3/1-258                  206 ----CRGIDDKHWNSQCKTSQTYVRALTSENNKLVGWRWIR------------------- 242
Mouse_Bdnf/1-249                  198 ----CRGIDKRHWNSQCRTTQSYVRALTMDSKKRIGWRFIR------------------- 234
Mouse_Ntf5/1-209                  153 GGGGCRGVDRRHWLSECKAKQSYVRALTADSQGRVGWRWIR------------------- 193
Sea_Urchin_AAGJ01195706.1/1-204   141 ----CLGIDNNNYDSVCLTKSAWVYAMIRTARGEEGWTWIA------------------- 177
Mouse_Tgfb1/1-390                 276 ----HRRALDTNYCFSSTEKNCCVRQLYIDFRKDLGWKWIHEPKGYHANFCLGPCPYIWS 331
```

```
Mouse_Ngfb/1-307                   291 -----------------IDTACVCVLSRK---------ATRRG----------------  307
Mouse_Ntf3/1-258                   243 -----------------IDTSCVCALSRK---------IGRT-----------------  258
Mouse_Bdnf/1-249                   235 -----------------IDTSCVCTLTIK---------RGR------------------  249
Mouse_Ntf5/1-209                   194 -----------------IDTACVCTLLSR---------TGRA-----------------  209
Sea_Urchin_AAGJ01195706.1/1-204    178 -----------------ISSSCNCAVRQLSL----LEQIGRRSRLTRL-----------  204
Mouse_Tgfb1/1-390                  332 LDTQYSKVLALYNQHNPGASASPCCVPQALEPLPIVYYVGRKPKVEQLSNMIVRSCKCS  390
```
