# Supplementary material for: New genes in the evolution of the neural crest differentiation program
Source: Genome Biol. 2007 Mar 12;8(3):R36. doi: 10.1186/gb-2007-8-3-r36 (PMC1868935; doi:10.1186/gb-2007-8-3-r36)
Supplement: Additional data file 5 — Phylogenetic analysis of neural crest Pfam domains emergence through evolution. The table shows a full list of the compiled 615 genes involved in neural crest development and the first appearance of their Pfam domains in the different clades. All the corresponding Pfam domains of these genes, when these domains have appeared, and the classification of the genes according to our previous sequential blast analysis (blast; color-coded) are indicated. [file gb-2007-8-3-r36-S5.pdf]

gene id	name	blast	pro	euk	met	deu	cor	ver	mam
ENSMUSG00000028933	Xrcc2	pro	-	-	-	-	-	-	-
ENSMUSG00000037313	Tacc3	pro	-	-		-	-	-	-
ENSMUSG00000029286	Enam	pro	-	-	-	-	-	-	-
ENSMUSG00000030450	p	pro	-	-	-	-	-	-	-
ENSMUSG00000051835	Gli2	pro	-	-	-	-	-	-	-
ENSMUSG00000053268	Dspp	pro	-	-	-	-	-	-	-
ENSMUSG00000017756	Slc12a7	pro	AA_permease	-	-	-	-	-	-
ENSMUSG00000024597	Slc12a2	pro	AA_permease	-	-	-	-	-	-
ENSMUSG00000027130	Slc12a6	pro	AA_permease	-	-	-	-	KCl_Cotrans_1	-
ENSMUSG00000028125	Abca4	pro	ABC_tran	-	-	-	-	-	-
ENSMUSG00000041301	Cftr	pro	ABC_tran ABC_me	-	-	-	-	-	-
ENSMUSG00000031378	Abcd1	pro	ABC_tran ABC_me	-	-	-	-	-	-
ENSMUSG00000051855	Mest	pro	Abhydrolase_1	-	-	-	-	-	-
ENSMUSG00000005125	Ndrg1	pro	Abhydrolase_1 Nd	-	-	-	-	-	-
ENSMUSG00000002103	Acp2	pro	Acid_phosphat_A	-	-	-	-	-	-
ENSMUSG00000031985	Gnpat	pro	Acyltransferase	-	-	-	-	-	-
ENSMUSG00000017697	Ada	pro	A_deaminase	-	-	-	-	-	-
ENSMUSG00000013584	Aldh1a2	pro	Aldedh	-	-	-	-	-	-
ENSMUSG00000028766	Akp2	pro	Alk_phosphatase	-	-	-	-	-	-
ENSMUSG00000034450	Gulo	pro	ALO FAD_binding_	-	-	-	-	-	-
ENSMUSG00000024131	Slc3a1	pro	Alpha-amylase	-	-	-	-	-	-
ENSMUSG00000051980	Casr	pro	ANF_receptor	-	7tm_3 NCD3G	-	-	-	-
ENSMUSG00000023947	Nfkbie	pro	Ank	-	-	-	-	-	-
ENSMUSG00000031543	Ank1	pro	Ank	-	Death ZU5	-	-	-	-
ENSMUSG00000022265	Ank	pro	ANKH	-	-	-	-	-	-
ENSMUSG00000005251	Ripk4	pro	Ank Pkinase_Tyr P	-	-	-	-	-	-
ENSMUSG00000005871	Apc	pro	-	Arm	APC_crr APC_15aa	-	-	EB1_binding APC_	-
ENSMUSG00000028207	Asph	pro	Asp_Arg_Hydrox	-	-	-	-	Asp-B-Hydro_N	-
ENSMUSG00000020774	Aspa	pro	AstE_AspA	-	-	-	-	-	-
ENSMUSG00000024899	Papss2	pro	ATP-sulfurylase AP	-	-	-	-	-	-
ENSMUSG00000007038	Neu1	pro	BNR	-	-	-	-	-	-
ENSMUSG00000017146	Brca1	pro	BRCT	zf-C3HC4	-	-	-	-	-
ENSMUSG00000022521	Crebbp	pro	-	Bromodomain	zf-TAZ DUF906 DU	-	-	-	-
ENSMUSG00000032252	Glce	pro	C5-epim_C	-	-	-	-	-	-
ENSMUSG00000052613	Pcdh15	pro	Cadherin	-	-	-	-	-	-
ENSMUSG00000027562	Car2	pro	Carb_anhydrase	-	-	-	-	-	-
ENSMUSG00000021919	Chat	pro	Carn_acyltransf	-	-	-	-	-	-
ENSMUSG00000021629	Slc30a5	pro	Cation_efflux	-	-	-	-	-	-
ENSMUSG00000004317	Clcn5	pro	CBS Voltage_CLC	-	-	-	-	-	-
ENSMUSG00000004319	Clcn3	pro	CBS Voltage_CLC	-	-	-	-	-	-

ENSMUSG00000036636	Clcn7	pro	CBS Voltage_CLC	-	-	-	-	-	-
ENSMUSG00000026114	Cnga3	pro	cNMP_binding Ion_	-	-	-	-	-	-
ENSMUSG00000031789	Cngb1b	pro	cNMP_binding Ion_	-	-	-	-	-	-
ENSMUSG00000026147	Col9a1	pro	Collagen	-	-	-	-	-	-
ENSMUSG00000001506	Col1a1	pro	Collagen	-	COLFI VWC	-	-	-	-
ENSMUSG00000022483	Col2a1	pro	Collagen	-	COLFI VWC	-	-	-	-
ENSMUSG00000027966	Q8VIA3_MOUS	pro	Collagen Laminin_	-	-	-	-	-	-
ENSMUSG00000031174	Rpgr	pro	COX2_TM	RCC1	Sushi	-	-	-	-
ENSMUSG00000028639	Nsep1	pro	CSD	-	-	-	-	-	-
ENSMUSG00000030400	Ercc2	pro	DEAD_2	DUF1227	-	-	-	-	-
ENSMUSG00000034593	Myo5a	pro	-	DIL IQ Myosin_hea	-	-	-	-	-
ENSMUSG00000038268	Dph2l1	pro	Diphthamide_syn	-	-	-	-	-	-
ENSMUSG00000029307	Dmp1	pro	-	-	-	-	-	-	DMP1
ENSMUSG00000017485	Top2b	pro	DNA_topoisoIV DN	-	-	-	-	DTHCT	-
ENSMUSG00000026532	Spna1	pro	efhand Spectrin SH	-	-	-	-	-	-
ENSMUSG00000022098	Bmp1	pro	-	EGF	CUB EGF_CA Astac	-	-	-	-
ENSMUSG00000024913	Lrp5	pro	-	EGF	Ldl_recept_a Ldl_r	-	-	-	-
ENSMUSG00000030201	Lrp6	pro	-	EGF	Ldl_recept_a Ldl_r	-	-	-	-
ENSMUSG00000031906	Smpd3	pro	Exo_endo_phos	-	-	-	-	-	-
ENSMUSG00000031755	Bbs2	pro	FG-GAP	-	-	-	-	-	-
ENSMUSG00000027087	Itgav	pro	FG-GAP	-	Integrin_alpha	-	-	-	-
ENSMUSG00000064201	Krt2-17	pro	-	-	Filament	-	-	-	-
ENSMUSG00000022054	Nef3	pro	-	-	Filament	-	-	Filament_head	-
ENSMUSG00000022055	Nefl	pro	-	-	Filament	-	-	Filament_head	-
ENSMUSG00000035916	Ptprq	pro	fn3	-	-	-	-	-	-
ENSMUSG00000051747	Ttn	pro	fn3 I-set	-	ig V-set	-	-	-	-
ENSMUSG00000060534	Dcc	pro	fn3 I-set	-	Neogenin_C ig V-s	-	-	-	-
ENSMUSG00000028289	Epha7	pro	fn3 Pkinase_Tyr PI	SAM_1 SAM_2	Ephrin_lbd	-	-	-	-
ENSMUSG00000037514	Pank2	pro	Fumble	-	-	-	-	-	-
ENSMUSG00000029491	Pde6b	pro	GAF	PDEase_I	-	-	-	-	-
ENSMUSG00000027523	Gnas	pro	-	G-alpha	-	-	-	-	NESP55
ENSMUSG00000006345	Ggt1	pro	G_glu_transpept	-	-	-	-	-	-
ENSMUSG00000058488	Kl	pro	Glyco_hydro_1	-	-	-	-	-	-
ENSMUSG00000021665	Hexb	pro	Glyco_hydro_20 G	-	-	-	-	-	-
ENSMUSG00000025232	Hexa	pro	Glyco_hydro_20 G	-	-	-	-	-	-
ENSMUSG00000025534	BGLR_MOUSE	pro	Glyco_hydro_2_C	-	-	-	-	-	-
ENSMUSG00000028048	Gba	pro	Glyco_hydro_30	-	-	-	-	-	-
ENSMUSG00000025579	Gaa	pro	Glyco_hydro_31	-	Trefoil	-	-	-	-
ENSMUSG00000032348	Gsta4	pro	GST_C GST_N	-	-	-	-	-	-
ENSMUSG00000028469	Npr2	pro	Guanylate_cyc Pki	-	-	-	-	-	-
ENSMUSG00000022770	Dlgh1	pro	Guanylate_kin SH.	-	-	-	-	-	-

ENSMUSG00000025982	Sf3b1	pro	HEAT	-	-	-	-	-	-
ENSMUSG00000033762	NP_478121.1	pro	Helicase_C DEAD	zf-CCHC	-	-	-	-	-
ENSMUSG00000029920	Smarcad1	pro	Helicase_C SNF2_	-	-	-	-	-	-
ENSMUSG00000024921	Smarca2	pro	Helicase_C SNF2_	Bromodomain HSA	TCH	-	-	-	-
ENSMUSG00000026313	Hdac4	pro	Hist_deacetyl	-	-	-	-	-	-
ENSMUSG00000024241	Sos1	pro	Histone	RasGEF RasGEF_N	-	-	-	-	-
ENSMUSG00000032060	Cryab	pro	HSP20	-	-	-	-	Crystallin	-
ENSMUSG00000033792	Atp7a	pro	Hydrolase E1-E2_A	-	-	-	-	-	-
ENSMUSG00000024590	Lmnb1	pro	-	-	IF_tail Filament	-	-	-	-
ENSMUSG00000028063	Lmna	pro	-	-	IF_tail Filament	-	-	-	-
ENSMUSG00000039959	Hip1	pro	-	I_LWEQ ANTH EN	-	-	-	-	-
ENSMUSG00000000915	Hip1r	pro	-	I_LWEQ ENTH AN	-	-	-	-	-
ENSMUSG00000026407	Cacna1s	pro	Ion_trans	-	-	-	-	-	-
ENSMUSG00000009545	Kcnq1	pro	Ion_trans_2 Ion_t	-	KCNQ_channel	-	-	-	-
ENSMUSG00000023033	Scn8a	pro	Ion_trans	IQ	Na_trans_assoc	-	-	-	-
ENSMUSG00000030592	Ryr1	pro	Ion_trans	SPRY MIR	RR_TM4-6 RyR RY	-	-	-	-
ENSMUSG00000033196	NP_659210.1	pro	-	IQ Myosin_head M	Myosin_tail_1	-	-	-	-
ENSMUSG00000056328	Myh1	pro	-	IQ Myosin_head M	Myosin_tail_1	-	-	-	-
ENSMUSG00000041695	Kcnj2	pro	IRK	-	-	-	-	-	-
ENSMUSG00000027312	Atrn	pro	Kelch_1 Kelch_2	EGF EGF_2	Laminin_EGF PSI I	-	-	-	-
ENSMUSG00000016028	Celsr1	pro	Laminin_G_2 Cadh	EGF	7tm_2 GPS HRM L	-	-	-	-
ENSMUSG00000028763	Hspg2	pro	Laminin_G_2 I-set	EGF_2 EGF	Laminin_G_1 ig V-	-	-	-	-
ENSMUSG00000015647	Lama5	pro	Laminin_G_2	-	Laminin_EGF Lam	-	-	-	-
ENSMUSG00000024421	Lama3	pro	Laminin_G_2	-	Laminin_G_1 Lam	-	-	-	-
ENSMUSG00000039233	Tbce	pro	LRR_1	CAP_GLY	-	-	-	-	-
ENSMUSG00000040472	Rabggta	pro	LRR_1	LRR_2 PPTA	-	-	-	RabGGT_insert	-
ENSMUSG00000019929	Dcn	pro	LRR_1	-	LRRNT	-	-	-	-
ENSMUSG00000031375	Bgn	pro	LRR_1	-	LRRNT	-	-	-	-
ENSMUSG00000036446	Lum	pro	LRR_1	-	LRRNT	-	-	-	-
ENSMUSG00000041559	Fmod	pro	LRR_1	-	LRRNT	-	-	-	-
ENSMUSG00000037375	Hhat	pro	MBOAT	-	-	-	-	-	-
ENSMUSG00000001348	Acp5	pro	Metallophos	-	-	-	-	-	-
ENSMUSG00000022243	Matp	pro	MFS_1	-	-	-	-	-	-
ENSMUSG00000032114	Slc37a4	pro	MFS_1	-	-	-	-	-	-
ENSMUSG00000029009	Mthfr	pro	MTHFR	-	-	-	-	-	-
ENSMUSG00000021490	Slc34a1	pro	Na_Pi_cotrans	-	-	-	-	-	-
ENSMUSG00000028003	Lrat	pro	NC	-	-	-	-	-	-
ENSMUSG00000033676	Gabrb3	pro	-	-	Neur_chan_memb	-	-	-	-
ENSMUSG00000006024	Napa	pro	NSF	-	-	-	-	-	-
ENSMUSG00000006724	Cyp27b1	pro	p450	-	-	-	-	-	-
ENSMUSG00000024987	Cyp26a1	pro	p450	-	-	-	-	-	-

ENSMUSG00000032274	Cyp19a1	pro	p450	-	-	-	-	-	-
ENSMUSG00000063415	Cyp26b1	pro	p450	-	-	-	-	-	-
ENSMUSG00000015522	Arnt	pro	PAS	HLH	-	-	-	-	-
ENSMUSG00000021466	Ptch1	pro	Patched	-	-	-	-	-	-
ENSMUSG00000030768	Disp1	pro	Patched	-	-	-	-	-	-
ENSMUSG00000020733	Slc9a3r1	pro	PDZ	-	-	-	-	-	-
ENSMUSG00000039137	Whrn	pro	PDZ	-	-	-	-	-	-
ENSMUSG00000053198	Prx	pro	PDZ	-	-	-	-	-	-
ENSMUSG00000028111	Ctsk	pro	Peptidase_C1	-	-	-	-	-	-
ENSMUSG00000000957	Mmp14	pro	Peptidase_M10	-	Hemopexin	-	-	-	-
ENSMUSG00000018620	Mmp20	pro	Peptidase_M10	-	Hemopexin	-	-	-	-
ENSMUSG00000043613	Mmp3	pro	Peptidase_M10	-	Hemopexin	-	-	-	-
ENSMUSG00000050578	Mmp13	pro	Peptidase_M10	-	Hemopexin	-	-	-	-
ENSMUSG00000031740	Mmp2	pro	Peptidase_M10	-	Hemopexin	fn2	-	-	-
ENSMUSG00000057457	Phex	pro	Peptidase_M13_N	-	-	-	-	-	-
ENSMUSG00000022842	Ece2	pro	Peptidase_M13 Pe	-	-	-	-	-	-
ENSMUSG00000057530	Ece1	pro	Peptidase_M13 Pe	-	-	-	-	-	-
ENSMUSG00000021557	Agtpbp1	pro	Peptidase_M14	-	-	-	-	-	-
ENSMUSG00000000738	Spg7	pro	Peptidase_M41 AA	-	-	-	-	-	-
ENSMUSG00000017760	Ppgb	pro	Peptidase_S10	-	-	-	-	-	-
ENSMUSG00000041534	Rbp3	pro	Peptidase_S41	-	-	-	-	-	-
ENSMUSG00000004508	Gab2	pro	-	PH	-	-	-	-	-
ENSMUSG00000039943	Plcb4	pro	PI-PLC-X	C2 PI-PLC-Y	DUF1154	-	-	-	-
ENSMUSG00000032855	Pkd1	pro	PKD LRR_1	WSC	PKD_channel PLAT	-	-	REJ	-
ENSMUSG00000024122	Pdpk1	pro	Pkinase	-	-	-	-	-	-
ENSMUSG00000025199	Chuk	pro	Pkinase	-	-	-	-	-	-
ENSMUSG00000031537	Ikbkb	pro	Pkinase	-	-	-	-	-	-
ENSMUSG00000000530	Acvrl1	pro	Pkinase	-	Activin_recp	-	-	-	-
ENSMUSG00000052155	Acvr2a	pro	Pkinase	-	Activin_recp	-	-	-	-
ENSMUSG00000061393	Acvr2b	pro	Pkinase	-	Activin_recp	-	-	-	-
ENSMUSG00000029334	Prkg2	pro	Pkinase cNMP_bin	-	-	-	-	-	-
ENSMUSG00000001729	Akt1	pro	Pkinase	Pkinase_C PH	-	-	-	-	-
ENSMUSG00000004056	Akt2	pro	Pkinase	Pkinase_C PH	-	-	-	-	-
ENSMUSG00000031309	Rps6ka3	pro	Pkinase Pkinase_T	Pkinase_C	-	-	-	-	-
ENSMUSG00000031450	Grk1	pro	Pkinase	RGS	-	-	-	-	-
ENSMUSG00000003534	Ddr1	pro	Pkinase_Tyr F5_F8	-	-	-	-	-	-
ENSMUSG00000021796	Bmpr1a	pro	Pkinase_Tyr Pkina	-	Activin_recp	-	-	-	-
ENSMUSG00000052430	Bmpr1b	pro	Pkinase_Tyr Pkina	-	Activin_recp	-	-	-	-
ENSMUSG00000030890	Ilk	pro	Pkinase_Tyr Pkina	-	-	-	-	-	-
ENSMUSG00000001127	Araf	pro	Pkinase_Tyr Pkina	C1_1	RBD	-	-	-	-
ENSMUSG00000030110	Ret	pro	Pkinase_Tyr Pkina	-	-	-	-	-	-

ENSMUSG00000014361	Mertk	pro	Pkinase_Tyr Pkina	-	ig	-	-	-	-
ENSMUSG00000002602	Axl	pro	Pkinase_Tyr Pkina	-	ig V-set	-	-	-	-
ENSMUSG00000027298	Tyro3	pro	Pkinase_Tyr Pkina	-	ig V-set	-	-	-	-
ENSMUSG00000005533	Igf1r	pro	Pkinase_Tyr Pkina	-	Recep_L_domain I	-	-	-	-
ENSMUSG00000005672	Kit	pro	Pkinase_Tyr Pkina	-	ig V-set	-	-	-	-
ENSMUSG00000029231	Pdgfra	pro	Pkinase_Tyr Pkina	-	ig	-	-	-	-
ENSMUSG00000023972	Ptk7	pro	Pkinase_Tyr Pkina	-	ig V-set	-	-	-	-
ENSMUSG00000030849	Fgfr2	pro	Pkinase_Tyr Pkina	-	ig V-set	-	-	-	-
ENSMUSG00000031565	Fgfr1	pro	Pkinase_Tyr Pkina	-	ig V-set	-	-	-	-
ENSMUSG00000055254	Ntrk2	pro	Pkinase_Tyr Pkina	-	ig V-set LRRNT	-	-	-	-
ENSMUSG00000021464	Ror2	pro	Pkinase_Tyr Pkina	Kringle	Fz ig V-set	-	-	-	-
ENSMUSG00000035305	Ror1	pro	Pkinase_Tyr Pkina	Kringle	Fz ig V-set	-	-	-	-
ENSMUSG00000059146	Ntrk3	pro	Pkinase_Tyr Pkina	-	ig LRRNT	-	-	-	-
ENSMUSG00000020700	Map3k3	pro	Pkinase_Tyr Pkina	PB1	-	-	-	-	-
ENSMUSG00000021701	Plk2	pro	Pkinase_Tyr Pkina	POLO_box	-	-	-	-	-
ENSMUSG00000020122	Egfr	pro	Pkinase_Tyr Pkina	-	Recep_L_domain I	-	-	-	-
ENSMUSG00000026117	Zap70	pro	Pkinase_Tyr Pkina	SH2	-	-	-	-	-
ENSMUSG00000014932	Yes1	pro	Pkinase_Tyr Pkina	SH2	-	-	-	-	-
ENSMUSG00000027646	Src	pro	Pkinase_Tyr Pkina	SH2	-	-	-	-	-
ENSMUSG00000032312	Csk	pro	Pkinase_Tyr Pkina	SH2	-	-	-	-	-
ENSMUSG00000032547	Ryk	pro	Pkinase_Tyr Pkina	-	WIF	-	-	-	-
ENSMUSG00000030513	Pcsk6	pro	P_proprotein Pepti	-	-	-	-	-	-
ENSMUSG00000017737	Mmp9	pro	PT Peptidase_M10	-	Hemopexin	fn2	-	-	-
ENSMUSG00000032067	Pts	pro	PTPS	-	-	-	-	-	-
ENSMUSG00000020380	Rad50	pro	Rad50_zn_hook	-	-	-	-	-	-
ENSMUSG00000030559	Rab38	pro	Ras	-	-	-	-	-	-
ENSMUSG00000022601	Rpl24	pro	Ribosomal_L24e	-	-	-	-	-	-
ENSMUSG00000056550	Rpl24	pro	Ribosomal_L24e	-	-	-	-	-	-
ENSMUSG00000028174	Rpe65	pro	RPE65	-	-	-	-	-	-
ENSMUSG00000054256	Msi1h	pro	RRM_1	-	-	-	-	-	-
ENSMUSG00000002028	Mll	pro	SET AT_hook	PHD	FYRC FYRN zf-CX)	-	-	-	-
ENSMUSG00000042678	Myo15	pro	SH3_2	IQ Myosin_head	MyTH4	-	-	-	-
ENSMUSG00000021609	Slc6a3	pro	SNF	-	-	-	-	-	-
ENSMUSG00000030096	Slc6a6	pro	SNF	-	-	-	-	-	-
ENSMUSG00000026131	Dst	pro	Spectrin efhand SI	CH	Plectin	-	-	-	-
ENSMUSG00000020315	Spnb2	pro	Spectrin	PH CH	-	-	-	-	-
ENSMUSG00000019820	Utrn	pro	Spectrin	WW CH	-	-	-	-	-
ENSMUSG00000045103	Dmd	pro	Spectrin	WW CH	-	-	-	-	-
ENSMUSG00000023945	Slc5a7	pro	SSF	-	-	-	-	-	-
ENSMUSG00000020651	Slc26a4	pro	STAS Sulfate_tran	-	-	-	-	-	-
ENSMUSG00000034320	Slc26a2	pro	STAS Sulfate_tran	-	-	-	-	-	-

ENSMUSG00000028832	Stmn1	pro	-	-	Stathmin	-	-	-	-
ENSMUSG00000032018	Sc5d	pro	Sterol_desat	-	-	-	-	-	-
ENSMUSG00000005043	Sgsh	pro	Sulfatase	-	-	-	-	-	-
ENSMUSG00000042093	Arsb	pro	Sulfatase	-	-	-	-	-	-
ENSMUSG00000028370	Pappa	pro	-	-	Sushi	-	-	Peptidase_M43	-
ENSMUSG00000010476	Ebf3	pro	TIG	-	-	-	-	-	-
ENSMUSG00000029765	Plxna4	pro	TIG	-	PSI Sema	-	-	-	-
ENSMUSG00000031398	Plxna3	pro	TIG	-	PSI Sema	-	-	-	-
ENSMUSG00000025235	Bbs4	pro	TPR_1 TPR_2	-	-	-	-	-	-
ENSMUSG00000036918	Ttc7	pro	TPR_2 TPR_1	-	-	-	-	-	-
ENSMUSG00000019428	Fkbp8	pro	TPR_2 TPR_1 FKB	-	-	-	-	-	-
ENSMUSG00000035606	Ky	pro	Transglut_core	-	-	-	-	-	-
ENSMUSG00000021822	Plau	pro	Trypsin	Kringle EGF_2	-	-	-	-	-
ENSMUSG00000031538	Plat	pro	Trypsin	Kringle EGF	-	fn1	-	-	-
ENSMUSG00000023885	Thbs2	pro	TSP_3	EGF TSP_1	TSP_C VWC	-	-	-	-
ENSMUSG00000040152	Thbs1	pro	TSP_3	EGF TSP_1	TSP_C VWC	-	-	-	-
ENSMUSG00000028047	Thbs3	pro	TSP_3	-	TSP_C EGF_CA	-	-	-	-
ENSMUSG00000004651	Tyr	pro	Tyrosinase	-	-	-	-	-	-
ENSMUSG00000005994	Tyrp1	pro	Tyrosinase	-	-	-	-	-	-
ENSMUSG00000022129	Dct	pro	Tyrosinase	-	-	-	-	-	-
ENSMUSG00000001750	Tcirg1	pro	V_ATPase_I	-	-	-	-	-	-
ENSMUSG00000040533	Matn1	pro	VWA	EGF	-	-	-	-	-
ENSMUSG00000020003	Pex7	pro	WD40	-	-	-	-	-	-
ENSMUSG00000021694	Ercc8	pro	WD40	-	-	-	-	-	-
ENSMUSG00000019979	Apaf1	pro	WD40 NB-ARC	-	CARD	-	-	-	-
ENSMUSG00000062312	Erbb2	pro	YLP Pkinase_Tyr P	-	Recep_L_domain I	-	-	-	-
ENSMUSG00000021738	Sca7	euk	-	-	-	-	-	-	-
ENSMUSG00000040084	Bub1b	euk	-	-	-	-	-	-	-
ENSMUSG00000031972	Acta1	euk	-	Actin	-	-	-	-	-
ENSMUSG00000007891	Ctsd	euk	-	Asp	A1_Propeptide	-	-	-	-
ENSMUSG00000019726	Lyst	euk	-	Beach	-	-	-	-	-
ENSMUSG00000024002	Brd4	euk	-	Bromodomain	-	-	-	-	-
ENSMUSG00000052837	Junb	euk	-	bZIP_1 bZIP_2	Jun	-	-	-	-
ENSMUSG00000052684	Jun	euk	-	bZIP_2 bZIP_1	Jun	-	-	-	-
ENSMUSG00000001349	Cnn1	euk	-	CH	Calponin	-	-	-	-
ENSMUSG00000030720	Cln3	euk	-	CLN3	-	-	-	-	-
ENSMUSG00000056201	Cfl1	euk	-	Cofilin_ADF	-	-	-	-	-
ENSMUSG00000031071	Ccnd1	euk	-	Cyclin_C Cyclin_N	-	-	-	-	-
ENSMUSG00000020888	Dvl2	euk	DEP PDZ	-	Dishevelled DIX	-	-	-	-
ENSMUSG00000052593	Adam17	euk	-	Disintegrin Reprol	-	-	-	-	-
ENSMUSG00000039176	Polg	euk	DNA_pol_A	-	-	-	-	-	-

ENSMUSG00000024842	Cabp4	euk	efhand	-	-	-	-	-	-
ENSMUSG00000011256	Adam19	euk	-	EGF_2 Disintegrin	Pep_M12B_proper	-	-	-	-
ENSMUSG00000020689	Itgb3	euk	-	EGF_2 EGF	Integrin_B_tail Int	-	-	-	-
ENSMUSG00000002799	Jag2	euk	-	EGF EGF_2	EGF_CA DSL MNN	-	-	-	-
ENSMUSG00000024940	Ltbp3	euk	-	EGF EGF_2	EGF_CA	TB	-	-	-
ENSMUSG00000003436	Dll3	euk	-	EGF EGF_2	MNNL	-	-	-	-
ENSMUSG00000021186	Fbln5	euk	-	EGF	EGF_CA	-	-	-	-
ENSMUSG00000004821	Brd4	euk	-	EMP24_GP25L	-	-	-	-	-
ENSMUSG00000032737	Inppl1	euk	Exo_endo_phos	SAM_1 SAM_2 SH	-	-	-	-	-
ENSMUSG00000022973	Synj1	euk	Exo_endo_phos	Syja_N	-	-	-	-	-
ENSMUSG00000035996	Ccnf	euk	F-box	Cyclin_C Cyclin_N	-	-	-	-	-
ENSMUSG00000063077	Kif1b	euk	FHA	PH Kinesin	-	-	-	-	-
ENSMUSG00000029245	Q8C278_MOU	euk	fn3	-	Ephrin_lbd	-	-	-	-
ENSMUSG00000002057	Foxn1	euk	-	Fork_head	-	-	-	-	-
ENSMUSG00000020950	Foxg1	euk	-	Fork_head	-	-	-	-	-
ENSMUSG00000037025	Foxa2	euk	-	Fork_head	-	-	-	-	-
ENSMUSG00000038402	Foxf2	euk	-	Fork_head	-	-	-	-	-
ENSMUSG00000042002	Foxn4	euk	-	Fork_head	-	-	-	-	-
ENSMUSG00000046714	Foxc2	euk	-	Fork_head	-	-	-	-	-
ENSMUSG00000047861	Foxi1	euk	-	Fork_head	-	-	-	-	-
ENSMUSG00000050295	Foxc1	euk	-	Fork_head	-	-	-	-	-
ENSMUSG00000024639	Gnaq	euk	-	G-alpha	-	-	-	-	-
ENSMUSG00000034781	Gna11	euk	-	G-alpha	-	-	-	-	-
ENSMUSG00000015619	Gata3	euk	-	GATA	-	-	-	-	-
ENSMUSG00000025531	Chm	euk	GDI	-	-	-	-	-	-
ENSMUSG00000021733	Slc4a7	euk	-	HCO3_cotransp	Band_3_cyto	-	-	-	-
ENSMUSG00000028962	Slc4a2	euk	-	HCO3_cotransp	Band_3_cyto	-	-	-	-
ENSMUSG00000021686	Ap3b1	euk	HEAT	Adaptin_N	-	-	-	-	-
ENSMUSG00000020198	Ap3d1	euk	HEAT	Adaptin_N	-	-	-	BLVR	-
ENSMUSG00000006932	Ctnnb1	euk	HEAT	Arm	-	-	-	-	-
ENSMUSG00000038780	Smurf1	euk	-	HECT WW C2	-	-	-	-	-
ENSMUSG00000000134	Tcfe3	euk	-	HLH	-	-	-	-	-
ENSMUSG00000035158	Mitf	euk	-	HLH	-	-	-	-	-
ENSMUSG00000018585	Atox1	euk	HMA	-	-	-	-	-	-
ENSMUSG00000000567	Sox9	euk	-	HMG_box	-	-	-	-	-
ENSMUSG00000003923	Tfam	euk	-	HMG_box	-	-	-	-	-
ENSMUSG00000024176	Sox8	euk	-	HMG_box	-	-	-	-	-
ENSMUSG00000027985	Lef1	euk	-	HMG_box	-	-	-	-	-
ENSMUSG00000033006	Sox10	euk	-	HMG_box	-	-	-	-	-
ENSMUSG00000041540	Sox5	euk	-	HMG_box	-	-	-	-	-
ENSMUSG00000045179	Sox3	euk	-	HMG_box	-	-	-	-	-

ENSMUSG00000046470	Sox18	euk	-	HMG_box	-	-	-	-	-
ENSMUSG00000000938	Hoxa10	euk	-	Homeobox	-	-	-	-	-
ENSMUSG00000001493	Meox1	euk	-	Homeobox	-	-	-	-	-
ENSMUSG00000001657	Hoxc8	euk	-	Homeobox	-	-	-	-	-
ENSMUSG00000001815	Evx2	euk	-	Homeobox	-	-	-	-	-
ENSMUSG00000001823	Hoxd12	euk	-	Homeobox	-	-	-	-	-
ENSMUSG00000021469	Msx2	euk	-	Homeobox	-	-	-	-	-
ENSMUSG00000029546	Uncx4.1	euk	-	Homeobox	-	-	-	-	-
ENSMUSG00000029754	Dlx6	euk	-	Homeobox	-	-	-	-	-
ENSMUSG00000029755	Dlx5	euk	-	Homeobox	-	-	-	-	-
ENSMUSG00000038210	Hoxa11	euk	-	Homeobox	-	-	-	-	-
ENSMUSG00000038721	Hoxb7	euk	-	Homeobox	-	-	-	-	-
ENSMUSG00000038805	Six3	euk	-	Homeobox	-	-	-	-	-
ENSMUSG00000040726	Hesx1	euk	-	Homeobox	-	-	-	-	-
ENSMUSG00000041730	Prrxl1	euk	-	Homeobox	-	-	-	-	-
ENSMUSG00000042499	Hoxd11	euk	-	Homeobox	-	-	-	-	-
ENSMUSG00000043969	Emx2	euk	-	Homeobox	-	-	-	-	-
ENSMUSG00000048450	Msx1	euk	-	Homeobox	-	-	-	-	-
ENSMUSG00000050368	HXD10_MOUS	euk	-	Homeobox	-	-	-	-	-
ENSMUSG00000051367	Six1	euk	-	Homeobox	-	-	-	-	-
ENSMUSG00000056648	Hoxb8	euk	-	Homeobox	-	-	-	-	-
ENSMUSG00000059723	2700086A05R	euk	-	Homeobox	-	-	-	-	-
ENSMUSG00000018698	Lhx1	euk	-	Homeobox LIM	-	-	-	-	-
ENSMUSG00000026686	Lmx1a	euk	-	Homeobox LIM	-	-	-	-	-
ENSMUSG00000028201	Lhx8	euk	-	Homeobox LIM	-	-	-	-	-
ENSMUSG00000038765	Lmx1b	euk	-	Homeobox LIM	-	-	-	-	-
ENSMUSG00000024518	Rax	euk	-	Homeobox	OAR	-	-	-	-
ENSMUSG00000025229	Pitx3	euk	-	Homeobox	OAR	-	-	-	-
ENSMUSG00000036602	Cart1	euk	-	Homeobox	OAR	-	-	-	-
ENSMUSG00000040310	Alx4	euk	-	Homeobox	OAR	-	-	-	-
ENSMUSG00000004872	Pax3	euk	-	Homeobox	PAX	-	-	-	-
ENSMUSG00000027168	Pax6	euk	-	Homeobox	PAX	-	-	-	-
ENSMUSG00000028736	Pax7	euk	-	Homeobox	PAX	-	-	-	-
ENSMUSG00000052534	Pbx1	euk	-	Homeobox	PBC	-	-	-	-
ENSMUSG00000031688	Pou4f-rs1	euk	-	Homeobox	Pou	-	-	-	-
ENSMUSG00000048349	Pou4f1	euk	-	Homeobox	Pou	-	-	-	-
ENSMUSG00000056854	Pou3f4	euk	-	Homeobox	Pou	-	-	-	-
ENSMUSG00000005917	Otx1	euk	-	Homeobox	-	-	-	TF_Otx	-
ENSMUSG00000021848	Otx2	euk	-	Homeobox	-	-	-	TF_Otx	-
ENSMUSG00000063681	Crb1	euk	Laminin_G_2	EGF EGF_2	EGF_CA	-	-	-	-
ENSMUSG00000028266	Lmo4	euk	-	LIM	-	-	-	-	-

ENSMUSG00000021767	Myst4	euk	-	MOZ_SAS PHD Lin	-	-	-	-	-	
ENSMUSG00000031918	Mtmr2	euk	-	Myotub-related GF	-	-	-	-	-	
ENSMUSG00000028657	Ppt1	euk	-	Palm_thioest	-	-	-	-	-	
ENSMUSG00000029223	Uchl1	euk	-	Peptidase_C12	-	-	-	-	-	
ENSMUSG00000018547	Pip5k2b	euk	-	PIP5K	-	-	-	-	-	
ENSMUSG00000005949	Ctns	euk	-	PQ-loop	-	-	-	-	-	
ENSMUSG00000020716	Nf1	euk	-	RasGAP	-	-	-	-	-	
ENSMUSG00000026797	Stxbp1	euk	-	Sec1	-	-	-	-	-	
ENSMUSG00000029434	Vps33a	euk	-	Sec1	-	-	-	-	-	
ENSMUSG00000030761	Myo7a	euk	SH3_1	RA IQ Myosin_hea	MyTH4	-	-	-	-	
ENSMUSG00000034330	Plcg2	euk	SH3_2 SH3_1 PI-I	C2 PI-PLC-Y SH2	-	-	-	-	-	
ENSMUSG00000006134	Crkl	euk	SH3_2 SH3_1	SH2	-	-	-	-	-	
ENSMUSG00000059923	Grb2	euk	SH3_2 SH3_1	SH2	-	-	-	-	-	
ENSMUSG00000021549	Rasa1	euk	SH3_2 SH3_1	SH2 RasGAP C2 PI	-	-	-	-	-	
ENSMUSG00000015605	Srf	euk	-	SRF-TF	-	-	-	-	-	
ENSMUSG00000040554	Aipl1	euk	TPR_2 TPR_1 FKB	-	-	-	-	-	-	
ENSMUSG00000001521	Tulp3	euk	-	Tub	-	-	-	-	-	
ENSMUSG00000028426	Rad23b	euk	UBA ubiquitin	-	-	-	-	-	-	
ENSMUSG00000020583	Matn3	euk	VWA	EGF	-	-	-	-	-	
ENSMUSG00000022324	Matn2	euk	VWA	EGF	EGF_CA	-	-	-	-	
ENSMUSG00000031165	Was	euk	-	WH2 PBD WH1	-	-	-	-	-	
ENSMUSG00000028329	Xpa	euk	-	XPA_C XPA_N	-	-	-	-	-	
ENSMUSG00000028496	Mllt3	euk	-	YEATS	-	-	-	-	-	
ENSMUSG00000041836	Ptpre	euk	Y_phosphatase	-	-	-	-	-	-	
ENSMUSG00000043733	Ptpn11	euk	Y_phosphatase	SH2	-	-	-	-	-	
ENSMUSG00000021318	Gli3	euk	zf-C2H2	-	-	-	-	-	-	
ENSMUSG00000022330	Osr2	euk	zf-C2H2	-	-	-	-	-	-	
ENSMUSG00000022676	Snai2	euk	zf-C2H2	-	-	-	-	-	-	
ENSMUSG00000024565	Sall3	euk	zf-C2H2	-	-	-	-	-	-	
ENSMUSG00000025407	Gli1	euk	zf-C2H2	-	-	-	-	-	-	
ENSMUSG00000025959	Klf7	euk	zf-C2H2	-	-	-	-	-	-	
ENSMUSG00000027109	Sp3	euk	zf-C2H2	-	-	-	-	-	-	
ENSMUSG00000032368	Zic1	euk	zf-C2H2	-	-	-	-	-	-	
ENSMUSG00000037465	Klf10	euk	zf-C2H2	-	-	-	-	-	-	
ENSMUSG00000037868	Egr2	euk	zf-C2H2	-	-	-	-	-	-	
ENSMUSG00000048562	Sp8	euk	zf-C2H2	-	-	-	-	-	-	
ENSMUSG00000060284	Sp7	euk	zf-C2H2	-	-	-	-	-	-	
ENSMUSG00000043099	Hic1	euk	zf-C2H2	BTB	-	-	-	-	-	
ENSMUSG00000027104	Atf2	euk	zf-C2H2	bZIP_2 bZIP_1	-	-	-	-	-	
ENSMUSG00000057409	Zfp53	euk	zf-C2H2	-	-	-	-	-	KRAB	
ENSMUSG00000030380	Zfp98	euk	zf-C2H2	-	-	-	-	SCAN	-	

ENSMUSG00000018537	Pcgf2	euk	-	zf-C3HC4	-	-	-	-	-
ENSMUSG00000022517	Mgrn1	euk	-	zf-C3HC4	-	-	-	-	-
ENSMUSG00000024325	Ring1	euk	-	zf-C3HC4	-	-	-	-	-
ENSMUSG00000024807	Syvn1	euk	-	zf-C3HC4	-	-	-	-	-
ENSMUSG00000026484	Rnf2	euk	-	zf-C3HC4	-	-	-	-	-
ENSMUSG00000026739	Bmi1	euk	-	zf-C3HC4	-	-	-	-	-
ENSMUSG00000027164	Traf6	euk	-	zf-C3HC4 MATH	zf-TRAF	-	-	-	-
ENSMUSG00000040374	Pxmp3	euk	-	zf-C3HC4 Pex2_Pe	-	-	-	-	-
ENSMUSG00000044786	Zfp36	euk	-	zf-CCCH	-	-	-	-	-
ENSMUSG00000058886	Deaf1	euk	-	zf-MYND	SAND	-	-	-	-
ENSMUSG00000009207	Lnp	met	-	-	-	-	-	-	-
ENSMUSG00000014418	Hps5	met	-	-	-	-	-	-	-
ENSMUSG00000024302	Dtna	met	-	-	-	-	-	-	-
ENSMUSG00000026303	Mlph	met	-	-	-	-	-	-	-
ENSMUSG00000027615	Hps3	met	-	-	-	-	-	-	-
ENSMUSG00000038280	Ostm1	met	-	-	-	-	-	-	-
ENSMUSG00000041309	Nkx6-2	met	-	-	-	-	-	-	-
ENSMUSG00000060708	Cno	met	-	-	-	-	-	-	-
ENSMUSG00000062115	Rai1	met	-	-	-	-	-	-	-
ENSMUSG00000022122	Ednrb	met	-	-	7tm_1	-	-	-	-
ENSMUSG00000026180	Il8rb	met	-	-	7tm_1	-	-	-	-
ENSMUSG00000030324	Rho	met	-	-	7tm_1	-	-	-	-
ENSMUSG00000031616	Ednra	met	-	-	7tm_1	-	-	-	-
ENSMUSG00000032259	Drd2	met	-	-	7tm_1	-	-	-	-
ENSMUSG00000035283	Adrb1	met	-	-	7tm_1	-	-	-	-
ENSMUSG00000038668	Edg2	met	-	-	7tm_1	-	-	-	-
ENSMUSG00000039358	Drd5	met	-	-	7tm_1	-	-	-	-
ENSMUSG00000045730	Adrb2	met	-	-	7tm_1	-	-	-	-
ENSMUSG00000047259	Mc4r	met	-	-	7tm_1	-	-	-	-
ENSMUSG00000050164	Gpr24	met	-	-	7tm_1	-	-	-	-
ENSMUSG00000023964	Calcr	met	-	-	7tm_2 HRM	-	-	-	-
ENSMUSG00000021814	Anxa7	met	-	-	Annexin	-	-	-	-
ENSMUSG00000025577	Cbx2	met	AT_hook	Chromo	-	-	-	-	-
ENSMUSG00000003873	Bax	met	-	-	Bcl-2	-	-	-	-
ENSMUSG00000057329	Bcl2	met	-	-	Bcl-2 BH4	-	-	-	-
ENSMUSG00000055024	Ep300	met	-	Bromodomain	zf-TAZ DUF906 DU	-	-	-	-
ENSMUSG00000020423	Btg2	met	-	-	BTG	-	-	-	-
ENSMUSG00000037573	Tob1	met	-	-	BTG	-	-	-	-
ENSMUSG00000052435	Cebpe	met	-	bZIP_2 bZIP_1	-	-	-	-	-
ENSMUSG00000040632	Nrl	met	-	-	bZIP_Maf	-	-	-	-
ENSMUSG00000055435	Maf	met	-	-	bZIP_Maf	-	-	-	-

ENSMUSG00000004581	Rpgrip1	met	-	C2	-	-	-	-	-
ENSMUSG00000031885	Cbfb	met	-	-	CBF_beta	-	-	-	-
ENSMUSG00000003031	Cdkn1b	met	-	-	CDI	-	-	-	-
ENSMUSG00000037664	Cdkn1c	met	-	-	CDI	-	-	-	-
ENSMUSG00000006958	Chrd	met	CHRD	-	VWC	-	-	-	-
ENSMUSG00000006782	Cnp1	met	-	-	-	CNPase	-	-	-
ENSMUSG00000034739	C1qtnf5	met	Collagen	-	Fz CUB Ldl_recept	-	-	-	-
ENSMUSG00000059327	Eda	met	Collagen	-	TNF	-	-	-	-
ENSMUSG00000039194	Rlbp1	met	-	CRAL_TRIO CRAL_	-	-	-	-	-
ENSMUSG00000014599	Csf1	met	-	-	-	-	-	-	CSF-1
ENSMUSG00000041120	Nbl1	met	-	-	DAN	-	-	-	-
ENSMUSG00000025900	Rp1h	met	-	-	DCX	-	-	-	-
ENSMUSG00000000120	Ngfr	met	-	-	Death TNFR_c6	-	-	-	-
ENSMUSG00000030341	Tnfrsf1a	met	-	-	Death TNFR_c6	-	-	-	-
ENSMUSG00000029071	Dvl1	met	DEP PDZ	-	Dishevelled DIX	-	-	-	-
ENSMUSG00000014859	E2f4	met	-	-	E2F_TDP	-	-	-	-
ENSMUSG00000027552	E2f5	met	-	-	E2F_TDP	-	-	-	-
ENSMUSG00000026124	Cfc1	met	-	EGF	-	-	-	-	-
ENSMUSG00000025809	Itgb1	met	-	EGF_2	Integrin_B_tail Int	-	-	-	-
ENSMUSG00000048915	Efna5	met	-	-	Ephrin	-	-	-	-
ENSMUSG00000002111	Sfpi1	met	-	-	Ets	-	-	-	-
ENSMUSG00000031293	Rs1h	met	F5_F8_type_C	-	-	-	-	-	-
ENSMUSG00000025810	Nrp1	met	F5_F8_type_C	-	MAM CUB	-	-	-	-
ENSMUSG00000000183	Fgf6	met	-	-	FGF	-	-	-	-
ENSMUSG00000025219	Fgf8	met	-	-	FGF	-	-	-	-
ENSMUSG00000057967	Fgf18	met	-	-	FGF	-	-	-	-
ENSMUSG00000023484	Prph1	met	-	-	Filament	-	-	Filament_head	-
ENSMUSG00000054263	Lifr	met	fn3	-	-	-	-	-	-
ENSMUSG00000038119	Cdon	met	fn3 I-set	-	ig V-set	-	-	-	-
ENSMUSG00000025158	Rfng	met	-	-	Fringe	-	-	-	-
ENSMUSG00000029570	Lfng	met	-	-	Fringe	-	-	-	-
ENSMUSG00000001761	Smo	met	-	-	Frizzled Fz	-	-	-	-
ENSMUSG00000051650	B3gnt1	met	-	-	Galactosyl_T	-	-	-	-
ENSMUSG00000025089	Gfra1	met	-	-	-	-	-	GDNF	-
ENSMUSG00000055653	Gpc3	met	-	-	Glypican	-	-	-	-
ENSMUSG00000006014	Prg4	met	-	-	Hemopexin Somat	-	-	-	-
ENSMUSG00000002633	Shh	met	-	-	Hint HH_signal	-	-	-	-
ENSMUSG00000006538	Ihh	met	-	-	Hint HH_signal	-	-	-	-
ENSMUSG00000000282	Mnt	met	-	HLH	-	-	-	-	-
ENSMUSG00000007805	Twist2	met	-	HLH	-	-	-	-	-
ENSMUSG00000020052	Ascl1	met	-	HLH	-	-	-	-	-

ENSMUSG00000028717	Tal1	met	-	HLH	-	-	-	-	-
ENSMUSG00000029553	Tcfec	met	-	HLH	-	-	-	-	-
ENSMUSG00000030543	Mesp2	met	-	HLH	-	-	-	-	-
ENSMUSG00000034701	Neurod1	met	-	HLH	-	-	-	-	-
ENSMUSG00000035799	Twist1	met	-	HLH	-	-	-	-	-
ENSMUSG00000038193	Hand2	met	-	HLH	-	-	-	-	-
ENSMUSG00000047002	Msgn1	met	-	HLH	-	-	-	-	-
ENSMUSG00000048015	Neurod4	met	-	HLH	-	-	-	-	-
ENSMUSG00000000435	Myf5	met	-	HLH	Basic	-	-	-	-
ENSMUSG00000026459	Myog	met	-	HLH	Basic	-	-	-	-
ENSMUSG00000035923	Myf6	met	-	HLH	Basic	-	-	-	-
ENSMUSG00000022528	Hes1	met	-	HLH	Hairy_orange	-	-	-	-
ENSMUSG00000023781	Hes7	met	-	HLH	Hairy_orange	-	-	-	-
ENSMUSG00000048001	Hes5	met	-	HLH	Hairy_orange	-	-	-	-
ENSMUSG00000037169	Nmyc1	met	-	HLH	-	Myc_N	-	-	-
ENSMUSG00000051910	Sox6	met	-	HMG_box	-	-	-	-	-
ENSMUSG00000063632	Sox11	met	-	HMG_box	-	-	-	-	-
ENSMUSG00000000690	Hoxb6	met	-	Homeobox	-	-	-	-	-
ENSMUSG00000001819	Hoxd13	met	-	Homeobox	-	-	-	-	-
ENSMUSG00000014603	Alx3	met	-	Homeobox	-	-	-	-	-
ENSMUSG00000014704	Hoxa2	met	-	Homeobox	-	-	-	-	-
ENSMUSG00000015579	Nkx2-5	met	-	Homeobox	-	-	-	-	-
ENSMUSG00000018973	Hoxb1	met	-	Homeobox	-	-	-	-	-
ENSMUSG00000025215	Tlx1	met	-	Homeobox	-	-	-	-	-
ENSMUSG00000026805	Barhl1	met	-	Homeobox	-	-	-	-	-
ENSMUSG00000029844	Hoxa1	met	-	Homeobox	-	-	-	-	-
ENSMUSG00000032033	Barx2	met	-	Homeobox	-	-	-	-	-
ENSMUSG00000034460	Six4	met	-	Homeobox	-	-	-	-	-
ENSMUSG00000036144	Meox2	met	-	Homeobox	-	-	-	-	-
ENSMUSG00000038692	Hoxb4	met	-	Homeobox	-	-	-	-	-
ENSMUSG00000038700	Hoxb5	met	-	Homeobox	-	-	-	-	-
ENSMUSG00000040610	TLX3_MOUSE	met	-	Homeobox	-	-	-	-	-
ENSMUSG00000048763	Hoxb3	met	-	Homeobox	-	-	-	-	-
ENSMUSG00000049604	Hoxb13	met	-	Homeobox	-	-	-	-	-
ENSMUSG00000049691	Bapx1	met	-	Homeobox	-	-	-	-	-
ENSMUSG00000058665	En1	met	-	Homeobox	-	-	-	-	-
ENSMUSG00000024619	Cdx1	met	-	Homeobox	-	-	-	Caudal_act	-
ENSMUSG00000029646	Cdx2	met	-	Homeobox	-	-	-	Caudal_act	-
ENSMUSG00000020875	Hoxb9	met	-	Homeobox	-	-	-	Hox9_act	-
ENSMUSG00000038227	Hoxa9	met	-	Homeobox	-	-	-	Hox9_act	-
ENSMUSG00000000247	Lhx2	met	-	Homeobox LIM	-	-	-	-	-

ENSMUSG00000021239	Chx10	met	-	Homeobox	OAR	-	-	-	-
ENSMUSG00000001288	Rarg	met	-	-	Hormone_recep zf	-	-	-	-
ENSMUSG00000015846	Rxra	met	-	-	Hormone_recep zf	-	-	-	-
ENSMUSG00000017491	Rarb	met	-	-	Hormone_recep zf	-	-	-	-
ENSMUSG00000021779	Thrb	met	-	-	Hormone_recep zf	-	-	-	-
ENSMUSG00000022479	Vdr	met	-	-	Hormone_recep zf	-	-	-	-
ENSMUSG00000030551	Nr2f2	met	-	-	Hormone_recep zf	-	-	-	-
ENSMUSG00000032292	Nr2e3	met	-	-	Hormone_recep zf	-	-	-	-
ENSMUSG00000037992	Rara	met	-	-	Hormone_recep zf	-	-	-	-
ENSMUSG00000046532	Ar	met	-	-	Hormone_recep zf	-	-	-	Androgen_recep
ENSMUSG00000024431	Nr3c1	met	-	-	Hormone_recep zf	-	-	GCR	-
ENSMUSG00000038086	Hspb2	met	HSP20	-	-	-	-	-	-
ENSMUSG00000025932	Eya1	met	Hydrolase	-	-	-	-	-	-
ENSMUSG00000022636	Alcam	met	-	-	ig V-set	-	-	-	-
ENSMUSG00000047319	Ttn	met	I-set	-	ig V-set	-	-	-	-
ENSMUSG00000055002	Ttn	met	I-set	-	ig V-set	-	-	-	-
ENSMUSG00000042587	Ttn	met	I-set	PPAK	ig V-set	-	-	-	-
ENSMUSG00000018593	Sparc	met	-	-	Kazal_1 Kazal_2	-	-	-	-
ENSMUSG00000021765	Fst	met	-	-	Kazal_1 Kazal_2	-	-	-	-
ENSMUSG00000055561	Spink5	met	-	-	Kazal_1 Kazal_2	-	-	-	-
ENSMUSG00000016534	Lamp2	met	-	-	Lamp	-	-	-	-
ENSMUSG00000031447	Lamp1	met	-	-	Lamp	-	-	-	-
ENSMUSG00000025784	Clec3b	met	-	-	Lectin_C	-	-	-	-
ENSMUSG00000025223	Ldb1	met	-	-	LIM_bind	-	-	-	-
ENSMUSG00000004885	Crabp2	met	Lipocalin	-	-	-	-	-	-
ENSMUSG00000046402	Rbp1	met	Lipocalin	-	-	-	-	-	-
ENSMUSG00000010721	Lmbr1	met	-	LMBR1	-	-	-	-	-
ENSMUSG00000057777	Mab21l2	met	-	-	Mab-21	-	-	-	-
ENSMUSG00000017386	Traf4	met	-	MATH zf-C3HC4	zf-TRAF	-	-	-	-
ENSMUSG00000043998	Mgat2	met	-	-	MGAT2	-	-	-	-
ENSMUSG00000024563	Smad2	met	-	-	MH2 MH1	-	-	-	-
ENSMUSG00000032402	Smad3	met	-	-	MH2 MH1	-	-	-	-
ENSMUSG00000041329	Atp1b2	met	-	-	Na_K-ATPase	-	-	-	-
ENSMUSG00000029205	Chrna9	met	-	-	Neur_chan_LBD N	-	-	-	-
ENSMUSG00000028833	Ncdn	met	-	-	Neurochondrin	-	-	-	-
ENSMUSG00000031548	Sfrp1	met	-	-	NTR Fz	-	-	-	-
ENSMUSG00000021638	Ocln	met	-	-	Occludin_ELL MAR	-	-	-	-
ENSMUSG00000019325	G6pc	met	PAP2	-	-	-	-	-	-
ENSMUSG00000001497	Pax9	met	-	-	PAX	-	-	-	-
ENSMUSG00000023951	Vegfa	met	-	-	PDGF	-	-	-	-
ENSMUSG00000028019	Pdgfc	met	-	-	PDGF CUB	-	-	-	-

ENSMUSG00000025856	Pdgfa	met	-	-	PDGF	-	-	PDGF_N	-
ENSMUSG00000029381	Shrm	met	PDZ	-	-	-	-	-	-
ENSMUSG00000030838	Ush1c	met	PDZ	-	-	-	-	-	-
ENSMUSG00000031628	Casp3	met	Peptidase_C14	-	-	-	-	-	-
ENSMUSG00000029863	Casp2	met	Peptidase_C14	-	CARD	-	-	-	-
ENSMUSG00000038894	Irs2	met	-	PH	IRS	-	-	-	-
ENSMUSG00000019969	Psen1	met	-	-	Presenilin	-	-	-	-
ENSMUSG00000028064	Sema4a	met	-	-	PSI Sema	-	-	-	-
ENSMUSG00000022105	Rb1	met	-	-	RB_B RB_A	-	-	-	-
ENSMUSG00000027641	Rbl1	met	-	-	RB_B RB_A	-	-	-	-
ENSMUSG00000031666	Rbl2	met	-	-	RB_B RB_A	-	-	-	-
ENSMUSG00000000142	Axin2	met	-	RGS	DIX	-	-	-	-
ENSMUSG00000039153	Runx2	met	-	-	Runt	-	-	-	-
ENSMUSG00000040669	Phc1	met	-	SAM_1 SAM_2	-	-	-	-	-
ENSMUSG00000022510	Trp63	met	-	SAM_2	P53	P53_tetramer	-	-	-
ENSMUSG00000028780	Sema3c	met	-	-	Sema	-	-	-	-
ENSMUSG00000028883	Sema3a	met	-	-	Sema	-	-	-	-
ENSMUSG00000020312	Shc2	met	-	SH2	PID	-	-	-	-
ENSMUSG00000021448	Shc3	met	-	SH2	PID	-	-	-	-
ENSMUSG00000020882	Cacnb1	met	SH3_1	-	Ca_channel_B	-	-	-	-
ENSMUSG00000057914	Cacnb2	met	SH3_1	-	Ca_channel_B	-	-	-	-
ENSMUSG00000036432	Siah2	met	-	-	Sina	-	-	-	-
ENSMUSG00000029050	Ski	met	-	-	Ski_Sno	-	-	-	-
ENSMUSG00000022114	Spry2	met	-	-	Sprouty	-	-	-	-
ENSMUSG00000009097	Tbx1	met	-	-	T-box	-	-	-	-
ENSMUSG00000027868	Tbx15	met	-	-	T-box	-	-	-	-
ENSMUSG00000032419	Tbx18	met	-	-	T-box	-	-	-	-
ENSMUSG00000062327	T	met	-	-	T-box	-	-	-	-
ENSMUSG00000030342	Cd9	met	-	-	Tetraspannin	-	-	-	-
ENSMUSG00000037706	Cd81	met	-	-	Tetraspannin	-	-	-	-
ENSMUSG00000021359	Tcfap2a	met	-	-	TF_AP-2	-	-	-	-
ENSMUSG00000029335	Bmp3	met	-	-	TGF_beta	-	-	-	-
ENSMUSG00000032968	Inha	met	-	-	TGF_beta	-	-	-	-
ENSMUSG00000008999	Bmp7	met	-	-	TGF_beta TGFb_p	-	-	-	-
ENSMUSG00000021253	Tgfb3	met	-	-	TGF_beta TGFb_p	-	-	-	-
ENSMUSG00000021835	Bmp4	met	-	-	TGF_beta TGFb_p	-	-	-	-
ENSMUSG00000027358	Bmp2	met	-	-	TGF_beta TGFb_p	-	-	-	-
ENSMUSG00000032179	Bmp5	met	-	-	TGF_beta TGFb_p	-	-	-	-
ENSMUSG00000037035	Inhbb	met	-	-	TGF_beta TGFb_p	-	-	-	-
ENSMUSG00000038259	Gdf5	met	-	-	TGF_beta TGFb_p	-	-	-	-
ENSMUSG00000039004	Bmp6	met	-	-	TGF_beta TGFb_p	-	-	-	-

ENSMUSG00000039239	Tgfb2	met	-	-	TGF_beta TGFb_pı	-	-	-	-	
ENSMUSG00000041324	Inhba	met	-	-	TGF_beta TGFb_pı	-	-	-	-	
ENSMUSG00000051279	Gdf6	met	-	-	TGF_beta TGFb_pı	-	-	-	-	
ENSMUSG00000022053	Ebf2	met	TIG	-	-	-	-	-	-	
ENSMUSG00000023411	Nfatc4	met	TIG	-	RHD	-	-	-	-	
ENSMUSG00000027544	Nfatc2	met	TIG	-	RHD	-	-	-	-	
ENSMUSG00000031902	Nfatc3	met	TIG	-	RHD	-	-	-	-	
ENSMUSG00000054452	Aes	met	-	-	TLE_N	-	-	-	-	
ENSMUSG00000022218	Tgm1	met	Transglut_core	-	Transglut_C Trans	-	-	-	-	
ENSMUSG00000024098	Twsg1	met	-	-	Tsg	-	-	-	-	
ENSMUSG00000022894	Adamts5	met	-	TSP_1 Reprolysin	ADAM_spacer1 Pe	-	-	-	-	
ENSMUSG00000036545	Adamts2	met	-	TSP_1 Reprolysin	ADAM_spacer1 Pe	-	-	-	-	
ENSMUSG00000019997	Ctgf	met	-	TSP_1	VWC	Cys_knot IGFBP	-	-	-	
ENSMUSG00000027351	Spred1	met	-	WH1	Sprouty	-	-	-	-	
ENSMUSG00000009900	Wnt3a	met	-	-	wnt	-	-	-	-	
ENSMUSG00000021994	Wnt5a	met	-	-	wnt	-	-	-	-	
ENSMUSG00000030093	Wnt7a	met	-	-	wnt	-	-	-	-	
ENSMUSG00000019850	Tnfaip3	met	-	zf-A20 OTU	-	-	-	-	-	
ENSMUSG00000029135	Fosl2	deu	-	bZIP_2 bZIP_1	-	-	-	-	-	
ENSMUSG00000032815	Fanca	deu	-	-	-	-	-	-	Fanconi_A	
ENSMUSG00000052305	Hbb-b1	deu	Globin	-	-	-	-	-	-	
ENSMUSG00000048482	Bdnf	deu	-	-	-	-	-	NGF	-	
ENSMUSG00000049107	Ntf3	deu	-	-	-	-	-	NGF	-	
ENSMUSG00000048616	Nog	deu	-	-	Noggin	-	-	-	-	
ENSMUSG00000029468	P2rx7	deu	-	-	-	-	-	P2X_receptor	-	
ENSMUSG00000024912	Fosl1	cor	-	bZIP_2 bZIP_1	-	-	-	-	-	
ENSMUSG00000042406	Atf4	cor	-	bZIP_2 bZIP_1	-	-	-	-	-	
ENSMUSG00000039910	Cited2	cor	-	-	-	-	CITED	-	-	
ENSMUSG00000040055	Gjb6	cor	-	-	-	Connexin	-	-	-	
ENSMUSG00000055493	Epm2a	cor	DSPc	-	-	-	-	-	-	
ENSMUSG00000047109	Cldn14	cor	-	-	PMP22_Claudin	-	-	-	-	
ENSMUSG00000021613	Hapln1	cor	-	-	V-set	-	-	Xlink	-	
ENSMUSG00000029287	Tgfbr3	cor	-	-	Zona_pellucida	-	-	-	-	
ENSMUSG00000025359	Si	ver	-	-	-	-	-	-	-	
ENSMUSG00000026241	Nppc	ver	-	-	-	-	-	ANP	-	
ENSMUSG00000027381	Bcl2l11	ver	-	-	-	-	-	-	Bim_N	
ENSMUSG00000022025	Lect1	ver	-	-	BRICHOS	-	-	-	-	
ENSMUSG00000031250	Tnmd	ver	-	-	BRICHOS	-	-	-	-	
ENSMUSG00000030669	Calca	ver	-	-	-	-	-	Calc_CGRP_IAPP	-	
ENSMUSG00000041681	Iapp	ver	-	-	-	-	-	Calc_CGRP_IAPP	-	
ENSMUSG00000021647	Cart	ver	-	-	-	-	-	CART	-	

ENSMUSG00000021367	Edn1	ver	-	-	-	-	-	Endothelin	-
ENSMUSG00000027524	Edn3	ver	-	-	-	-	-	Endothelin	-
ENSMUSG00000044042	Fmn1	ver	-	FH2	-	-	-	-	-
ENSMUSG00000030218	Mgp	ver	-	-	-	Gla	-	-	-
ENSMUSG00000063560	NP_031567.1	ver	-	-	-	Gla	-	-	-
ENSMUSG00000015812	Gnrh2	ver	-	-	-	GnRH	-	-	-
ENSMUSG00000028102	Itga10	ver	-	-	Integrin_alpha	-	-	-	-
ENSMUSG00000062991	Nrg1	ver	I-set	EGF	ig V-set	-	-	Neuregulin	-
ENSMUSG00000020660	Pomc1	ver	-	-	-	-	-	Op_neuropeptide	-
ENSMUSG00000048776	Pthlh	ver	-	-	-	-	-	Parathyroid	-
ENSMUSG00000059077	Pth	ver	-	-	-	-	-	Parathyroid	-
ENSMUSG00000025386	Pde6g	ver	-	-	-	-	-	PDE6_gamma	-
ENSMUSG00000058217	Mia1	ver	SH3_2 SH3_1	-	-	-	-	-	-
ENSMUSG00000022144	Gdnf	ver	-	-	TGF_beta	-	-	-	-
ENSMUSG00000039481	Nrtn	ver	-	-	TGF_beta	-	-	-	-
ENSMUSG00000024613	Tcof1	ver	-	-	-	-	-	-	Treacle
ENSMUSG00000056569	Mpz	ver	-	-	V-set	-	-	-	-
ENSMUSG00000030579	Tyrobp	mam	-	-	-	-	-	-	-
ENSMUSG00000038000	Acd	mam	-	-	-	-	-	-	-
ENSMUSG00000043861	Atp1b2	mam	-	-	-	-	-	-	-
ENSMUSG00000046607	Bid3	mam	-	-	-	-	-	-	-
ENSMUSG00000054006	Bcl2	mam	-	-	-	-	-	-	-
ENSMUSG00000027596	a	mam	-	-	-	-	-	Agouti	-
ENSMUSG00000029288	Ambn	mam	-	-	-	-	-	-	Amelin
ENSMUSG00000031354	Amelx	mam	-	-	-	-	-	-	Amelogenin
ENSMUSG00000004446	Bid	mam	-	-	-	-	-	-	BID
ENSMUSG00000018916	Csf2	mam	-	-	-	-	-	-	GM_CSF
ENSMUSG00000059201	Lep	mam	-	-	-	-	-	-	Leptin
ENSMUSG00000058755	Osm	mam	-	-	-	-	-	LIF_OSM	-
ENSMUSG00000053863	Mepe	mam	-	-	-	-	-	-	Osteoregulin
ENSMUSG00000019966	Kitl	mam	-	-	-	-	-	SCF	-
ENSMUSG00000026285	Pdcd1	mam	-	-	V-set	-	-	-	-
