# Supplementary material for: New genes in the evolution of the neural crest differentiation program
Source: Genome Biol. 2007 Mar 12;8(3):R36. doi: 10.1186/gb-2007-8-3-r36 (PMC1868935; doi:10.1186/gb-2007-8-3-r36)
Supplement: Additional data file 6 — A complete list of genomes of species included in this work and their respective source is compiled in this table. Abbreviations: arc (archaeobacteria), bac (bacteria), euk (eukaryota), met (metazoa), deu (deuterostomia), cor (chordata), ver (vertebrata). [file gb-2007-8-3-r36-S6.pdf]

group	name	sequences	source
arc	Aeropyrum pernix, K1	2694 pep	cogent241:APER-XK1-01
	Archaeoglobus fulgidus, DSM4304	2409 pep	cogent241:AFUL-DSM-01
	Haloarcula marismortui, ATCC43049	4240 pep	cogent241:HMAR-502-01
	Halobacterium sp., NRC-1	2605 pep	cogent241:HALO-NRC-01
	Methanobacterium thermoautotrophicum, deltaH	1871 pep	cogent241:MTHE-DEL-01
	Methanococcus jannaschii, DSM 2661	1773 pep	cogent241:MJAN-DSM-01
	Methanococcus maripaludis, S2	1722 pep	cogent241:MMAR-XS2-01
	Methanopyrus kandleri, AV19	1687 pep	cogent241:MKAN-AV1-01
	Methanosarcina acetivorans, C2A	4528 pep	cogent241:MACE-C2A-01
	Methanosarcina mazei, Go1	3371 pep	cogent241:MMAZ-GO1-01
	Nanoarchaeum equitans, Kin4-M	563 pep	cogent241:NEQU-N4M-01
	Picrophilus torridus, DSM9790	1535 pep	cogent241:PTOR-790-01
	Pyrobaculum aerophilum, IM2	2605 pep	cogent241:PAER-IM2-01
	Pyrococcus abyssi, GE5	1765 pep	cogent241:PABY-GE5-01
	Pyrococcus furiosus, DSM3638	2065 pep	cogent241:PFUR-638-01
	Pyrococcus horikoshii, OT3	2061 pep	cogent241:PHOR-OT3-01
	Sulfolobus solfataricus, P2	2996 pep	cogent241:SSOL-XP2-01
	Sulfolobus tokodaii, str. 7	2826 pep	cogent241:STOK-XX7-01
	Thermococcus kodakaraensis, KOD1	2306 pep	cogent241:TKOD-OD1-01
	Thermoplasma acidophilum, DSM1728	1478 pep	cogent241:TACI-DSM-01
	Thermoplasma volcanium, GSS1	1526 pep	cogent241:TVOL-GSS-01
bac	Acinetobacter calcoaceticus, ADP1	3325 pep	cogent241:ACIN-DP1-01
	Agrobacterium tumefaciens, C58	5299 pep	cogent241:ATUM-C58-01
	Anabaena sp., strain PCC 7120	6129 pep	cogent241:NOST-PCC-01
	Anaplasma marginale, St. Maries	949 pep	cogent241:AMAR-MAR-01
	Aquifex aeolicus, VF5	1553 pep	cogent241:AAEO-VF5-01
	Azoarcus sp. EbN1	4598 pep	cogent241:AZOA-BN1-01
	Bacillus anthracis, Ames	5311 pep	cogent241:BANT-AME-01
	Bacillus cereus, ATCC 10987	5603 pep	cogent241:BCER-987-01
	Bacillus cereus, ATCC 14579	5255 pep	cogent241:BCER-579-01
	Bacillus halodurans, C-125	4066 pep	cogent241:BHAL-C12-01
	Bacillus licheniformis, ATCC 14580	4161 pep	cogent241:BLIC-580-01
	Bacillus licheniformis, DSM13	4196 pep	cogent241:BLIC-M13-01
	Bacillus subtilis, 168	4093 pep	cogent241:BSUB-168-01
	Bacteroides fragilis, NCTC9343	4236 pep	cogent241:BFRA-343-01
	Bacteroides fragilis, YCH46	4625 pep	cogent241:BFRA-H46-01
	Bacteroides thetaiotaomicron, VPI-5482	4778 pep	cogent241:BTHE-VPI-01
	Bartonella henselae, Houston-1	1488 pep	cogent241:BHEN-HOU-01
	Bartonella quintana, Toulouse	1142 pep	cogent241:BQUI-TOU-01
	Bdellovibrio bacteriovorus, HD100	3583 pep	cogent241:BBAC-100-01
	Bifidobacterium longum, NCC2705	1729 pep	cogent241:BLON-NCC-01
	Blochmannia floridanus	583 pep	cogent241:BFLO-XXX-01
	Bordetella bronchiseptica, NCTC-13252	4994 pep	cogent241:BBRO-252-01
	Bordetella parapertussis, NCTC-13253	4185 pep	cogent241:BPAR-253-01
	Bordetella pertussis, NCTC-13251	3447 pep	cogent241:BPER-251-01
	Borrelia burgdorferi, B31	1639 pep	cogent241:BBUR-B31-01
	Borrelia garinii, PBi	932 pep	cogent241:BGAR-PBI-01
	Bradyrhizobium japonicum, USDA110	8317 pep	cogent241:BJAP-USD-01
	Brucella abortus, 9-941	3085 pep	cogent241:BABO-941-01

Brucella melitensis, M16	3198 pep	cogent241:BMEL-M16-01
Brucella suis, str. 1330	3293 pep	cogent241:BSUI-133-01
Buchnera aphidicola, Bp	504 pep	cogent241:BAPH-XBP-01
Buchnera aphidicola, SG	545 pep	cogent241:BAPH-XSG-01
Buchnera sp., APS	575 pep	cogent241:BUCH-APS-01
Burkholderia mallei, ATCC 23344	4764 pep	cogent241:BMAL-344-01
Burkholderia pseudomallei, K96243	5729 pep	cogent241:BPSE-243-01
Campylobacter jejuni, NCTC 11168	1634 pep	cogent241:CJEJ-NCT-01
Campylobacter jejuni, RM1221	1838 pep	cogent241:CJEJ-221-01
Caulobacter crescentus, CB15	3737 pep	cogent241:CCRE-XXX-01
Chlamydia pneumoniae, AR39	1119 pep	cogent241:CPNE-AR3-01
Chlamydia pneumoniae, CWL029	1052 pep	cogent241:CPNE-CWL-01
Chlamydia pneumoniae, J138	1070 pep	cogent241:CPNE-J13-01
Chlamydia trachomatis, MoPn	921 pep	cogent241:CTRA-MOP-01
Chlamydia trachomatis, serovar D	894 pep	cogent241:CTRA-SVD-01
Chlamydophila abortus, S26/3	932 pep	cogent241:CABO-263-01
Chlamydophila caviae, GPIC	1005 pep	cogent241:CCAV-GPI-01
Chlorobium tepidum, TLS	2252 pep	cogent241:CTEP-TLS-01
Chromobacterium violaceum, ATCC 12472	4407 pep	cogent241:CVIO-472-01
Clostridium acetobutylicum, ATCC 824	3916 pep	cogent241:CACE-ATC-01
Clostridium perfringens, str. 13	2723 pep	cogent241:CPER-X13-01
Clostridium tetani, E88	2432 pep	cogent241:CTET-E88-01
Corynebacterium diphtheriae, NCTC13129	2272 pep	cogent241:CDIP-129-01
Corynebacterium efficiens, YS314T	2950 pep	cogent241:CEFF-YS3-01
Corynebacterium glutamicum	3040 pep	cogent241:CGLU-XXX-01
Coxiella burnetii, RSA 493	2045 pep	cogent241:CBUR-RSA-01
Dehalococcoides ethenogenes, 195	1580 pep	cogent241:DETH-195-01
Deinococcus radiodurans, R1	3116 pep	cogent241:DRAD-XR1-01
Desulfotalea psychrophila, LSv54	3236 pep	cogent241:DPSY-V54-01
Desulfovibrio vulgaris, str. Hildenborough	3514 pep	cogent241:DVUL-HIL-01
Ehrlichia ruminantium (Welgevonden), str. ARC	888 pep	cogent241:ERUM-ARC-01
Enterococcus faecalis ,V583	3113 pep	cogent241:EFAE-V58-01
Erwinia carotovora subsp. atroseptica, SCRI1043	4472 pep	cogent241:ECAR-043-01
Escherichia coli 0157:H7, RIMD0509952	5447 pep	cogent241:ECOL-RIM-01
Escherichia coli, CFT073	5379 pep	cogent241:ECOL-CFT-01
Escherichia coli, MG1655	4290 pep	cogent241:ECOL-MG1-01
Escherichia coli O157:H7, EDL933	5349 pep	cogent241:ECOL-EDL-01
Francisella tularensis, SCHU S4	1603 pep	cogent241:FTUL-XS4-01
Fusobacterium nucleatum, ATCC 25586	2067 pep	cogent241:FNUC-ATC-01
Geobacillus kaustophilus, HTA426	3540 pep	cogent241:GKAU-426-01
Geobacter sulfurreducens, PCA	3445 pep	cogent241:GSUL-PCA-01
Gloeobacter violaceus, PCC 7421	4430 pep	cogent241:GVIO-421-01
Gluconobacter oxydans, 621H	2664 pep	cogent241:GOXY-21H-01
Haemophilus influenzae, KW20	1707 pep	cogent241:HINF-KW2-01
Helicobacter hepaticus, ATCC51449	1875 pep	cogent241:HHEP-449-01
Helicobacter pylori, 26695	1575 pep	cogent241:HPYL-266-01
Helicobacter pylori, J99	1491 pep	cogent241:HPYL-J99-01
Idiomarina loihiensis, DSM 15497T	2628 pep	cogent241:ILOI-497-01
Lactobacillus acidophilus, NCFM (ATCC 700396)	1864 pep	cogent241:LACI-396-01
Lactobacillus johnsonii, NCC 533	1821 pep	cogent241:LJOH-533-01
Lactobacillus plantarum, WCFS1	3009 pep	cogent241:LPLA-WCF-01

	Lactococcus lactis, IL1403	2266 pep	cogent241:LLAC-IL1-01
	Legionella pneumophila, Philadelphia-1	2942 pep	cogent241:LPNE-PHI-01
	Legionella pneumophila, strain Lens	2934 pep	cogent241:LPNE-LEN-01
	Legionella pneumophila, strain Paris	3166 pep	cogent241:LPNE-PAR-01
	Leifsonia xyli subsp. xyli, CTCB07	2030 pep	cogent241:LXYL-B07-01
	Leptospira interrogans, L1-130	3660 pep	cogent241:LINT-130-01
	Leptospira interrogans, str. 56601	4727 pep	cogent241:LINT-566-01
	Listeria innocua, CLIP 11262	2968 pep	cogent241:LINN-CLI-01
	Listeria monocytogenes, EGD-e	2846 pep	cogent241:LMON-EGD-01
	Listeria monocytogenes, F2365	2821 pep	cogent241:LMON-365-01
	Listeria monocytogenes, F6854	2973 pep	cogent241:LMON-854-01
	Listeria monocytogenes, H7858	3109 pep	cogent241:LMON-858-01
	Mannheimia succiniciproducens, MBEL55E	2384 pep	cogent241:MSUC-55E-01
	Mesorhizobium loti, MAFF303099	7281 pep	cogent241:MLOT-MAF-01
	Methylococcus capsulatus (Bath)	2959 pep	cogent241:MCAP-BAT-01
	Mycobacterium bovis, AF2122/97	3920 pep	cogent241:MBOV-AF2-01
	Mycobacterium leprae, TN	1605 pep	cogent241:MLEP-XTN-01
	Mycobacterium tuberculosis, CDC1551	4203 pep	cogent241:MTUB-CDC-01
	Mycobacterium tuberculosis, H37Rv	3924 pep	cogent241:MTUB-H37-01
	Mycoplasma gallisepticum, Rlow	726 pep	cogent241:MGAL-RLO-01
	Mycoplasma genitalium, G-37	479 pep	cogent241:MGEN-G37-01
	Mycoplasma hyopneumoniae, strain 232	691 pep	cogent241:MHYO-232-01
	Mycoplasma mobile, 163K	635 pep	cogent241:MMOB-63K-01
	Mycoplasma mycoides subsp. mycoides SC strain PG1	1016 pep	cogent241:MMYC-G1T-01
	Mycoplasma penetrans, HF2	1037 pep	cogent241:MPEN-HF2-01
	Mycoplasma pneumoniae, M129	689 pep	cogent241:MPNE-M12-01
	Mycoplasma pulmonis, UAB CTIP	782 pep	cogent241:MPUL-UAB-01
	Neisseria meningitidis, MC58	2081 pep	cogent241:NMEN-MC5-01
	Neisseria meningitidis, Z2491	2065 pep	cogent241:NMEN-Z24-01
	Nitrosomonas europaea, ATCC19718	2461 pep	cogent241:NEUR-718-01
	Nocardia farcinica, IFM 10152	5936 pep	cogent241:NFAR-152-01
	Oceanobacillus iheyensis, HET831	3496 pep	cogent241:OIHE-HET-01
	Parachlamydia sp., UWE25	2031 pep	cogent241:PCHL-E25-01
	Pasteurella multocida, Pm70	2014 pep	cogent241:PMUL-PM7-01
	Photorhabdus luminescens, TTO1	4683 pep	cogent241:PLUM-TO1-01
	Phytoplasma asteris, OY	754 pep	cogent241:PAST-XOY-01
	Pirellula sp., strain 1	7325 pep	cogent241:PIRE-ST1-01
	Porphyromonas gingivalis, W83	1909 pep	cogent241:PGIN-W83-01
	Prochlorococcus marinus, MED4	1712 pep	cogent241:PMAR-MED-01
	Prochlorococcus marinus, MIT9313	2265 pep	cogent241:PMAR-MIT-01
	Prochlorococcus marinus, SS120	1882 pep	cogent241:PMAR-SS1-01
	Propionibacterium acnes, KPA171202	2297 pep	cogent241:PACN-202-01
	Pseudomonas aeruginosa, PAO1	5570 pep	cogent241:PAER-PAO-01
	Pseudomonas putida, KT2440	5387 pep	cogent241:PPUT-KT2-01
	Pseudomonas syringae pv. tomato, DC3000	5471 pep	cogent241:PSYR-DC3-01
	Ralstonia solanacearum	5116 pep	cogent241:RSOL-XXX-01
	Rhodopseudomonas palustris, CGA009	4814 pep	cogent241:RPAL-009-01
	Rickettsia conorii, str. Malish 7	1374 pep	cogent241:RCON-MAL-01
	Rickettsia prowazekii, Madrid E	834 pep	cogent241:RPRO-MAD-01
	Rickettsia typhi, ATCC VR-144	838 pep	cogent241:RTYP-144-01

	Salmonella enterica (Paratyphi A), ATCC 9150	4093 pep	cogent241:SENT-PAR-01
	Salmonella enterica serovar Typhi, CT18	4767 pep	cogent241:SENT-CT1-02
	Salmonella enterica serovar Typhimurium, LT2	4553 pep	cogent241:SENT-LT2-01
	Salmonella enterica sv. Choleraesuis, SC-B67	4666 pep	cogent241:SENT-B67-01
	Salmonella enterica, Ty2	4323 pep	cogent241:SENT-TY2-01
	Shewanella oneidensis, MR1	4867 pep	cogent241:SONE-MR1-01
	Shigella flexneri, 2457T	4068 pep	cogent241:SFLE-457-01
	Shigella flexneri (serotype 2a), str. 301	4452 pep	cogent241:SFLE-301-01
	Silicibacter pomeroyi, DSS-3	4252 pep	cogent241:SPOM-SS3-01
	Sinorhizobium meliloti, strain 1021	6206 pep	cogent241:SMEL-102-01
	Staphylococcus aureus, COL	2618 pep	cogent241:SAUR-COL-01
	Staphylococcus aureus, MRSA252	2656 pep	cogent241:SAUR-252-01
	Staphylococcus aureus MRSA, MW2	2659 pep	cogent241:SAUR-MW2-01
	Staphylococcus aureus MRSA, N315	2624 pep	cogent241:SAUR-N13-01
	Staphylococcus aureus, MSSA476	2579 pep	cogent241:SAUR-476-01
	Staphylococcus aureus VRSA, Mu50	2748 pep	cogent241:SAUR-MU5-01
	Staphylococcus epidermidis, RP62A	2526 pep	cogent241:SEPI-62A-01
	Streptococcus agalactiae, 2603 V/R	2144 pep	cogent241:SAGA-260-01
	Streptococcus agalactiae, NEM316	2094 pep	cogent241:SAGA-NEM-01
	Streptococcus mutans, UA159	1960 pep	cogent241:SMUT-UA1-01
	Streptococcus pneumoniae, R6	2043 pep	cogent241:SPNE-XR6-01
	Streptococcus pneumoniae, TIGR4	2140 pep	cogent241:SPNE-TIG-01
	Streptococcus pyogenes M18, MGAS8232	1845 pep	cogent241:SPYO-MGA-01
	Streptococcus pyogenes M1, SF370	1696 pep	cogent241:SPYO-SF3-01
	Streptococcus pyogenes M3, MGAS315	1865 pep	cogent241:SPYO-XM3-01
	Streptococcus pyogenes M3, SSI-1	1861 pep	cogent241:SPYO-SSI-01
	Streptococcus pyogenes, MGAS10394	1886 pep	cogent241:SPYO-394-01
	Streptococcus thermophilus, CNRZ 1066	1915 pep	cogent241:STHE-066-01
	Streptococcus thermophilus, LMG18311	1889 pep	cogent241:STHE-311-01
	Streptomyces avermitilis	7671 pep	cogent241:SAVE-XXX-01
	Streptomyces coelicolor, A3(2)	7810 pep	cogent241:SCOE-A32-01
	Symbiobacterium thermophilum, IAM 14863	3337 pep	cogent241:SYTH-863-01
	Synechococcus sp., WH8102	2517 pep	cogent241:SYCC-WH8-01
	Synechocystis sp., PCC6803	3167 pep	cogent241:SYNE-PCC-01
	Thermoanaerobacter tengcongensis, MB4	2588 pep	cogent241:TTEN-MB4-01
	Thermosynechococcus elongatus, BP-1	2475 pep	cogent241:TELO-BP1-01
	Thermotoga maritima, MSB8	1849 pep	cogent241:TMAR-MSB-01
	Thermus thermophilus, HB27	2210 pep	cogent241:TTHE-B27-01
	Treponema denticola, ATCC 35405	2767 pep	cogent241:TDEN-405-01
	Treponema pallidum, Nichols	1030 pep	cogent241:TPAL-NIC-01
	Tropheryma whipplei, TW08/27	783 pep	cogent241:TWHI-TW0-01
	Tropheryma whipplei, Twist	808 pep	cogent241:TWHI-TWI-01
	Ureaplasma urealyticum, serovar 3	613 pep	cogent241:UURE-SV3-01
	Vibrio cholerae, El Tor N16961	3835 pep	cogent241:VCHO-N16-01
	Vibrio fischeri, ES114	3802 pep	cogent241:VFIS-114-01
	Vibrio parahaemolyticus, RIMD2210633	4832 pep	cogent241:VPAR-RIM-01
	Vibrio vulnificus, YJ016	5028 pep	cogent241:VVUL-YJ0-01
	Wigglesworthia glossinidia brevipalpis	611 pep	cogent241:WGLO-BRE-01
	Wolbachia pipientis, wMel	1176 pep	cogent241:WPIP-WME-01
	Wolbachia sp., TRS	805 pep	cogent241:WOLB-TRS-01
	Wolinella succinogenes strain DSM 1740	2044 pep	cogent241:WSUC-740-01

	Xanthomonas axonopodis pv. citri, str. 306	4427 pep	cogent241:XAXO-306-02
	Xanthomonas campestris pv. campestris, 8004	4273 pep	cogent241:XCAM-004-01
	Xanthomonas campestris pv. campestris, ATCC33913	4181 pep	cogent241:XCAM-AT3-01
	Xanthomonas oryzae pv. oryzae, KAXCC10331	4637 pep	cogent241:XORY-331-01
	Xylella fastidiosa, 9a5c	2830 pep	cogent241:XFAS-9A5-01
	Xylella fastidiosa, PD	2036 pep	cogent241:XFAS-XPD-01
	Yersinia pestis bv Medievalis, 91001	4142 pep	cogent241:YPES-001-01
	Yersinia pestis, CO92	4093 pep	cogent241:YPES-CO9-01
	Yersinia pestis, KIM	4281 pep	cogent241:YPES-KIM-01
	Yersinia pseudotuberculosis, IP32953	4038 pep	cogent241:YPSE-953-01
	Zymomonas mobilis (ZM4), ATCC31821	1998 pep	cogent241:ZMOB-ZM4-01
euk	Plasmodium falciparum, 3D7	5295 pep	cogent241:PFAL-3D7-01
	Saccharomyces cerevisiae, S288C	6357 pep	cogent241:SCER-S28-01
	Schizosaccharomyces pombe	4945 pep	cogent241:SPOM-XXX-01
met	Anopheles gambiae, PEST	15101 pep	cogent241:AGAM-PES-01
	Caenorhabditis briggsae	19507 pep	cogent241:CBRI-XXX-01
	Drosophila melanogaster	18484 pep	cogent241:DMEL-XXX-02
deu	Strongylocentrotus Purpuratus, mRNA	527735 ests	GenBank, NCBI
cor	Ciona intestinalis [JGI 1.95]	21574 pep	ensembl_v31
	Branchiostoma, mRNA	321472 ests	GenBank, NCBI
ver	Takifugu rubripes [Fugu 2.0]<br>Danio rerio [WTSI Zv5]<br>Tetraodon nigroviridis [TETRAODON 7]	33003 pep<br>32143 pep<br>28005 pep	ensembl_v32
Blast sources			
mouse	Mus musculus [NCBIM33]	23658 pep	ensembl_v31
ciona	Ciona_intestinalis [JGI1.95]	21574 pep	ensembl_v31
danio	Danio_rerio [ZFISH4]	32062 pep	ensembl_v31
gallus	Gallus_gallus [WASHUC1]	28416 pep	ensembl_v31
xenopus	Xenopus_tropicalis [JGI3]	52786 pep	ensembl_v31

Links:

Cogent: http://cgg.ebi.ac.uk/services/cogent/
GenBank at NCBI: http://www.ncbi.nlm.nih.gov/
Ensembl: http://www.ensembl.org
