# Supplementary material for: New genes in the evolution of the neural crest differentiation program
Source: Genome Biol. 2007 Mar 12;8(3):R36. doi: 10.1186/gb-2007-8-3-r36 (PMC1868935; doi:10.1186/gb-2007-8-3-r36)

	Mouse	Gallus	Xenopus	Danio
Total genes	23658	28416	52786	32062
Prokaryota hits	7320	10015	23312	12086
Eukaryota hits	4764	6214	9988	7445
Metazoa hits	5516	6156	11920	8434
Deuterostomia hits	987	921	1411	1126
Chordata hits	595	585	807	758
Vertebrata hits	1720	1669	1764	2133
Vertebrata no hits	2756	2856	3584	80

	Mouse	Gallus	Xenopus	Danio
Prokaryota hits	30,9%	35,2%	44,2%	37,7%
Eukaryota hits	20,1%	21,9%	18,9%	23,2%
Metazoa hits	23,3%	21,7%	22,6%	26,3%
Deuterostomia hits	4,2%	3,2%	2,7%	3,5%
Chordata hits	2,5%	2,1%	1,5%	2,4%
Vertebrata hits	7,3%	5,9%	3,3%	6,7%
Vertebrata no hits	11,6%	10,1%	6,8%	0,2%

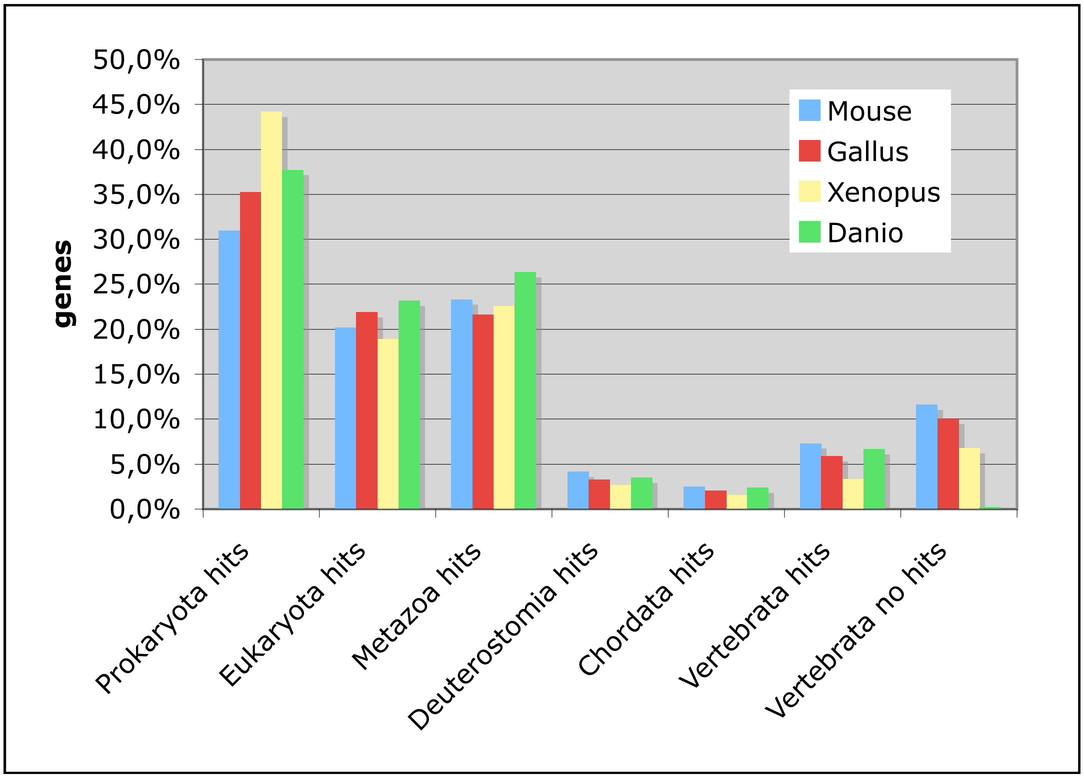

Supplement: Additional data file 7 — As a control of our gene phylogeny analysis, we also run the sequential blast pipeline using other vertebrate groups, namely (chicken, xenopus and zebrafish genomes). The tables show the number or percentage of genes assigned to each evolutionary category. The graphical representation of the gene phylogeny for the four vertebrate species analyzed revealed a very similar gene loss/emergence profile. [file gb-2007-8-3-r36-S7.pdf]
